# Supplementary material for: Behavioral changes during the COVID-19 pandemic decreased income diversity of urban encounters
Source: Nat Commun. 2023 Apr 21;14:2310. doi: 10.1038/s41467-023-37913-y (PMC10120472; doi:10.1038/s41467-023-37913-y)
Supplement: Supplementary file 1 — Supplementary Information [file 41467_2023_37913_MOESM1_ESM.pdf]

Supplementary Information for

Behavioral changes during the COVID-19 pandemic decreased income diversity in urban encounters

Takahiro Yabe[1,*], Bernardo García Bulle Bueno[1], Xiaowen Dong[2,3], Alex Pentland[1,3], Esteban Moro[1,3,4,*]

[1]Institute for Data, Systems, and Society, Massachusetts Institute of Technology, Cambridge, MA 02139, USA
[2]Department of Engineering Science, University of Oxford, Oxford OX2 6ED, UK
[3]Media Lab, Massachusetts Institute of Technology, Cambridge, MA 02139, USA
[4]Grupo Interdisciplinar de Sistemas Complejos (GISC), Departamento de Matemáticas, Universidad Carlos III de Madrid, 28911 Leganés, Madrid, Spain
*Corresponding authors: tyabe@mit.edu, emoro@mit.edu

Supplementary Notes

1 Mobility data **6**
1.1 Home estimation and stop detection . . . 6
1.2 Robustness to threshold distance for attribution of stays to places . . . 6
1.3 Robustness against choice of POI dataset . . . 9
1.4 Robustness against definition of income quantiles . . . 10
1.5 Robustness against choice of data filtering parameters . . . 12

2 Data representativeness **14**
2.1 Population and income representativeness . . . 15
2.2 Robustness check via post-stratification . . . 17
2.3 Accuracy of mobility data in high density areas . . . 17

3 Income diversity of encounters **19**
3.1 Income diversity at places . . . 19
3.2 Income diversity experienced by individuals . . . 23
3.3 Other measure of diversity: entropy . . . 26

4 Counterfactual simulations **29**
4.1 Synthetic data generation procedure . . . 29
4.2 Analysis of the impacts of removal rates under different scenarios . . . 32
4.3 Summary of counterfactual simulation results . . . 35
4.4 Parameters of the Social-EPR model . . . 35

5 Explaining spatial heterogeneity in diversity **39**

5.1 Regression models and results 39

5.2 Pooled model with monthly fixed effects 50

6 COVID-19 intensity and segregation **56**

6.1 Model estimation results 57

6.2 Robustness of results via time series modeling 57

7 Software **60**

List of Figures

S1 Sensitivity of place based diversity of encounters with respect to different spatial parameters used for the visit attribution algorithm 7
S2 Robustness of income diversity at food and grocery places to using different POI datasets 10
S3 Dollar ranges of median household income values when using different number of income quantiles ($n = \{2, 3, 4, 5, 6\}$). 11
S4 Sensitivity of income diversity of encounters with respect to number of income quantile categories n used to compute income diversity. 12
S5 Sensitivity of income diversity of encounters with respect to the selection of users based on different thresholds for minimum observation times. 13
S6 Diversity of sociodemographic, political, and climate characteristics of the four cities compared with the 15 largest CBSAs. 15
S7 Sensitivity of income diversity of encounters with respect to the representativeness of mobile phone users across CBGs and income groups 16
S8 % of POIs in high density areas and the proportion of dwell time spent at those high density POIs, in each city. 19
S9 Total dwell time (left column) and place diversity (right column) of each place category are consistent when we remove high density POIs ($\geq$ 3 POIs within 20 meters) from the dataset. 20
S10 Total dwell time (left column) and place diversity (right column) of each place category are consistent when we refine the visit attribution to high density POIs ($\geq$ 3 POIs within 20 meters) using the Foursquare checkin dataset. 21
S11 Income diversity of encounters in places in the three CBSAs 22
S12 Percentage difference of income diversity at places in Boston, compared to same months in 2019. 23
S13 Average income diversity of encounters at different place categories in the three CBSAs 24
S14 Time series data of the normalized visits per user to different place categories observed in each month 25
S15 Absolute values of the income diversity of encounters experienced at places and by individuals across time for the four CBSAs. 25
S16 Box plots of place and individual income diversity across time in all four different cities. Using the mean (green triangle) and the median (orange line) values does not substantially affect the results. 27
S17 Comparison of the diversity and entropy metrics. 28
S18 Income diversity of encounters measured using the entropy metric. 28
S19 Comparison of counterfactual scenarios 30
S20 Retain rates used to generate mobility datasets under different counterfactual scenarios. 31
S21 (a) Histograms of $\tau_{q \in \{q_1, q_2, q_3, q_4\}}$ for counterfactual scenarios (i) and (ii-1). (b) Differences in the distributions of τ_q between counterfactual scenarios (i) and (ii-1) are significant for each income quantile, but are nearly identical when aggregated across all income quantiles, yielding similar income diversity measures. 33
S22 Retain rates for different place taxonomies 34
S23 Percentage changes in income diversity of encounters in places and by individuals in the four CBSAs under different synthetic counterfactual scenarios. 36

S24 Proportion of percentage changes in income diversity of encounters in places and by individuals in the four CBSAs under different synthetic counterfactual scenarios. . . 37
S25 Key parameters of the Social-EPR model, ρ and γ (shown in panel a), and π (shown in panel b), are fairly consistent during the pandemic. The social exploration parameter σ_s (shown in main manuscript Figure 2D) was the only parameter with significant changes. Data are presented as mean values $+$ / $-$ SEM. 38
S26 The model parameter with the most significant change during the pandemic was the social exploration parameter σ_s, even when compared with the counterfactual simulation results. 39
S27 Changes in proportion of high-frequency visitation to place subcategories across different periods of the pandemic in (a) Seattle, (b) Los Angeles, and (c) Dallas (Boston is shown in main manuscript). 40
S28 ΔD_{CBG} for different time periods in (a) Seattle, (b) Los Angeles, and (c) Dallas. Maps were produced in Python using the TIGER shapefiles from the U.S. Census Bureau [28] 41
S29 Correlation between D_{CBG} in different timings during the pandemic and the corresponding months in 2019. 42
S30 Correlation matrix of CBG based residential variables. 43
S31 Monthly fixed effects for the pooled model with D_{CBG} as the dependent variable. Data are presented as mean values $+$ / $-$ SEM ($n = 427,776$ POIs across the four cities). 51
S32 Root mean squared error of the pooled regression model with D_{CBG} as the dependent variable by months. 53
S33 Proportion of explained variance across the three groups of variables in the pooled model with D_{CBG} as the dependent variable. 53
S34 Monthly fixed effects for the pooled model with ΔD_{CBG} as the dependent variable. Data are presented as mean values $+$ / $-$ SEM ($n = 427,776$ POIs across the four cities). 53
S35 Root mean squared error of the pooled regression model with ΔD_{CBG} as the dependent variable by months. 55
S36 Proportion of explained variance across the three groups of variables in the pooled model with ΔD_{CBG} as the dependent variable. 55
S37 Number of monthly COVID-19 cases (top row), COVID-19 deaths (middle row), and stringency index (bottom row) in each of the CBSAs. 56
S38 Reduction in income diversity regressed against the stringency index and the death rates due to COVID-19 in each of the CBSAs. 58
S39 Autocorrelation and partial autocorrelation of original series and 1st order of differencing of $\Delta D_{CBSA}(t)$ for (a) Boston, (b) Seattle, (c) Los Angeles, and (d) Dallas. Error bands for autocorrelation and partial autocorrelation show the 95% confidence intervals. 61

List of Tables

S1 Number of places in the Foursquare dataset in the four core-based statistical areas (CBSAs) analyzed in this study. 8

S2	Description of the four core-based statistical areas (CBSAs) analyzed in this study.	14
S3	Summary statistics of the residential variables used in the regression models.	44
S4	Regression results for D_{CBG} for April 2019. The statistical tests were two-sided.	45
S5	Regression results for D_{CBG} for April 2020. The statistical tests were two-sided.	46
S6	Regression results for D_{CBG} for October 2021. The statistical tests were two-sided.	47
S7	Regression results for ΔD_{CBG} for April 2020. The statistical tests were two-sided.	48
S8	Regression results for ΔD_{CBG} for May 2020. The statistical tests were two-sided.	49
S9	Regression results for ΔD_{CBG} for December 2020. The statistical tests were two-sided.	50
S10	Regression results for ΔD_{CBG} for January 2021. The statistical tests were two-sided.	51
S11	Regression results for D_{CBG}, all months pooled. The statistical tests were two-sided.	52
S12	Regression results for ΔD_{CBG}, all months pooled. The statistical tests were two-sided.	54
S13	Regression results for $\Delta D_{CBSA}(t)$ using COVID-19 intensity and policy measures. The statistical tests were two-sided.	59
S14	Regression results for $\Delta D_{CBSA}(t)$ using only COVID-19 local deaths and policy strictness measures. The statistical tests were two-sided.	59
S15	Augmented Dickey Fuller test for $\Delta D_{CBSA}(t)$.	60
S16	ARIMA regression results for $\Delta D_{CBSA}(t)$ using COVID-19 local deaths and policy strictness measures. The statistical tests were two-sided.	62

1 Mobility data

1.1 Home estimation and stop detection

In this study we utilize an anonymized location dataset of mobile phones and smartphone devices provided by Spectus Inc., a location data intelligence company which collects anonymous, privacy-compliant location data of mobile devices using their software development kit (SDK) technology in mobile applications and ironclad privacy framework. Spectus processes data collected from mobile devices whose owners have actively opted in to share their location, and require all application partners to disclose their relationship with Spectus, directly or by category, in the privacy policy. With this commitment to privacy, the data set contains location data for roughly 15 million daily active users in the United States. Through Spectus' Data for Good program, Spectus provides mobility insights for academic research and humanitarian initiatives. All data analyzed in this study are aggregated to preserve privacy[1]. Each entry in the data table comprises anonymized device ID, location coordinates, start time, and dwell time of the stop for the device.

To define the type of location (Home or Work), different variables are used, including the number of days spent in a given location in the last month, the daily average number of hours spent in that location, and the time of the day spent in the location (nighttime/daytime). To estimate the home position of a user, the algorithm combines the three variables and creates a score that represents the probability that the position points to the home. The more days and the average number of hours spent in the position, the higher the score is. Higher scores will also be assigned to the most common places during the night. The location that maximizes this score is defined as the home of the device.

Once the location of the home location is identified, the algorithm looks for the work position. Note that the algorithm requires the work location to be located at least 100 meters apart from the home location. The same variables used for the detection of the home location are used, but a higher score is given to daytime locations for the work location rather than nighttime locations. Spectus runs the algorithm every week in order to confirm or update the inferred home and work locations as we observe new data. We will only consider devices that have been present in Spectus' dataset for at least 15 days. Spectus tightly restricts access to the inferred precise home and work locations of devices. Furthermore, it is used as input into various downstream processes to create more privacy-protected versions of Spectus datasets. For example, we only expose home and work datasets in Spectus Workbench associated with standard Census Block Groups, created by the U.S. Census Bureau, rather than the precise locations. This offers a good balance between utility and privacy: according to the U.S. Census Bureau, there are between 600 and 3000 people living in each block group. Each block group is an aggregate of contiguous U.S. blocks sharing similar socio-demographic characteristics. The representativeness of this data has been tested and corrected in Section 2 in the Supplementary Material. The stops, which are location clusters where individual users stay for a given duration, are estimated using the Sequence Oriented Clustering approach [30].

1.2 Robustness to threshold distance for attribution of stays to places

To measure the diversity of physical encounters in urban environments, we attribute the stops of individual users to specific places in the city. To study the stops at different places, we use stops that are longer than 10 minutes but shorter than four hours. In our study, we use location data of places

[1] `https://spectus.ai/privacy/privacy-policy/`

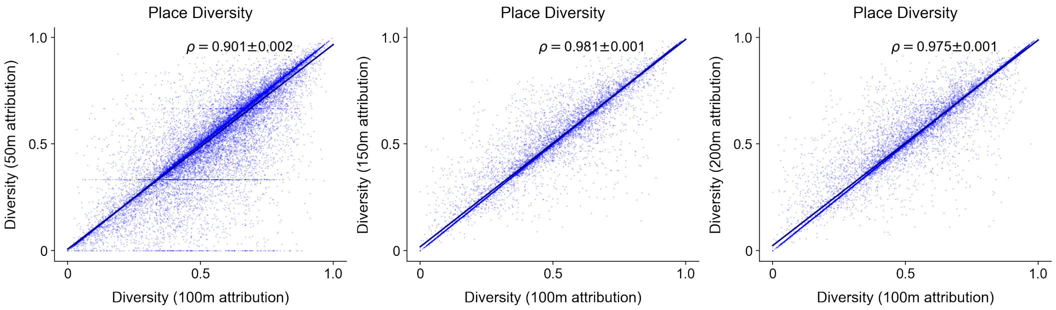

(a) Boston CBSA

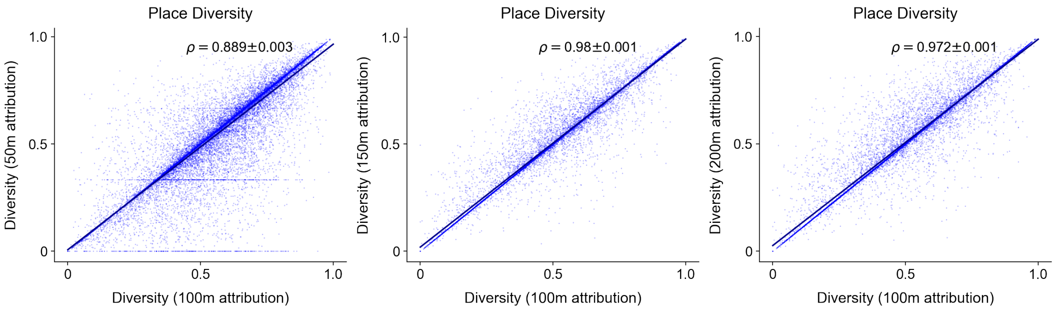

(b) Seattle CBSA

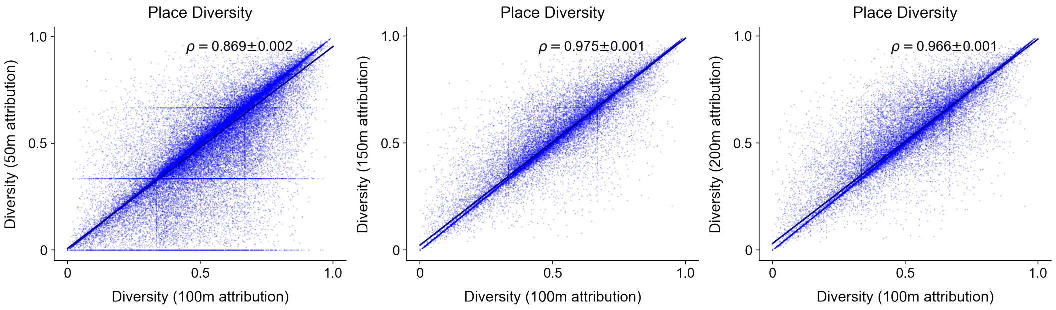

(c) Los Angeles CBSA

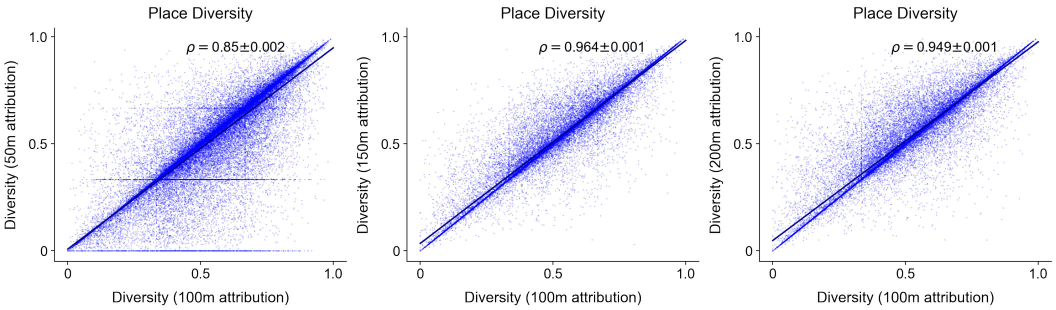

(d) Dallas CBSA

Figure S1: Sensitivity of place based diversity of encounters with respect to different spatial parameters (50m, 100m, 150m, 200m) used for the visit attribution algorithm, for (a) Boston, (b) Seattle, (c) Los Angeles, and (d) Dallas. For all values $d_{max} = \{50, 150, 200\}$, the Pearson correlation of place based diversity metrics are extremely high. This robustness check shows that the estimated diversity values do not depend on the choice of the spatial threshold parameter for visit attribution.

Table S1: Number of places in the Foursquare dataset in the four core-based statistical areas (CBSAs) analyzed in this study.

	CBSAs			
Place category	Boston	Seattle	Los Angeles	Dallas
Arts and Museums	3,346	2,797	12,019	4,340
City and Outdoors	9,370	6,794	22,364	9,719
Coffee and Tea	872	1,968	3,284	1,064
Entertainment	5,548	3,991	18,533	7,065
Food	14,791	10,936	46,411	22,812
Grocery	2,017	1,166	4,602	1,808
Health	318	209	979	554
Service	20,500	15,692	53,972	30,044
Shopping	8,612	6,134	26,538	14,269
Transportation	6,615	7,460	18,165	5,538
All places	71,989	57,147	206,867	97,213

collected via the Foursquare API [2]. To protect the users' privacy, we have removed various privacy-sensitive places from our places database. Sensitive places include health-related places, places where the vulnerable population are located, military-related, religious facilities, places that are related to sexual-orientation, and adult-oriented places [3]. As a result, we have a total of 71K places in Boston, 57K places in Seattle, 206K places in Los Angeles, and 97K places in Dallas. The breakdown of the number of places by the place category is shown in Table S1. 'City and Outdoor' places include parks, public spaces, fields, beaches, and historic sites. To attribute a stop to a place, we simply attribute each stop the closest place in our dataset. The average spatial error of a collection of observations during a stay is 8.31 meters, which allows us to detect the specific place category of where the individual is staying. This is achieved by using multiple GPS observations during the stay (which is 10 minutes to four hours in our study) to improve the accuracy of the stay location. To avoid attributing a stop to place far away, we attribute the stop to a place within $d_{max} = 100$ meters from the observed location of the stop. If the stop is further away than 100 meters from any place in the dataset, the stop is discarded from our dataset and not used for computing the diversity of encounters. These values of d_{max} are maximum distances in which a stay location may be attributed to the closest POI, thus the visit attribution algorithm adaptively chooses the closest POI from the stay location based on the density of the area. The threshold parameter only dictates the maximum distance to which the stay location can be assigned to a POI, and this should not differ across high-dense or low-dense areas. We have note that those robustness checks were implemented on the same data for pre-pandemic income experienced diversity [20] or epidemiological studies of the transmission of COVID-19 [2]. In all cases, it was found that the results were robust towards those thresholds.

The robustness of our results on the diversity of encounters have been tested using different spatial thresholds of d_{max}. Different levels of d_{max} could change how individuals' stays are attributed to the places, and thus could affect our estimates of the income diversity of physical encounters.

[2] https://developer.foursquare.com/

[3] https://spectus.ai/privacy/spoi-policy/

Figure S1 compares the diversity of encounters for places in the four CBSAs when we use different levels of d_{max} (y-axis) with our default parameter $d_{max} = 100m$ (x-axis). For all values $d_{max} = \{50, 150, 200\}$, the Pearson correlation of place based diversity metrics are extremely high, where for Boston, $\rho[D_\alpha^{d_{max}=100m}, D_\alpha^{d_{max}=50m}] = 0.901 \pm 0.002$, $\rho[D_\alpha^{d_{max}=100m}, D_\alpha^{d_{max}=150m}] = 0.981 \pm 0.001$, and $\rho[D_\alpha^{d_{max}=100m}, D_\alpha^{d_{max}=200m}] = 0.975 \pm 0.001$. This robustness check shows that the estimated diversity values do not depend on the choice of the spatial threshold parameter for visit attribution.

1.3 Robustness against choice of POI dataset

Although we may assume that our dataset of places (name, location coordinates, business category) collected via the Foursquare API is relatively comprehensive, there could be places that are missing from the dataset, which could affect our results on income diversity. To check whether our findings in our study are independent on the selection of the dataset of places, we used the "ReferenceUSA Business Historical Data", which is a record of companies across the US. The dataset is created annually from Infogroup's U.S. Business Database, and a snapshot of the data is saved each December (we used the 2020 version). The data, similar to the Foursquare data, contains the company name, mailing address, SIC and NAICS codes, employee size, sales volume, latitude/longitude, and other variables about each company [13]. However, Infogroup data is limited to mainly business locations, and the spatial granularity is also limited to the official address level, thus there are instances where different places are clustered into the exact same location if located in the same shopping mall. Sometimes the location of the official address of the mall is at the gate, located far away from any POI in the mall.

On the other hand, Foursquare data contains location data of different types of POIs such as parks, and also contains the precise location coordinates of the POIs, which is why the Foursquare POI is used as the main dataset in this study. For example, in Quincy Market in Boston, which is one of the most attractive shopping and dining spots for tourists in the city, Infogroup data has 1 location coordinate containing information of 20 points of interest, while in the Foursquare data, each place is assigned a unique coordinate within the shopping mall.

We also note that Foursquare data does not only come from voluntary input. The data is built from a combination of crowd-sourced user activity and the aggregation of data from additional sources and has third-party verification [7]. Finally, a 2018 study comparing the Foursquare POI database with other public POI databases from mapping and social media platforms (Facebook, Foursquare, Google, Instagram, OSM, Twitter, and Yelp) established that while none of these databases is complete, Foursquare's data quality, as measured by number of POI, number of categories included, and positioning accuracy, was among the best [10].

In Boston, there were 12641 food and restaurant places (NAICS code starts with 722) and 3886 grocery stores (NAICS code starts with 445) in the ReferenceUSA dataset, compared to the 14,791 and 2,017 places in the Foursquare data, respectively. The income diversity experienced at food, restaurant, and grocery places were calculated using the two different datasets for several time periods (April and October in 2019, 2020, 2021). Figure S2 shows the mean $\pm$ standard errors of income diversity of encounters at places. Despite the differences in the number of places and the minor differences in category labels between the Foursquare data and the ReferenceUSA datasets, similar levels of decrease in income diversity are observed between the two datasets, suggesting the results we obtain are robust against the choice of place datasets.

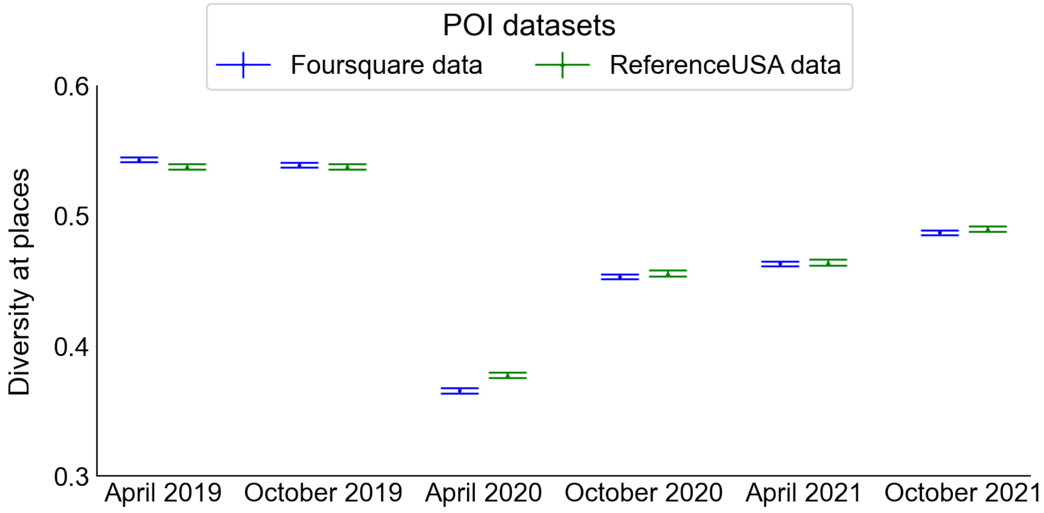

Figure S2: Robustness of income diversity at food and grocery places to using different POI datasets (Foursquare and ReferenceUSA data). Horizontal bars show the standard errors, which are very small due to the large number of places. Despite the differences in the number of places and the minor differences in category labels between the Foursquare data and the ReferenceUSA datasets, similar levels of decrease in income diversity are observed between the two datasets, suggesting the results we obtain are robust against the choice of place datasets ($n = 16,248$ POIs for Foursquare, $n = 12,334$ for ReferenceUSA). Data are presented as mean values $+$ / $-$ SEM.

1.4 Robustness against definition of income quantiles

To estimate the socioeconomic status of each individual, we use the median household income of the census block group (CBG) where their estimated homes are located in as a proxy for their income. Note that we don't use the actual estimation of the household income by CBG made by the American Community Survey [3], but only a broad categorization of them by quantiles. Thus our income classification is only slightly affected by uncertainty problems in the actual estimations [26]. We also note that our classification of different CBGs in a small set of income groups using the median household income is consistent with a large body of literature [20, 29, 18, 4, 2, 14, 15, 22].

Individuals in our dataset are then grouped into four equal-size quantiles of economic status within each city. The diversity of encounters at places and for individuals are hereon calculated using these assigned quantile values. For Boston, the median household income thresholds for the quantile classification are: [$0,$59K] for quantile 1 (low income), [$59K,$84K] for quantile 2 (medium-low income), [$84K, $108K] for quantile 3 (medium-high income), and [$108K, $250K] for quantile 4 (high income). The four income quantile ranges for the four cities are shown in Figure S3a. While Boston has the highest quantile thresholds, Los Angeles and Dallas have slightly lower income quantile ranges.

Since our estimation of income diversity is conducted by grouping the encountered individuals into income quantile groups and measuring the unevenness of the group sizes, it is important to check whether our income diversity estimates are affected by the number of income quantile groups we use. This is especially important given the uncertainties in the estimation of household incomes in the American Community Survey (ACS) [26].

To check the robustness of our income diversity measures against the selection of the number of

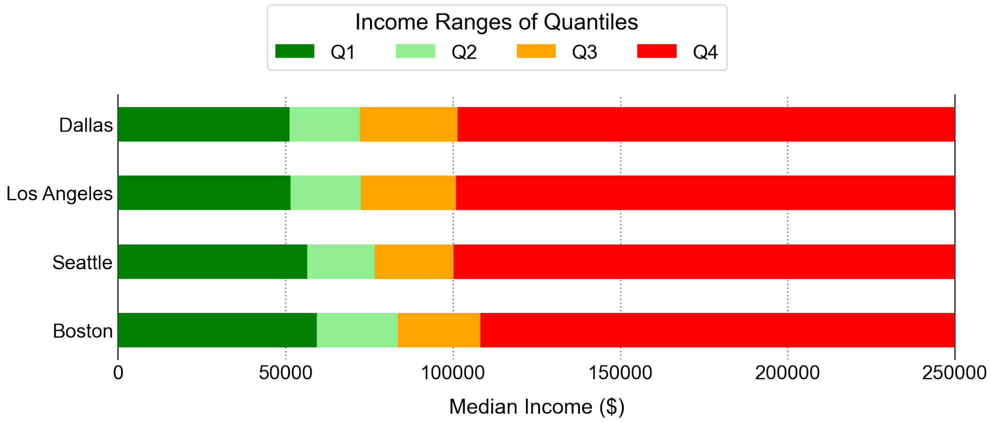

(a) Dollar ranges of the four income quantiles for the four CBSAs.

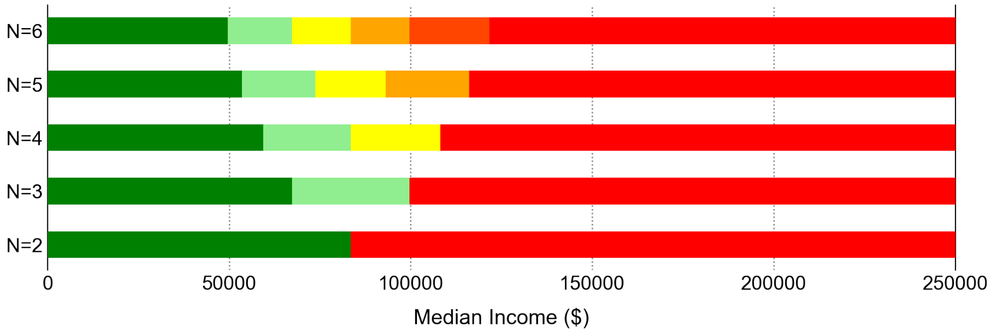

(b) Dollar ranges of income when different number of income quantiles are used ($n = \{2, 3, 4, 5, 6\}$) in the Boston CBSA.

Figure S3: (a) Dollar ranges of median household income values when using 4 income quantiles, and (b) when using different number of income quantiles ($n = \{2, 3, 4, 5, 6\}$).

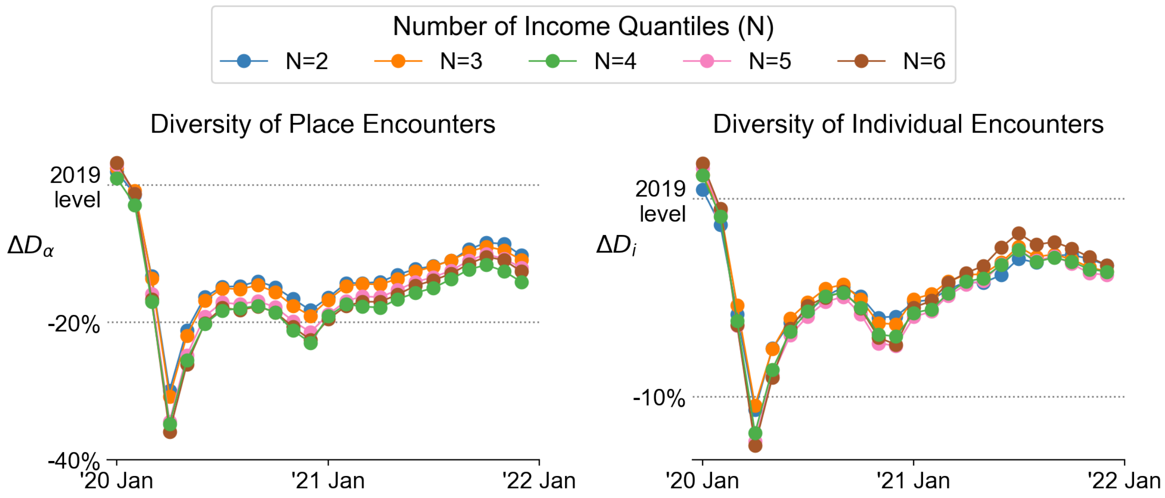

Figure S4: Sensitivity of income diversity of encounters with respect to number of income quantile categories used. The results on the dynamics of income diversity are robust and independent of the choice of the number of income quantile categories n.

income quantiles n, we compute the place-based and individual-based diversity measures when using different number of income quantiles ($n = 2, 3, 4, 5, 6$). The income diversity metric under a given n is computed as the following:

$$D_\alpha^{\{n\}} = 1 - \frac{n}{2n-2} \sum_{q=1}^{n} |\tau_{q\alpha} - \frac{1}{n}|, \tag{1}$$

where n is the number of quantiles used for income quantile classification. For Boston's case, the income ranges of quantiles under different number of quantiles are shown in Figure S3b. Figure S4 shows the estimated decrease in diversity in Boston when using different number of income quantiles. We observe that both the dynamics of the diversity of encounters experienced at places and by individuals are consistent across time, showing high agreement with the result obtained using $n = 4$ (green color). Therefore, we conclude that our findings related to the loss of diversity in the short- and long-term during the pandemic is independent of the choice of the number of quantiles.

1.5 Robustness against choice of data filtering parameters

Since mobile phone location pings are collected via various smartphone apps at asynchronous timings and frequencies, some users are observed for a long duration during the day while others could be observed for just a very short period of time. Using a group of individuals with very short observation times could skew the results of the income diversity of encounters. Therefore, we limit the group of individual users analyzed in this study to those who are observed a substantial amount of time each day. In this study, we use users who are observed more than $t_{min} = 300$ minutes across all visited places (including their homes) to select the users used in our analysis.

Since 300 minutes is an arbitrary temporal threshold, we tested whether income diversity experienced at places and by individuals are affected by the selection of the t_{min} parameter. There is an obvious trade-off between the number of available users in the dataset and the temporal coverage of

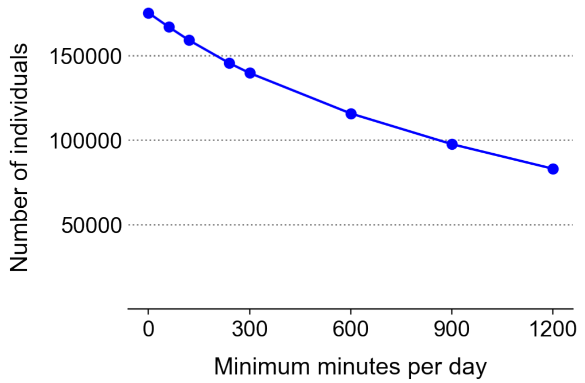

(a) Number of smartphone users selected under different thresholds for minimum minutes observed per day.

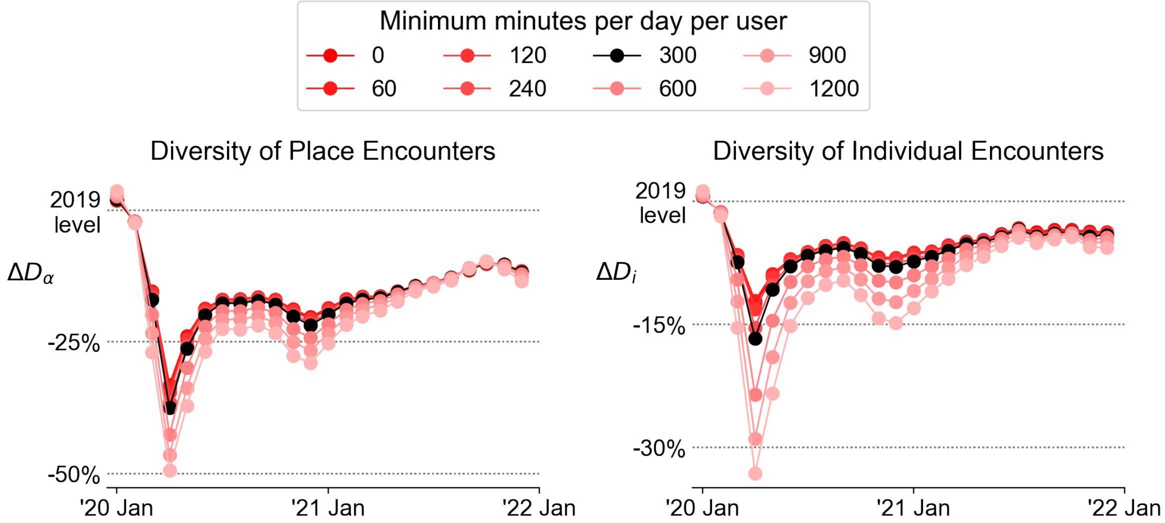

(b) Sensitivity of income diversity of encounters with respect to the minimum observation threshold for user selection. The decrease in diversity becomes amplified when selecting a smaller set of users with longer observed duration.

Figure S5: (a) Sensitivity of income diversity of encounters with respect to the selection of users based on different thresholds for minimum observation times. In this study, we use users who are observed more than $t_{min} = 300$ minutes across all visited places (including their homes) to select the users used in our analysis. (b) This sensitivity analysis shows that the main takeaways of the dynamics in income diversity are consistent across different threshold parameters.

Table S2: Description of the four core-based statistical areas (CBSAs) analyzed in this study.

CBSA	Population	# users (monthly)	# stays (monthly)	# places
Boston-Cambridge-Newton	4.64M	144K	2.34M	71,989
Seattle-Tacoma-Bellevue	3.55M	141K	2.23M	57,147
Los Angeles-Long Beach-Anaheim	13.05M	452K	10.00M	206,867
Dallas-Fort Worth-Arlington	6.70M	425K	8.83M	97,213
Total	27.94M	1.16M	23.4M	433,216

the users' mobility patterns, as shown in Figure S5a for the Boston CBSA. Out of all the 175K users in the dataset, 140K users were observed more than 300 minutes.

Figure S5b shows how the income diversity dynamics experienced at places (left panel) and by individuals (right panel) vary when using different t_{min} parameters. The losses in diversity in encounters are amplified for both places and individuals when we employ a stricter threshold for selecting the users, mainly due to the lack of individuals visiting each place, which increases the likelihood of lower diversity. However, the main takeaways of the dynamics in income diversity are consistent – the income diversity in urban encounters have become decreased in both the long and short term, both from the places' and individuals' perspectives.

To summarize the mobility data filtering process, we 1) estimate home and stop locations for each individual, 2) attribute the stays to specific places, 3) estimate each individual user's socioeconomic status using census-block group level data, and 4) select users who are observed more than 300 minutes per day. After pre-processing the mobility datasets for each of the four urban areas, the entire dataset contains a total of 1.16 million unique users and 23.4 million stays across a total of 97K places. Table S2 shows the summary statistics for the four CBSAs.

2 Data representativeness

The location data used in our study is collected from smartphones via various apps and services. Although a significant portion (85% according to 2021 data[4]) of the US population owns a smartphone, one could question the representativeness of the 1.16 million user samples across geographical regions and income quantiles. Studies have reported digital divide and smartphone usage gaps across sociodemographic groups in the US [27]. In this section, we test whether our group of users in the mobility data are representative of the total population, and further employ post-stratification techniques to correct for any potential biases in the sampling rates across places and socioeconomic status and to test whether the results on income diversity dynamics are robust to such uncertainties concerning data representativeness.

In this study, we selected Boston, Seattle, Los Angeles, and Dallas as the four CBSAs given constraints on data collection. The four cities were selected with respect to the diversity of characteristics in terms of geographical locations, sociodemographic details, political inclinations, weather characteristics, COVID-19 policy strictness. To quantitatively show the diversity of the four cities' characteristics, we plot various sociodemographic, geographic, COVID-19 related, and climate related variables

[4] https://www.statista.com/topics/2711/us-smartphone-market/\#topicHeader__wrapper

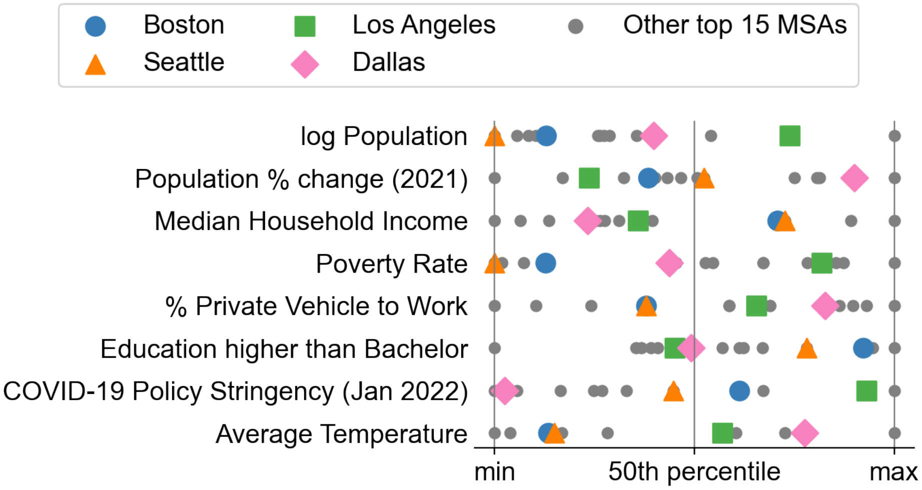

Figure S6: Diversity of sociodemographic, political, and climate characteristics of the four cities shown by comparing with the largest 15 metropolitan statistical areas in the US. The plot shows that the selected four cities are diverse in various characteristics including population dynamics, wealth, transportation, COVID-19 policies, and climate.

of the four cities among the top 15 metropolitan statistical areas in the US. More specifically, Figure S6 shows the the population (log), population % change from 2020 to 2021, median household income, poverty rates, % of workers who use private vehicles to work, % population with education level with Bachelor degree (all using the American Community Survey), the COVID-19 stringency index in 2022 January provided by the University of Oxford, and annual average temperature of the four cities compared against the largest 15 metropolitan statistical areas in the US. The figure shows that our collection of four cities covers a wide range of values for each of the sociodemographic, political (COVID-19 stringency), and climate characteristics.

2.1 Population and income representativeness

The sampling percentage of the mobility data (100% $\times$ number of observed mobile phone users divided by the total population from the census data) is around 3% across all metropolitan regions. To test whether the users in the location data are representative of the entire population, first we compare the population detection in our mobility data and the 2016-2020 5-year American Community Survey (ACS) data for each of the CBGs in the cities. The left panel in Figure S7a shows the comparison between the census population (x-axis) and the number of observed smartphone users (y-axis) on the CBG scale in the month of January 2020 in the Boston CBSA. The correlation is moderately high ($\rho = 0.767$) showing that despite the use of such small census areas and potential bias in the smartphone usage patterns, we are able to obtain a good representation of the population. This correlation is relatively stable before and during the pandemic at around $\rho = 0.75$, which is moderately high. In Section 2.2 we use post-stratification techniques to correct for any differences in the sample percentages across CBGs and assess whether our estimates on income diversity of urban encounters are affected by the representativeness of the data.

In addition to the differences in sampling rates across CBGs, differences in representativeness across income quantiles are important for our study. To study the representativeness across income

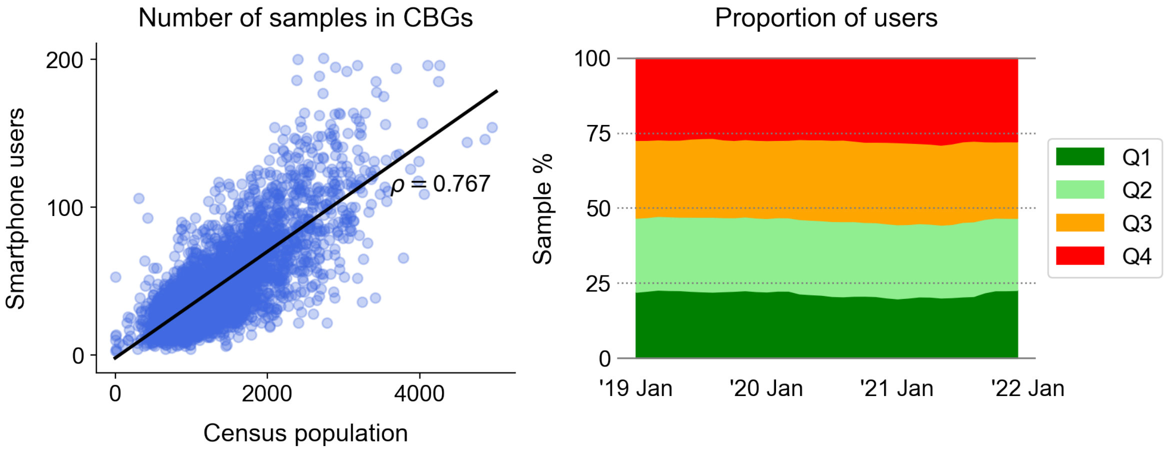

(a) (left) Comparison of number of smartphone users with the census population. (right) Proportion of smartphone users in the four income quantiles.

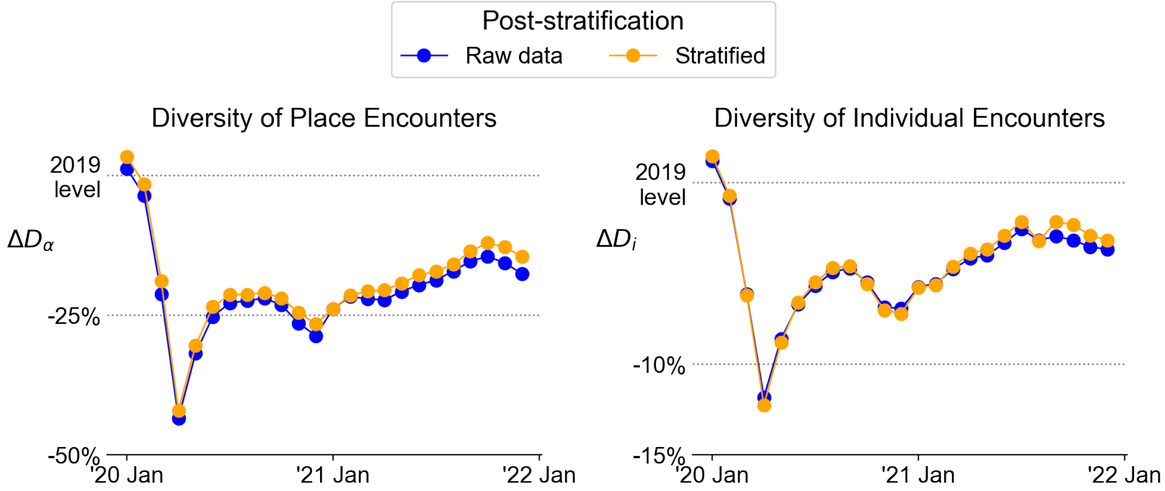

(b) Sensitivity of income diversity of encounters with respect to the representativeness of smartphone location data via post-stratification.

Figure S7: (a) Sensitivity of income diversity of encounters with respect to the representativeness of mobile phone users across CBGs and income groups. (b) Overall, the results show that even though the representativeness of mobile phone users are not perfect, the effect on our estimations are very limited.

quantiles, we compute the proportion of users in the four income quantiles across time, which is shown in the right panel of Figure S7a. A completely balanced dataset would have all income quantiles each represent 25% of the proportion of the users. However, we can observe that the highest income quantile (Q4) is over-represented in the dataset throughout the 3 years period, while the lowest income quantile (Q1) is under-represented. In Section 2.2, we investigate whether this bias in income representativeness affects our estimates on income diversity using post-stratification techniques.

2.2 Robustness check via post-stratification

To understand the effects of the varying sampling rates across CBGs and income groups on our estimation on income diversity of urban encounters experienced at places and by individuals, we apply a post-stratification technique, which is used in a previous study [20]. Post-stratification is a well know sampling tool [23] and is typically used to study the impact of sampling biases in mobile phone location data [15] or (geolocated) social media data [29] on various downstream tasks and analyses. Following the methods employed in Moro et al. [20], we denote w_g the expansion factor, which is the ratio of the population of census block g to the population detected in our mobility data. We then weight the time people from census block group g spends at place α by

$$\hat{\tau}_{g\alpha} = w_g \tau_{g\alpha} \tag{2}$$

where the assumption is that $\tau_{g\alpha}$ is proportional to the number of people visiting the place. Using this method, we could increase (decrease) the time spent at places by people coming from census block groups that are under-estimated (over-estimated).

Recomputing the income diversity of urban encounters using the corrected duration of stays $\hat{\tau}_{g\alpha}$, as shown in Figure S7b we observe that the dynamics of the income diversity decrease between the raw mobility data and the post-stratified data are very similar. These results show the robustness of the insights on income diversity, and that even though the representativeness of mobile phone users are not perfect, the effect on our estimations are very limited.

2.3 Accuracy of mobility data in high density areas

Although the spatial errors of mobile phone GPS location signals (around 10 meters) are relatively small compared to the distance between neighboring POIs, such uncertainty may be problematic when analyzing mobility patterns in high density urban areas, especially with many high-rise buildings. During the last years, the suitability of the specific mobility dataset used in this study to investigate different urban behaviors has been extensively tested. The data has been used in different contexts and lately for COVID-19 research, and different aspects of the data have been validated, from its population representativeness, visit attribution, and even travel behavior. For example in previous works [20] and subsequent works [12, 2, 1], it has been shown that the dataset is highly representative of the different socio-demographic groups in the urban areas considered. Even more interestingly, the results about changes in visitation patterns to different places during the pandemic (e.g. groceries or food places, see [2] and [1]) and subsequent infections coincide with other works based on different datasets, surveys and epidemiological data (see those papers for references). Even the results about decrease of walking behavior detected from this dataset coincide with Apple or Google mobility trends [12]. Since Spectus provided access to this dataset through their Data for Good initiative we are not the only group using this dataset and other research groups, agencies and governments have used it

and validated it in many other different contexts (see e.g., World Bank blog on use of Spectus data for pandemic response [5]).

There may be spatial noise that could affect the visit attribution process in high-density mixed land-use areas such as the Prudential Center in Boston, even with the 10m granularity accuracy of mobility data. Unfortunately, due to limitations in ground truth data, it is infeasible to validate the actual visit counts to individual stores, however, a previous study checked the representativeness of the same dataset at the level of major sports venues, including games in the National Football League (NFL), National Basketball Association (NBA), and National Hockey League (NHL), and showed high accuracy in estimating the attendance for each game, validated using official statistics. While this validation was conducted on large-scale venues, accurate estimations for NBA and NHL venues in urban locations (which is high-density and mixed use), such as TD Garden in Boston, provide strong evidence that the mobile phone location data may be used to accurately estimate visitation patterns to places (see Supplementary Material for [20]).

To address this issue further, we have investigated the effect of the visit attribution in high-density areas. We find that such high density POIs, which have 5 or more POIs located within 20 meters including vertical alignment (which a generous spatial threshold relative to the GPS location error of 10 meters), account for around 5% of POIs and 2% of total dwell time spent, averaged across the four cities. Figure S8 shows the % of high density POIs and the % of total dwell time spent in those locations, under different parameters for determining high density POIs (3, 5, 10, or 20 POIs located within 20 meters).

To investigate whether this issue does not affect the overall results on the income diversity of urban encounters, we recomputed the results using two methods: 1) compute the diversity at places without the inclusion of such high density POIs, and 2) refine our visit attribution algorithm for such high density POIs using Foursquare check-in data. For method 1), we remove all POIs that have more than 2 POIs located within 20 meters (corresponding to the first data point in Figure S8), which correspond to around 7% (Dallas) to 14% (Los Angeles) of all POIs. The plots on the left column of Figure S9 shows the total dwell time spent at each category in the actual data (x-axis) against the data where high density POIs are removed (y-axis). The colors correspond to the place major categories. In all four cities, the correlation between the two values are extremely high ($R > 0.99$), indicating that removing such high density POIs does not significantly change the distribution of visits and dwell times. The plots on the left column of Figure S9 shows the average place diversity at each category in the actual data (x-axis) against the data where high density POIs are removed (y-axis). In all four cities, the correlation between the two values are high ($R > 0.98$), indicating that removing such high density POIs does not significantly change the estimation of place diversity in our study. The only POI category which was significantly affected by removing high density POIs in Boston (blue point which decreases place diversity from around 0.65 to 0.5 when high density POIs are removed) was Science Museums. While removing the high density POIs has insignificant effects on all the other place categories, we further conduct an additional experiment to test whether refining the visit attribution in high density POI areas using an external dataset (Foursquare checkins) affects our results on income diversity.

For method 2), we attribute visits to all high density POIs (3 or more POIs located within 20 meters) by using Foursquare checkin data as a proxy of actual visitation patterns. When attributing visits, the visit is assigned probabilistically to a POI proportionate to its number of Foursquare check-ins. This way, in high density POIs areas we give more probability to visit those places which are

[5] https://blogs.worldbank.org/sustainablecities/poor-people-respond-differently-stay-home-orders-

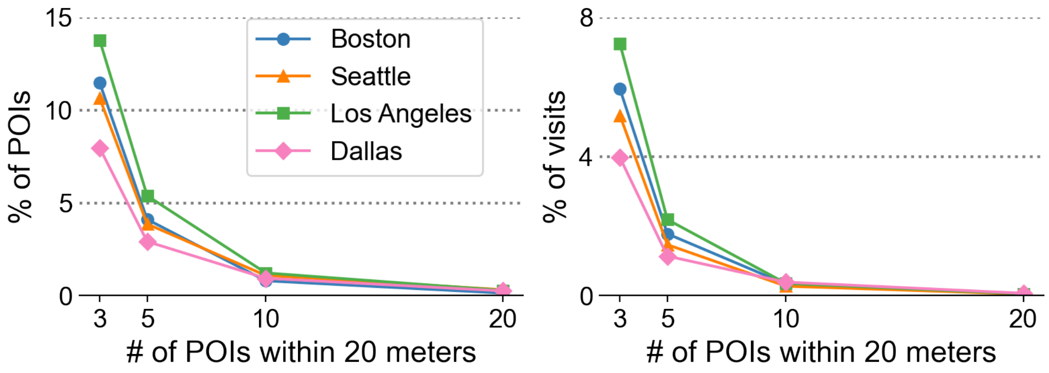

Figure S8: % of POIs in high density areas (i.e., have more than [3,5,10,20] POIs within 20 meters), and the proportion of dwell time spent at those high density POIs, in each city. Analysis shows that even though visit estimations in high density areas could be an issue for mobile phone location data, the proportions of such high density areas are small ($\sim 5\%$ of POIs have 5 or more POIs within 20 meters).

more popular. The plots on the left column of Figure S10 shows the total dwell time spent at each category in the actual data (x-axis) against the data where high density POIs are reattributed (y-axis). The colors correspond to the place major categories. In all four cities, the correlation between the two values are extremely high ($R > 0.995$), indicating that reattibuting the visits of such high density POIs does not significantly change the distribution of visits and dwell times. The plots on the left column of Figure S10 shows the average place diversity at each category in the actual data (x-axis) against the data where high density POIs are reattributed (y-axis). In all four cities, the correlation between the two values are high ($R > 0.975$), indicating that reattributing such high density POIs does not significantly change the estimation of place diversity in our study. In both methods and in all four cities, we found the results on income diversity to be robust against the treatment of high density POIs, under different threshold parameters.

3 Income diversity of encounters

3.1 Income diversity at places

To measure the income diversity of encounters experienced at each place α in each city, we compute the proportion of total time spent at place α by each income quantile q, $\tau_{q\alpha}$. Income thresholds for the quantiles are chosen based on the income distributions in each city, as described in Section 1.4. We also checked that the results for income diversity are independent of the choice of the number of income quantiles in Section 1.4. We define full diversity of encounters at a place when people from all income quantiles spend the same amount of time, $\tau_{q\alpha} = \frac{1}{4}$ for all q. Using the metric used to compute income segregation in urban encounters in previous studies [20], we define the income diversity experienced at each place α, D_α as a measure of evenness of time spend by different income quantiles:

$$D_\alpha = 1 - \frac{2}{3} \sum_q |\tau_{q\alpha} - \frac{1}{4}|. \quad (3)$$

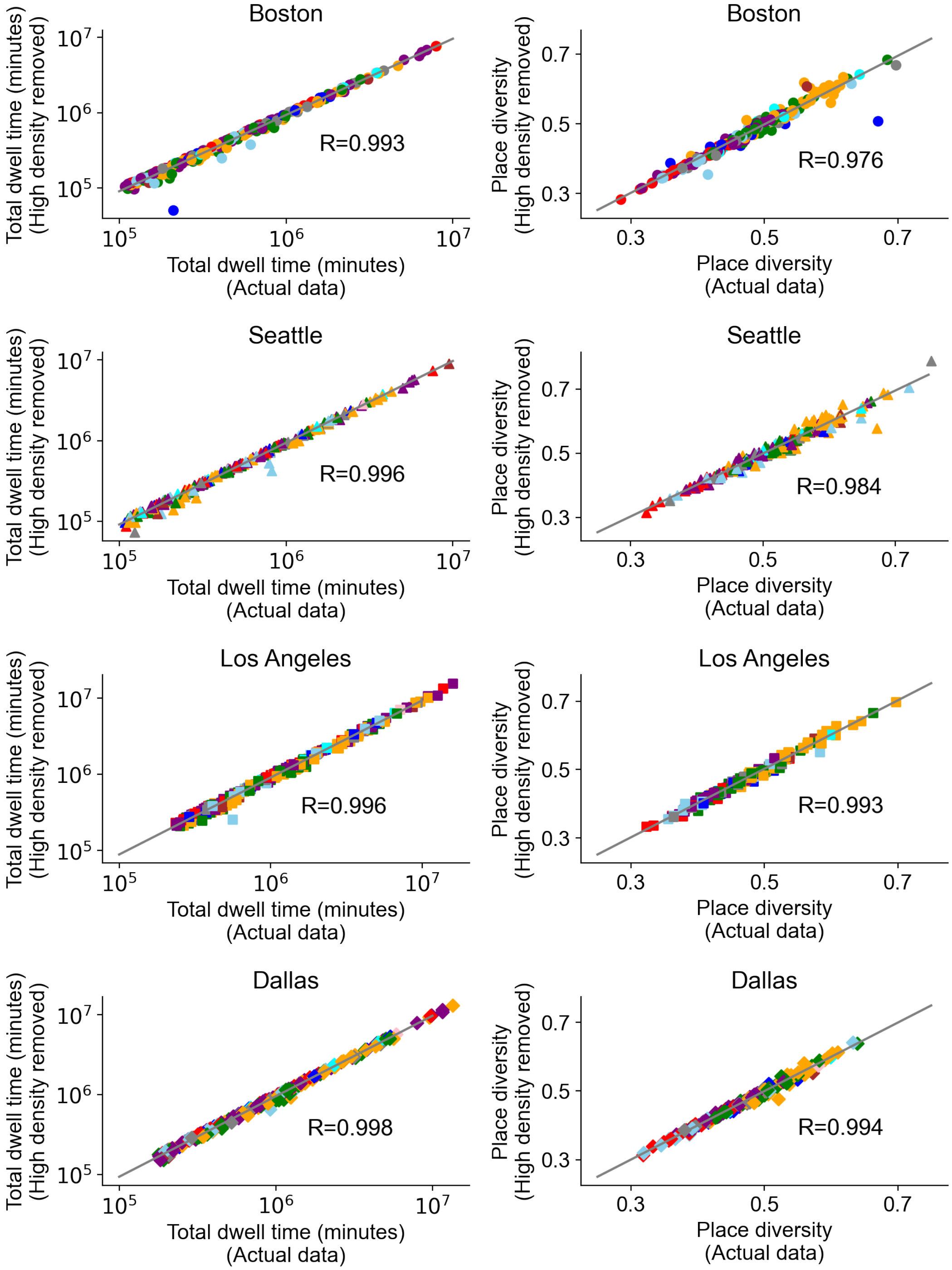

Figure S9: Total dwell time (left column) and place diversity (right column) of each place category are consistent when we remove high density POIs ($\geq$ 3 POIs within 20 meters) from the dataset. This result shows that the estimated dwell time and place diversity for each place category are robust to the inclusion of high density POIs.

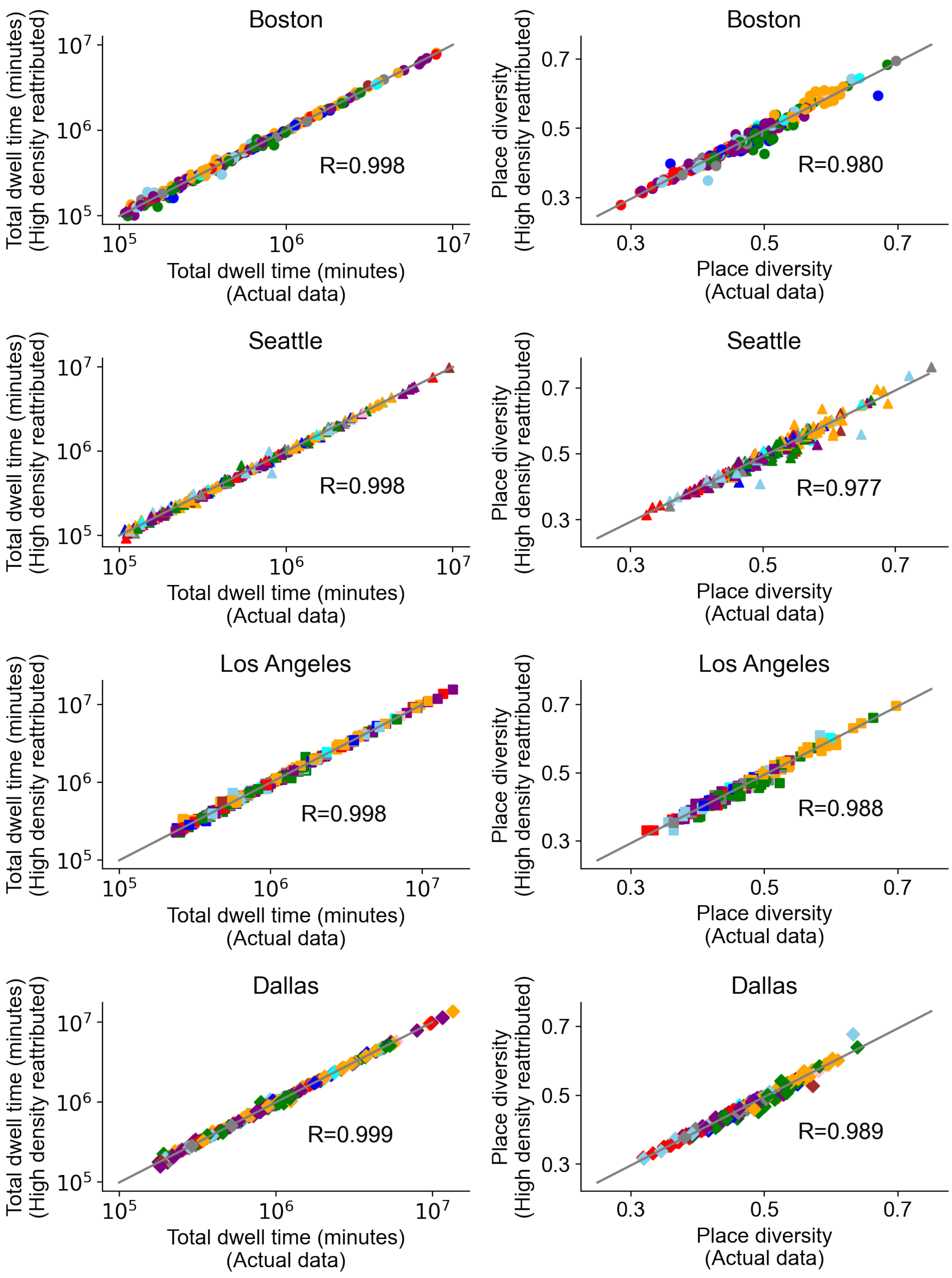

Figure S10: Total dwell time (left column) and place diversity (right column) of each place category are consistent when we refine the visit attribution to high density POIs ($\geq$ 3 POIs within 20 meters) using the Foursquare checkin dataset. This result shows that the estimated dwell time and place diversity for each place category are robust to the accuracy of visit attribution methods used for visits to high density POIs.

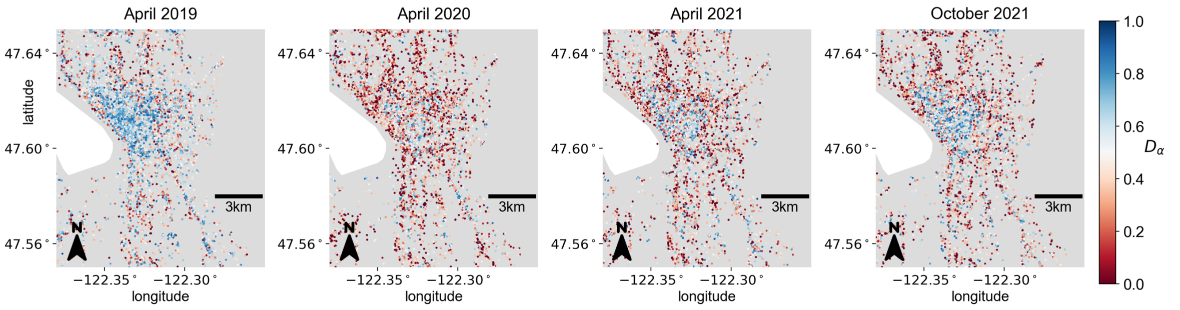

(a) Seattle

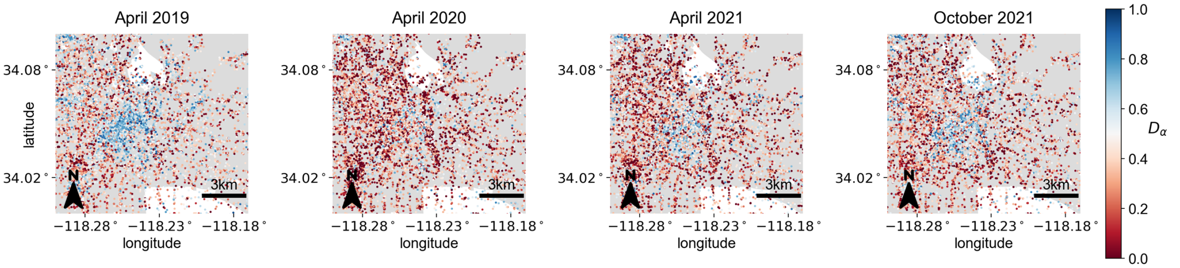

(b) Los Angeles

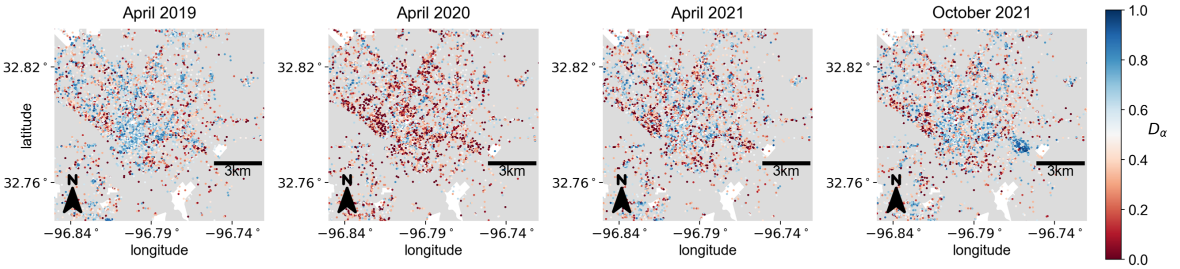

(c) Dallas

Figure S11: Income diversity of encounters in places in the three CBSAs, for (a) Seattle, (b) Los Angeles, and (c) Dallas (Boston is shown in main manuscript). Colors represent the income diversity level at each place. Maps were produced in Python using the TIGER shapefiles from the U.S. Census Bureau [28].

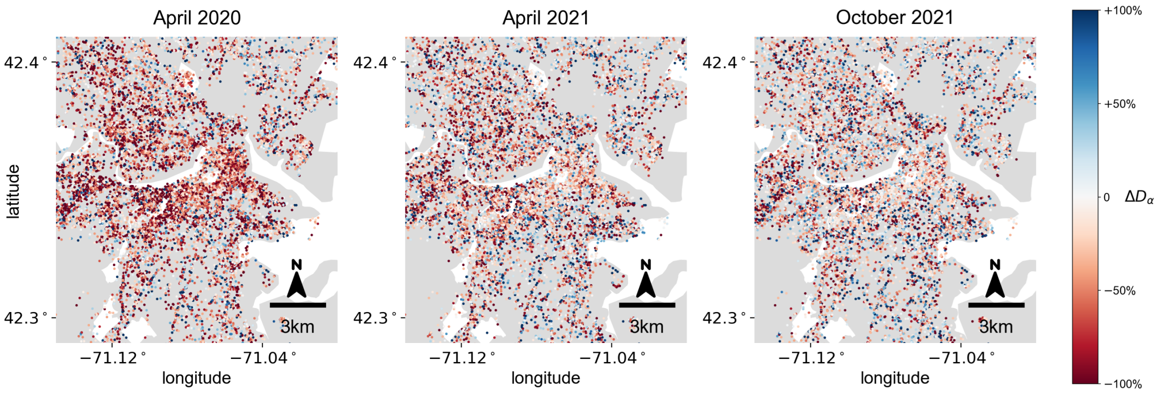

Figure S12: Percentage difference of income diversity at places in Boston, compared to same months in 2019. Insights are similar to Figure 1A the in main manuscript, where we see significant decrease in income diversity even in October 2021. Maps were produced in Python using the TIGER shapefiles from the U.S. Census Bureau [28].

The diversity measure is bounded between 0 and 1, where $D_\alpha = 0$ means there is no diversity (the place is visited by people from only one income quantile), and $D_\alpha = 1$ indicates that all income quantiles spent equal amount of time at the place. Results in Section 3.3 show that using different popular measures of diversity such as entropy does not affect the results on income diversity of encounters.

Figure S11 shows the changes in income diversity at places across four time periods: April 2019 (before the pandemic), April 2020, April 2021, and October 2021 in the four CBSAs. Figure S12 shows the percentage changes of income diversity at places in Boston, compared to same months in 2019. Figure S13 shows the income diversity experienced at different types of places across the four cities, across four time periods. Similar to the results for Boston in Figure 1D in the main manuscript, museums and leisure places had the largest decrease in diversity while health and grocery related places had the smallest decrease in diversity. This result agrees with the large decrease in visits to places such as museums, food places, and leisure places, as shown in Figure S14, indicating that the decrease in number of visits per user is correlated to the decrease in income diversity experienced at places. We further investigate how much of income diversity reduction is due to the decrease in the number of visits in Supplementary Note 4.

3.2 Income diversity experienced by individuals

In addition to the income diversity experienced at places, we are interested in measuring the income diversity that each individual experiences across all places they visit. Given the proportion of time individual i spent at place α, $\tau_{i\alpha}$, the individual's relative exposure to income quantile q, τ_{iq} can be computed by:

$$\tau_{iq} = \sum_{\alpha} \tau_{i\alpha} \tau_{q\alpha}. \quad (4)$$

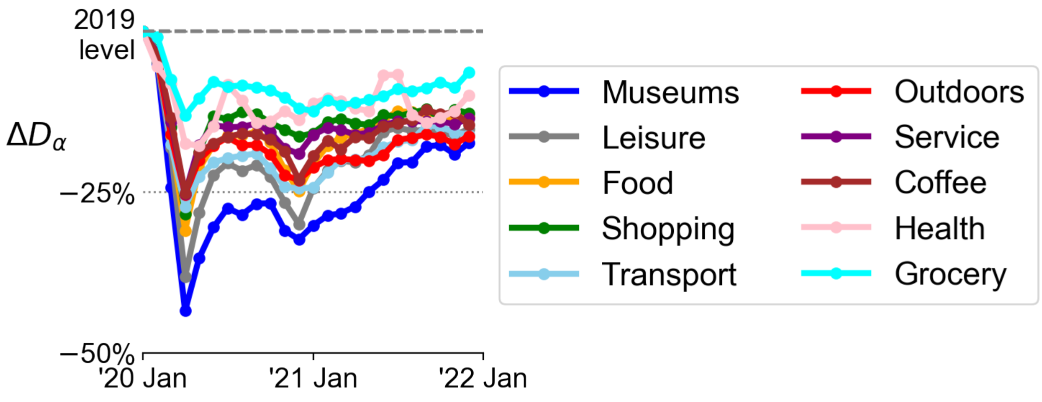

(a) Seattle

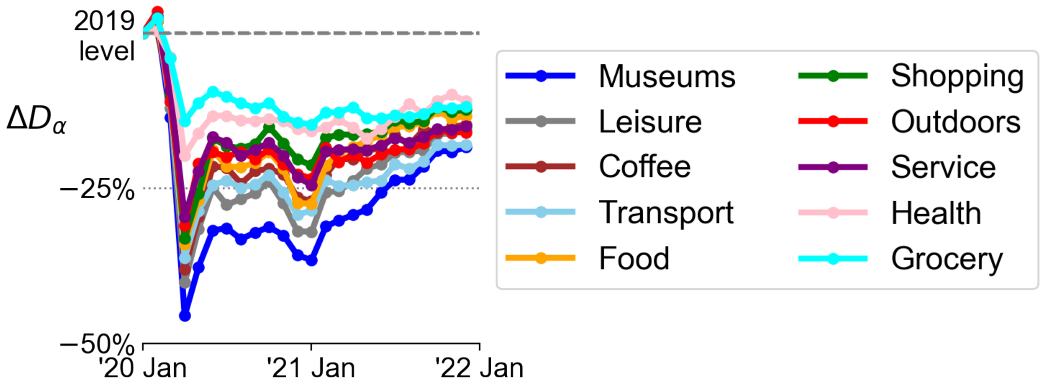

(b) Los Angeles

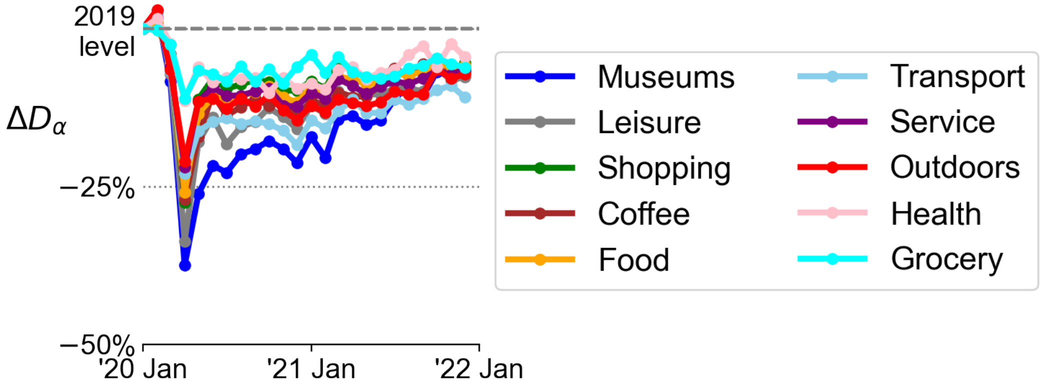

(c) Dallas

Figure S13: Average income diversity of encounters at different place categories in the three CBSAs, for (a) Seattle, (b) Los Angeles, and (c) Dallas (excluding Boston, which was in main manuscript). In all cities, museums, leisure, and transport places experienced the largest decrease in income diversity, while grocery and health places had the least decrease during the pandemic.

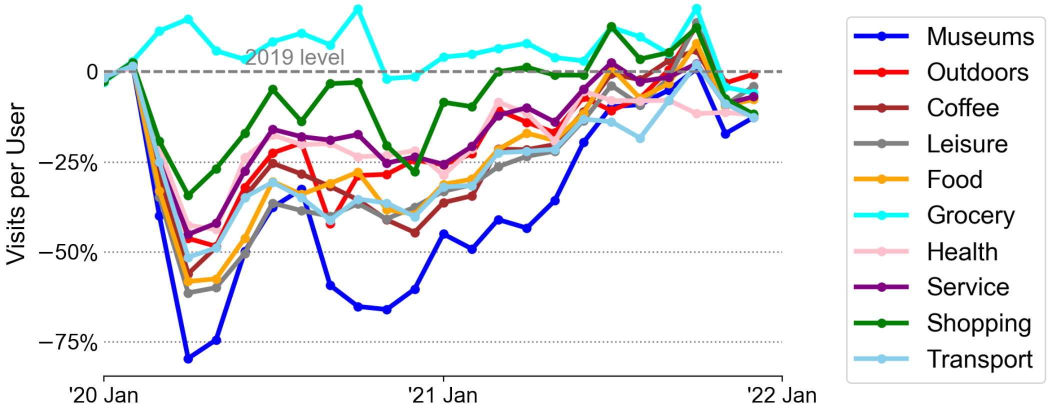

Figure S14: Time series data of the normalized visits per user to different place categories observed in each month, compared to pre-pandemic levels in 2019. Except for grocery places and shopping places, all place categories experience significant drops in visits during the pandemic, however, all recover back to pre-pandemic levels by late 2021.

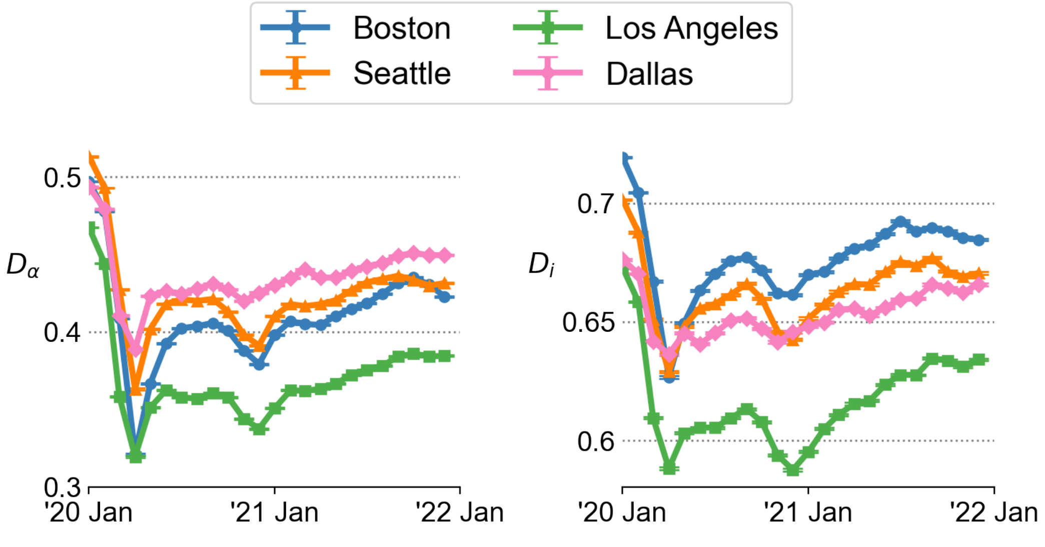

Figure S15: Absolute values of the income diversity of encounters experienced at places (left plot) and by individuals (right plot) across time for the four CBSAs. Places and individuals in Los Angeles has the lowest diversity, both pre-pandemic and during the pandemic. For interpretability, percentage differences are used in figures in the main manuscript.

Then, the income diversity experienced by individual i can be measured using the same equation used for places:

$$D_i = 1 - \frac{2}{3}\sum_q |\tau_{i\alpha} - \frac{1}{4}|. \quad (5)$$

Note that the exposure to income quantiles are calculated in a probabilistic manner across a two month time horizon to overcome the sparsity in actual encounters observed in the mobility data. Figure S15 shows the average income diversity at places and experienced by individuals for the four CBSAs. Los Angeles has the lowest income diversity both at places and by individuals out of the four cities. Different cities, which are located in different states, were restricted with COVID-19 lockdown polices of different levels of strictness. We investigate the regional differences from this perspective in Figure 4 in the main manuscript and in Section 6 in the Supplementary material. All monthly time series data, including the mean place diversity and individual diversity data are de-seasonalized by removing the monthly fluctuations (simply the deviations from the annual mean) observed in 2019. Most of the results in the main manuscript are shown by percentage differences, which is computed by $\Delta D_i(t) = \frac{D_i(t)-D_i(2019)}{D_i(2019)} \times 100(\%)$, where $D_i(2019)$ is the income diversity of encounters observed on the same month as t in 2019, before the pandemic.

3.3 Other measure of diversity: entropy

The metric for diversity used in our study captures the (un)evenness of exposure between different income quantile groups adopted in previous studies [20]. Another popular metric used to measure the (un)evenness of distribution groups is the entropy metric, which has been used in previous studies related to the diversity of communication networks across cities [6]. In our scenario, the entropy of the physical encounters at places are computed as the following:

$$H_\alpha = \frac{1}{\log 4}\sum_{q=1}^{4} \tau_{q\alpha} \log \tau_{q\alpha}. \quad (6)$$

The left panel in Figure S17 shows the histogram of the diversity (used in our study) and entropy of the encounters taken place at places. The histogram shows how the entropy metric is heavily skewed to high values between 0.8 and 1.0, whereas the diversity metric has relatively larger variability, spanning from 0 to 1. Despite these different characteristics, the right panel in Figure S17 plots the correlation between the diversity (x-axis) and entropy (y-axis) metrics. The Pearson's correlation between these two metrics is very high ($\rho = 0.971$), indicating that these two different metrics are both able to capture the income diversity of encounters.

Indeed, when using the entropy metric to measure the changes in diversity of encounters experienced at places and by individuals, we obtain similar results to when we use the diversity metric. Figure S18 shows how similar to Figure 1C in the main manuscript, we observe a decrease in income diversity of encounters during the first and second waves (April 2020 and December 2020). Moreover, the long-term decrease in diversity in late 2021 is consistent with the results using the diversity metric. Because of the consistency in the key insights between the two metrics, both these metrics are suitable for measuring the income diversity in encounters. Given the wider variability in the range between 0 and 1, we employ the diversity metric as our main metric for measuring income diversity.

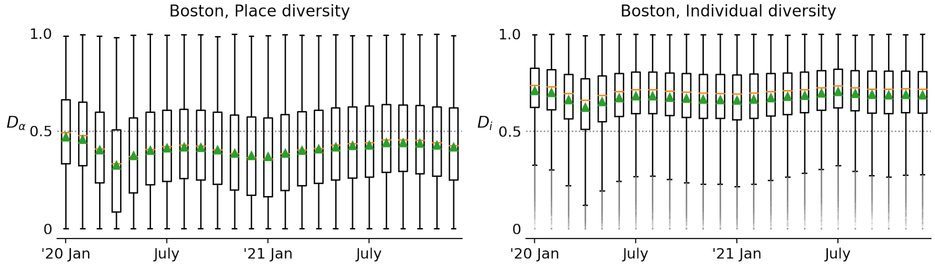

(a) Boston

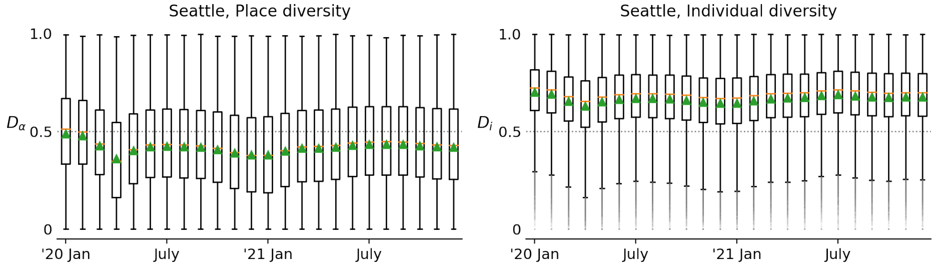

(b) Seattle

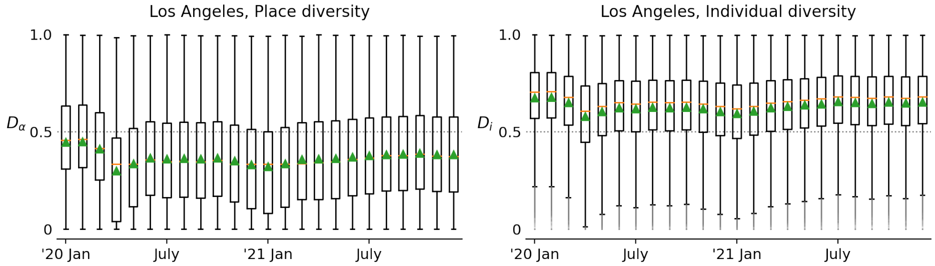

(c) Los Angeles

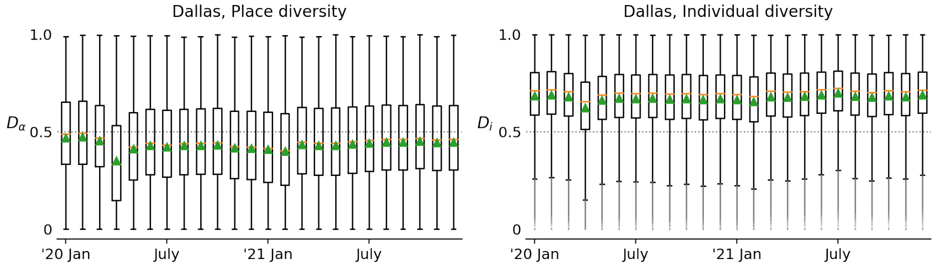

(d) Dallas

Figure S16: Box plots of place and individual income diversity across time in all four different cities, for (a) Boston, (b) Seattle, (c) Los Angeles, and (d) Dallas. The mean (green triangle) and the median (orange line) values are similar in all cities across all periods, suggesting that the selection of the aggregate metric (e.g., mean, median) does not substantially affect the results ($n = 71,989$ POIs for Boston, $n = 57,147$ POIs for Seattle, $n = 206,867$ POIs for Los Angeles, $n = 97,213$ POIs for Dallas). The box extends from the first quartile (Q1) to the third quartile (Q3) of the data, with a line at the median. The whiskers extend from the box by 1.5x the inter-quartile range (IQR). Flier points are those past the end of the whiskers.

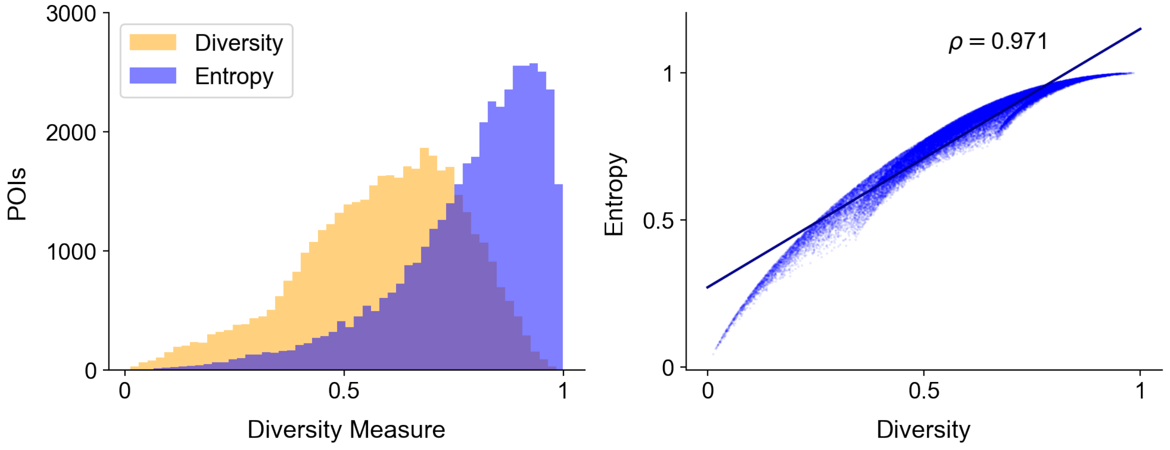

Figure S17: Comparison of the diversity and entropy metrics. The histogram shows how the entropy metric is heavily skewed to high values between 0.8 and 1.0, whereas the diversity metric has relatively larger variability, spanning from 0 to 1. The Pearson's correlation between these two metrics is very high ($\rho = 0.971$), indicating that these two different metrics are both able to capture the income diversity of encounters.

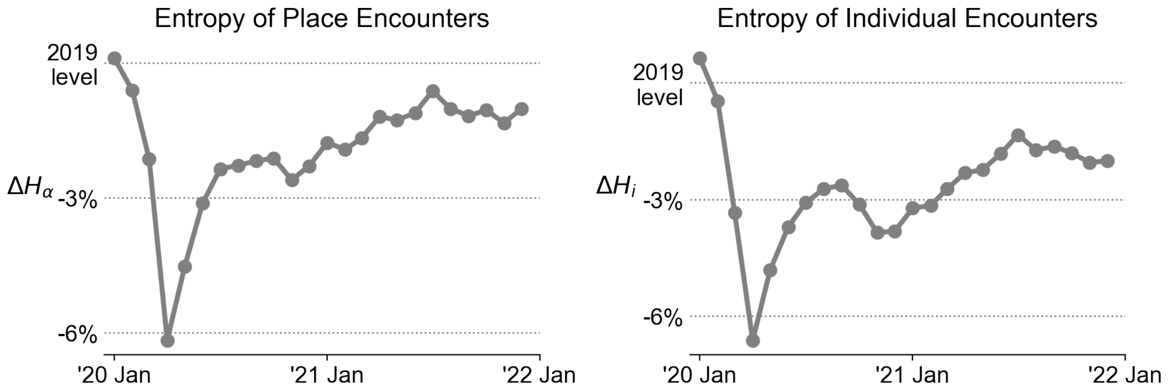

Figure S18: Income diversity of encounters measured using the entropy metric. Using entropy, we observe a decrease in income diversity of encounters during the first and second waves (April 2020 and December 2020). Moreover, the long-term decrease in diversity in late 2021 is consistent with the results using the diversity metric. Because of the consistency in the key insights between the two metrics, both these metrics are suitable for measuring the income diversity in encounters.

4 Counterfactual simulations

To understand the underlying behavioral changes that contributed to the decrease of income diversity in urban encounters, we design a simulation framework that leverages the pre-pandemic data to create synthetic, counterfactual mobility patterns. The synthetic, counterfactual mobility patterns dataset is designed so that while the fundamental behavioral patterns observed in 2019 are kept consistent, the number of visits to different place categories, in different distance ranges, by different income quantiles are reduced to post-pandemic levels. This way, we are able to delineate the effects of different levels of behavioral changes to the total decrease in income diversity. Each scenario was run 10 times, and the mean and standard errors of the estimated income diversity metrics were computed to ensure robust results.

4.1 Synthetic data generation procedure

The following steps are performed to simulate the synthetic mobility dataset. To create the synthetic counterfactual data for year y and month m, denoted as $\mathcal{S}_{y,m}$, we use the mobility data observed in the year 2019 on the same month m as input data $\mathcal{D}_{2019,m}$, for example, to create a synthetic mobility dataset for April 2020, we use the mobility data observed in April 2019. Three different synthetic data, $\mathcal{S}_{y,m}^{(i)}$, $\mathcal{S}_{y,m}^{(ii-1)}$, $\mathcal{S}_{y,m}^{(ii-2)}$, and $\mathcal{S}_{y,m}^{(ii-3)}$, are created based on different levels of detail. The steps for creating the synthetic datasets are as follows:

- $\mathcal{S}_{y,m}^{(i)}$: Randomly remove visits from $\mathcal{D}_{2019,m}$ to adjust the total amount of time spent at places outside home or workplaces to match $\mathcal{D}_{y,m}$
  - Visits are randomly retained by rate $r(y,m) = \min\left(1, \frac{\sum_{i\in\mathcal{D}_{y,m}} \tau_i}{\sum_{i\in\mathcal{D}_{2019,m}} \tau_i}\right)$, where $\sum_{i\in x} \tau_i$ is the total amount of dwell time duration spent by all users in dataset x. As a result, we obtain $\mathcal{S}_{y,m}^{(i)}$ which is a modified version of $\mathcal{D}_{2019,m}$ with adjusted total activity based on observations in the target year y and month m.
- $\mathcal{S}_{y,m}^{(ii-1)}$: Randomly remove visits from $\mathcal{D}_{2019,m}$ by income quantiles q to adjust the total dwell time at places
  - Visits are randomly retained by rate $r(y,m,q) = \min\left(1, \frac{\sum_{i\in\mathcal{D}_{y,m}(q)} \tau_i}{\sum_{i\in\mathcal{D}_{2019,m}(q)} \tau_i}\right)$, where $\sum_{i\in x(q)} \tau_i$ is the total amount of dwell time spent by all users in dataset x by users from income quantile q. As a result, we obtain $\mathcal{S}_{y,m}^{(ii-1)}$ which is a modified dataset of $\mathcal{D}_{2019,m}$ with adjusted number of visits based on observations in the target year y and month m.
- $\mathcal{S}_{y,m}^{(ii-2)}$: Randomly remove visits from $\mathcal{D}_{2019,m}$ by income quantiles q and traveled distance d to adjust the total dwell time at places
  - Visits are randomly retained by rate $r(y,m,q,d) = \min\left(1, \frac{\sum_{i\in\mathcal{D}_{y,m}(q,d)} \tau_i}{\sum_{i\in\mathcal{D}_{2019,m}(q,d)} \tau_i}\right)$, where $\sum_{i\in x(q,d)} \tau_i$ is the total amount of dwell time spent by all users in dataset x by users from income quantile q within distance d from the user's home location. d was binned into 7 distance ranges: $[0km, 1km)$, $[1km, 3km)$, $[3km, 5km)$, $[5km, 10km)$, $[10km, 20km)$, $[20km, 40km)$, $[40km, \infty]$ to obtain rates for each category. As a result, we obtain $\mathcal{S}_{y,m}^{(ii-2)}$

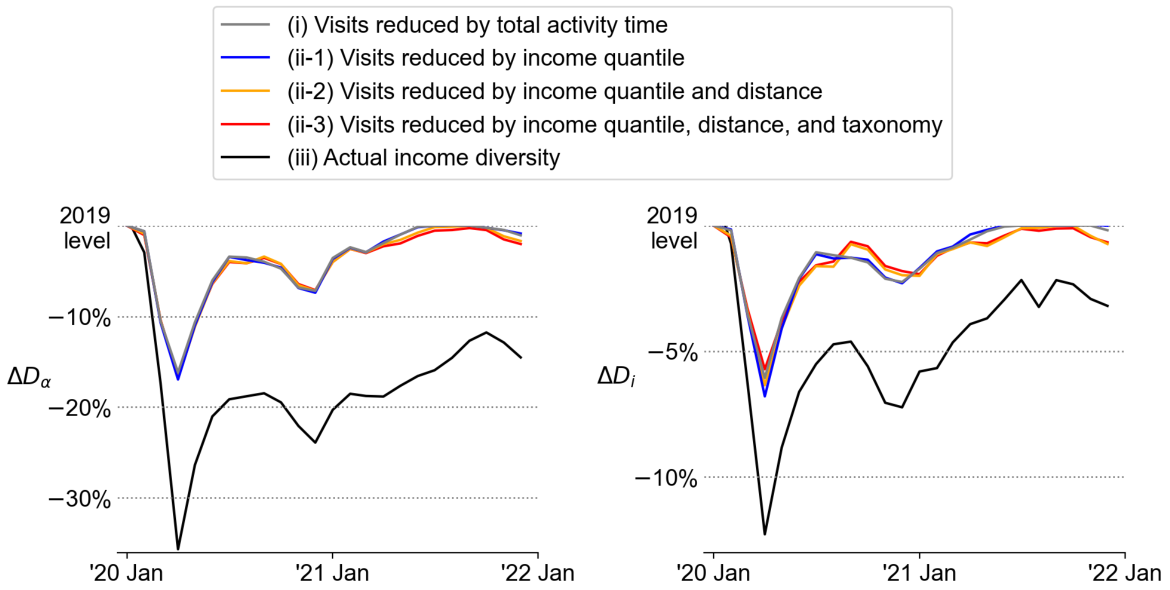

Figure S19: Comparison of counterfactual scenarios for Boston where the visits are reduced based on (i) total activity time, (ii-1) activity time categorized by income quantiles, (ii-2) activity time categorized by income quantiles and distance distributions, and (ii-3) activity time categorized by income quantiles, distance distributions, and POI taxonomy, and (iii) actual income diversity. Scenarios (i) and (ii-2) was employed in the main manuscript since there was little difference between scenarios (i) and (ii-1), and (ii-2) and (ii-3), respectively.

which is a modified dataset of $\mathcal{D}_{2019,m}$ with adjusted number of visits based on observations in the target year y and month m.

- $\mathcal{S}_{y,m}^{(ii-3)}$: Randomly remove visits from $\mathcal{D}_{2019,m}$ by income quantiles q, place taxonomy c, and traveled distance d to adjust the total dwell time spent at places

  – Visits are randomly retained by rate $r(y,m,q,d,c) = \min\left(1, \frac{\sum_{i \in \mathcal{D}_{y,m}(q,d,c)} \tau_i}{\sum_{i \in \mathcal{D}_{2019,m}(q,d,c)} \tau_i}\right)$, where $\sum_{i \in x(q,d,c)} \tau_i$ is the total amount of dwell time spent by all users in dataset x by users from income quantile q, to places in major taxonomy c, within distance d from the user's home location. Similar to the previous counterfactual, d was binned into the same 7 distance ranges to obtain rates for each category. The 10 taxonomies shown in Table S1 are used. As a result, we obtain $\mathcal{S}_{y,m}^{(iii-3)}$ which is a modified version of $\mathcal{D}_{2019,m}$ with adjusted number of visits based on observations in the target year y and month m.

After creating the synthetic counterfactual datasets $\mathcal{S}_{y,m}^{(i)}$, $\mathcal{S}_{y,m}^{(ii-1)}$, $\mathcal{S}_{y,m}^{(ii-2)}$, and $\mathcal{S}_{y,m}^{(ii-3)}$ from the observed changes in aggregate behavior metrics, we compute the income diversity of encounters and compare with the income diversity measured using the actual observed data $\mathcal{D}_{y,m}$. Figure S19 shows the percentage changes in income diversity at places ΔD_α and by individuals ΔD_i computed using the different counterfactual datasets. The results indicate that the counterfactual scenarios using $\mathcal{S}_{y,m}^{(i)}$, $\mathcal{S}_{y,m}^{(ii-1)}$, $\mathcal{S}_{y,m}^{(ii-2)}$, and $\mathcal{S}_{y,m}^{(ii-3)}$ yield similar results. In particular, results from $\mathcal{S}_{y,m}^{(i)}$ and $\mathcal{S}_{y,m}^{(ii-1)}$, and

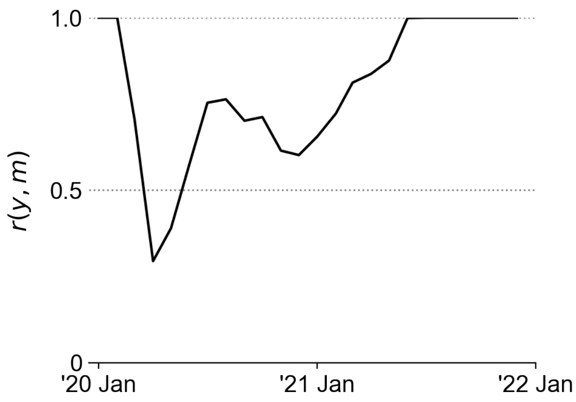

(a) Retain rate using total dwell time.

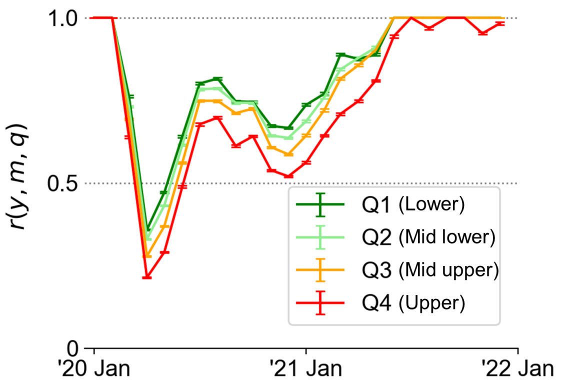

(b) Retain rate by income quantiles.

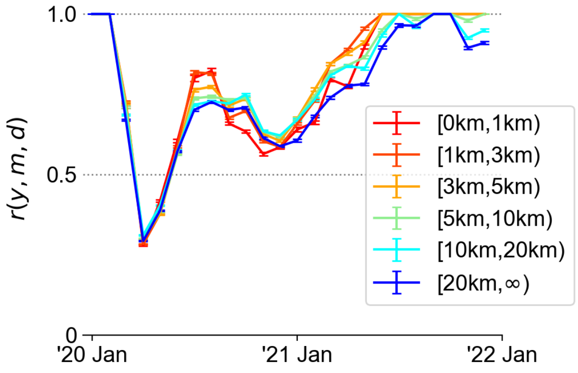

(c) Retain rate by travel distance.

Figure S20: Retain rates used to generate mobility datasets under different counterfactual scenarios. (a) retain rate using total dwell time is used for creating the counterfactual dataset $\mathcal{S}^{(i)}_{y,m}$, (b) retain rate by income quantiles and (c) retain rate by travel distance are used to create the counterfactual dataset $\mathcal{S}^{(ii)}_{y,m}$.

$\mathcal{S}_{y,m}^{(ii-2)}$ and $\mathcal{S}_{y,m}^{(ii-3)}$, generate similar patterns, indicating that the effects of controlling by income quantiles and place taxonomies are negligible.

To summarize the findings, counterfactual simulations show that:

1. Using different retain rates across income quantiles have no effect on income diversity measures (no difference between $\mathcal{S}_{y,m}^{(i)}$ and $\mathcal{S}_{y,m}^{(ii-1)}$);
2. Using different retain rates across distance distributions have slight effects on income diversity measures (slight difference between $\mathcal{S}_{y,m}^{(ii-1)}$ and $\mathcal{S}_{y,m}^{(ii-2)}$), and;
3. Using different retain rates across place taxonomies (major categories) have no effect on income diversity measures (no difference between $\mathcal{S}_{y,m}^{(ii-2)}$ and $\mathcal{S}_{y,m}^{(ii-3)}$),

which will be further investigated in the following sections.

4.2 Analysis of the impacts of removal rates under different scenarios

1. Effects of different retain rates across income quantiles

To understand why the impacts of using different retain rates across income quantiles (as shown in Figure S20b) yield no difference in diversity decrease, we plot the histograms of τ_α, q of each place α for each income quantile q and in aggregate, in Figures S21a and S21b, respectively, for the two counterfactual scenarios (i) and (ii-1). We observe that, in agreement with Figure S20b, during the pandemic τ_{q_1} and τ_{q_2} increased and τ_{q_4} decreased due to lower income populations disproportionately visiting places than higher income people. However, when we aggregate and plot the τ_q values for all $q \in \{q_1, q_2, q_3, q_4\}$, there is no significant difference across the two counterfactual scenarios, consequentially yielding similar values of diversity, since the diversity measure does not differentiate whether q_1 or q_4 had disproportionate dwell time spent at places.

2. Effects of different retain rates across distance distributions

The retain rates across distance ranges shown in Figure S20c show that during most of the periods in the pandemic, shorter distance trips (e.g., $[0, 1km)$, $[1km, 3km)$) have higher retain rates compared to longer distance trips (e.g., $[20km, \infty)$), indicating that people preferred shorter distance trips than longer ones during the pandemic. As shown in previous studies, longer distance trips tend to result in higher diversity, whereas shorter distance trips are less diverse due to stronger effects of residential segregation [20]. Indeed, when we compare results (ii-1) and (ii-2) in Figure S19, especially ΔD_i, scenario (ii-2) has lower diversity during periods when $r(y, m, d)$ for shorter distances are higher than longer distances (i.e., June – September 2020, January 2021 – June 2021). On the other hand, scenario (ii-2) has higher diversity during periods when $r(y, m, d)$ for shorter distances are lower than longer distances (i.e., September – December 2020). These observations show that changes in distance distributions does play a role in the income diversity of urban encounters, despite the small magnitude of the effects as shown in Figure S19.

3. Effects of different retain rates across place taxonomies (major categories)

The retain rates across place taxonomies (major categories) shown in Figure S22a indicate that different major categories had varying rates during the pandemic. While most categories follow similar

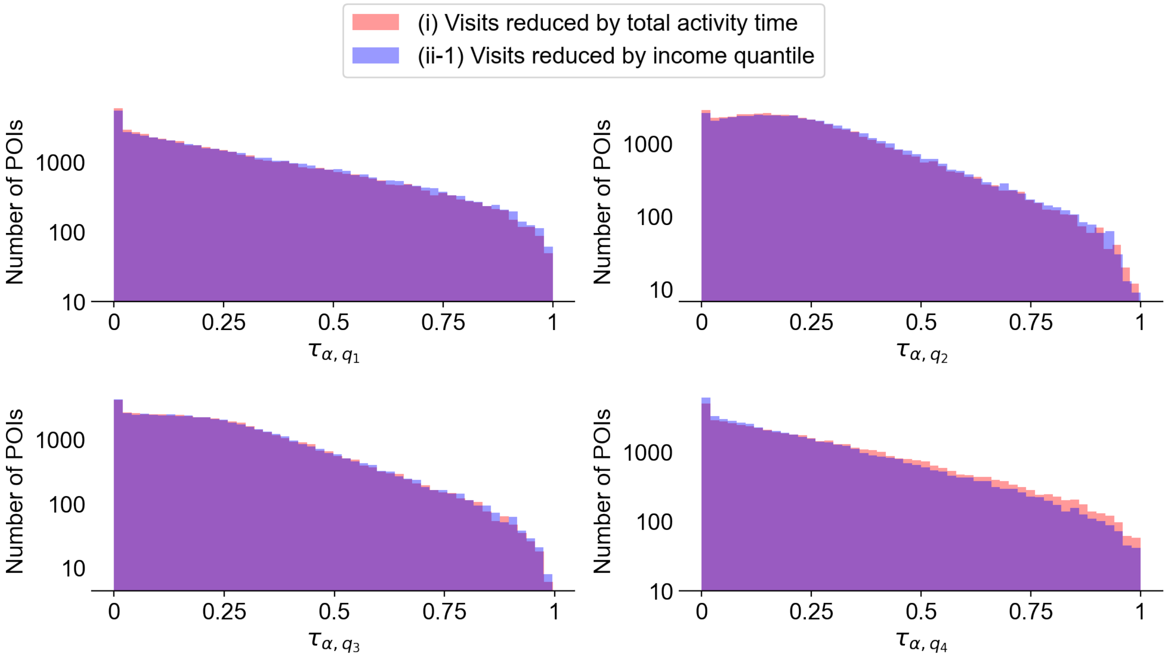

(a) Histograms of τ_q for q_1,q_2,q_3,q_4, respectively, for counterfactual scenarios (i) and (ii-1).

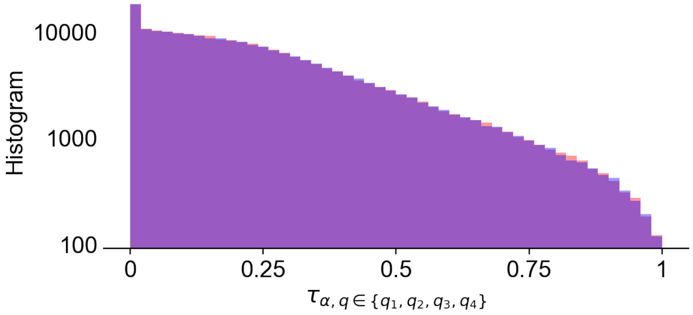

(b) Histograms of $\tau_{q \in \{q_1,q_2,q_3,q_4\}}$ for counterfactual scenarios (i) and (ii-1).

Figure S21: (a) Histograms of $\tau_{q \in \{q_1,q_2,q_3,q_4\}}$ for counterfactual scenarios (i) and (ii-1). (b) Differences in the distributions of τ_q between counterfactual scenarios (i) and (ii-1) are significant for each income quantile, but are nearly identical when aggregated across all income quantiles, yielding similar income diversity measures.

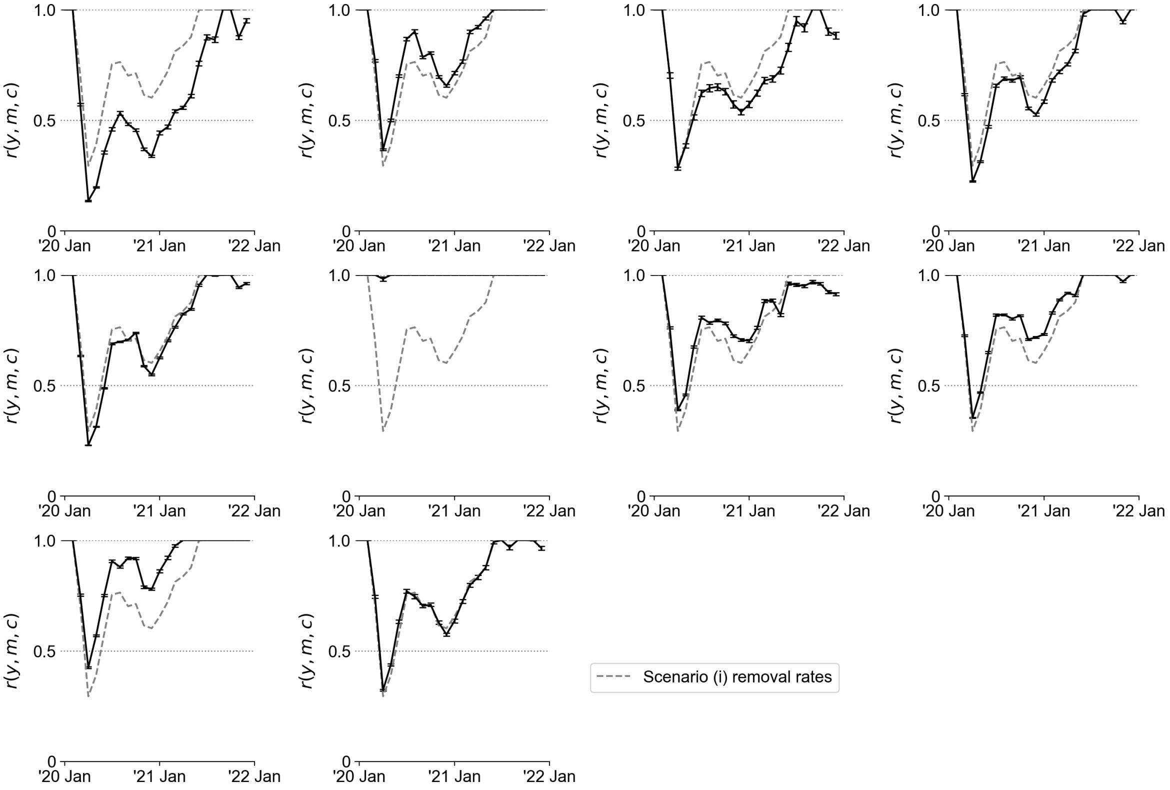

(a) Retain rates for different place taxonomies (major categories).

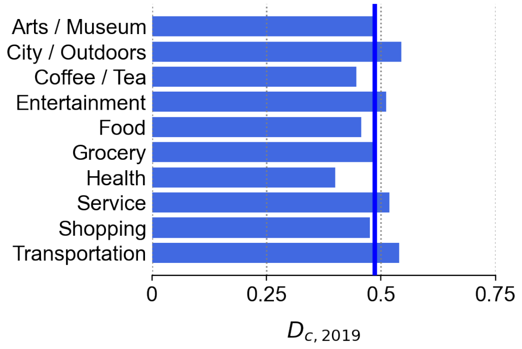

(b) Average baseline diversity metric for each place taxonomy.

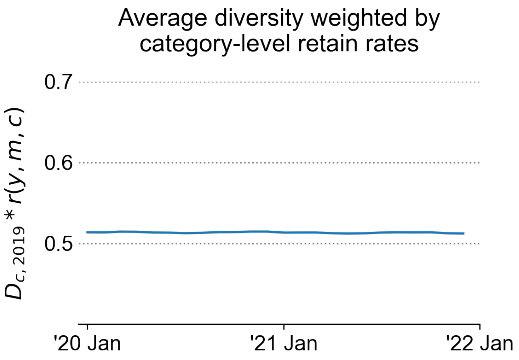

(c) Average diversity weighted by category-level retain rates.

Figure S22: (a) Heterogeneous retain rates across place taxonomies (major categories) suggest income diversity measures to be affected by adding place taxonomies as a constraint for creating counterfactual datasets in (ii-3). (b) However, the effects are close to zero, since place categories which have substantially different retain rates (i.e., grocery and arts/museums) have average level diversity measures.

patterns as the overall average retain rates shown in Figure S20a, places such as grocery stores had significantly higher (almost full) retain rates, indicating that dwell times at grocery stores had very small decreases. On the other hand, arts and museums had the largest decrease in retain rates. These heterogeneous rates suggest that using different retain rates across place categories when producing the counterfactual dataset (ii-3) could significantly affect the income diversity of $\mathcal{S}_{y,m}^{(ii-3)}$.

However, as shown in Figure S19, this additional constraint of controlling by place taxonomies yield negligible effects. We test this by computing the average diversity weighted by category-level retain rates across time. More specifically, we take the 2019 level diversity of each place taxonomy, $D_{c,2019}$, and re-weight them by the time-varying retain rates $r(y, m, c)$. The results in Figure S22c show almost a flat trend across time, indicating that the heterogeneity in the time-varying retain rates have no effects on the overall income diversity. This can be explained by looking at place taxonomies that had the largest deviations in retain rates – grocery stores and arts/museum places had close to the average diversity measures, as shown in Figure S22b.

4.3 Summary of counterfactual simulation results

Since the effects of heterogeneous retain rates across place taxonomies was insignificant, results for counterfactual diversity decrease using the $\mathcal{S}_{y,m}^{(ii-1)}$ and $\mathcal{S}_{y,m}^{(ii-3)}$ datasets were omitted from Figure 2B in the main manuscript. Figure S19 shows the comparison of counterfactual scenarios for Boston where the visits are reduced based on (i) total activity time ($\mathcal{S}_{y,m}^{(i)}$), (ii-1) activity time categorized by distance and income quantile ($\mathcal{S}_{y,m}^{(ii)}$), and (ii-2) activity time categorized by distance, income quantile, and POI taxonomy ($\mathcal{S}_{y,m}^{(iii)}$), and (iii) actual income diversity. Scenario (ii-1) was employed in the main manuscript since there was little difference between scenarios (ii-1) and (ii-2). For all cities, the decrease in income diversity when we consider the reduction in users and visits by quantile accounts ($\mathcal{S}_{y,m}^{(i)}$) for around 50% of the reduction in diversity in the initial stages of the pandemic in the early stages of the pandemic. The marginal decrease in the diversity due to the reduction in visits based on place categories and travel distances ($\mathcal{S}_{y,m}^{(ii)}$) is relatively small compared to the reduction in visits.

However, as shown in Figures S19, S23, and S24 (which shows the proportion of the three factors), these reductions in active users and visits to different categories do not account for all of the reduction in income diversity, and indicates that more microscopic changes in human behavior have contributed to a further decrease in income diversity in cities during the pandemic. To investigate what behavioral changes during the pandemic contributed to the decrease in income diversity, we seek to find any microscopic, individual level behavior that changed during the later stages of the pandemic. To do that, we analyze the behavioral parameters of the Social-EPR model (proposed in [20], which extended the EPR model proposed in [25]).

4.4 Parameters of the Social-EPR model

The social exploration and preferential return (Social-EPR) model [20, 25] characterizes visitation patterns of individuals using two mechanisms: exploration (visiting a new place) or preferential return (visiting an already visited place). The probability of exploration when an individual has already visited S_T places is modeled as $P_{new} = \rho S_T^{-\gamma}$, where ρ and γ are model parameters. If an individual decides to explore, they then decide whether to socially explore (visit a new place where their income group is not the majority income quantile group) with probability σ_s. In the case that the individual decides to return, the individual selects the destination α with probability $\Pi_\alpha \sim \tau_{\alpha,i}$, where $\tau_{\alpha,i}$ is the

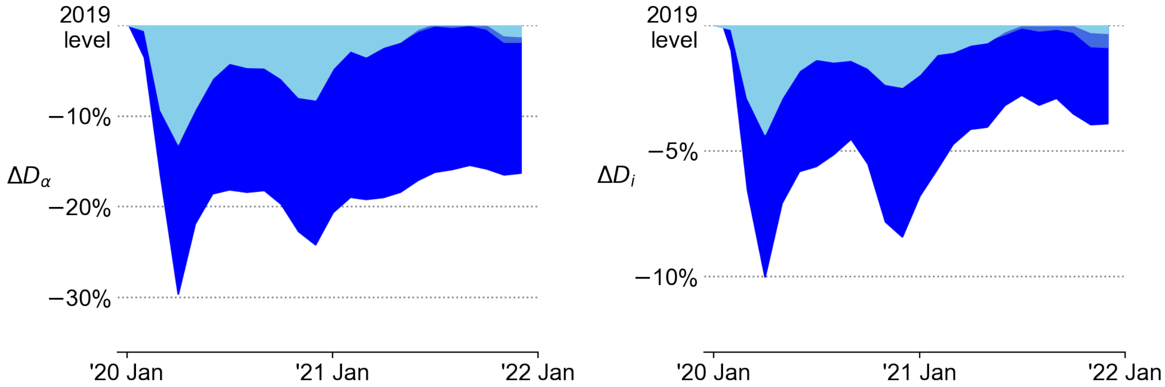

(a) Seattle

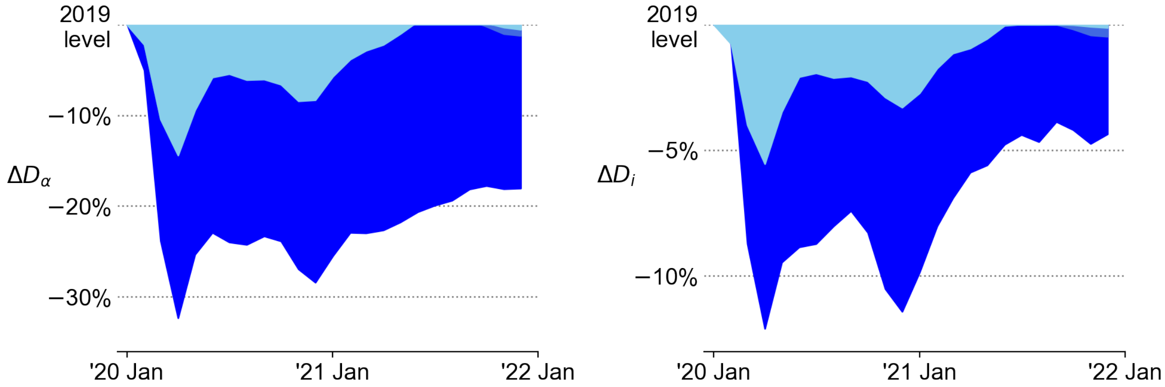

(b) Los Angeles

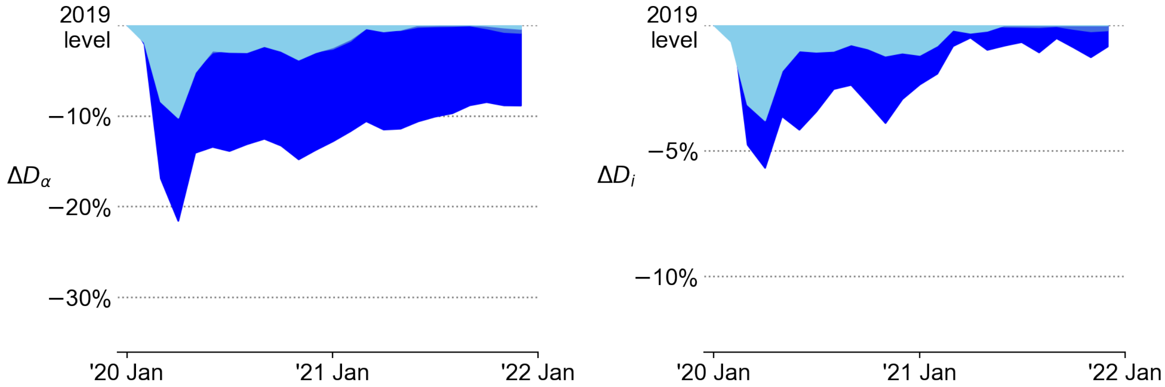

(c) Dallas

Figure S23: Percentage changes in income diversity of encounters in places and by individuals in the three CBSAs under different synthetic counterfactual scenarios, for (a) Seattle, (b) Los Angeles, and (c) Dallas (Boston is shown in Figure 2B in main manuscript). Similar to Figure 2B in the main manuscript, most of the decrease in income diversity can be attributed to microscopic changes in mobility behavior, especially during the later stages of the pandemic.

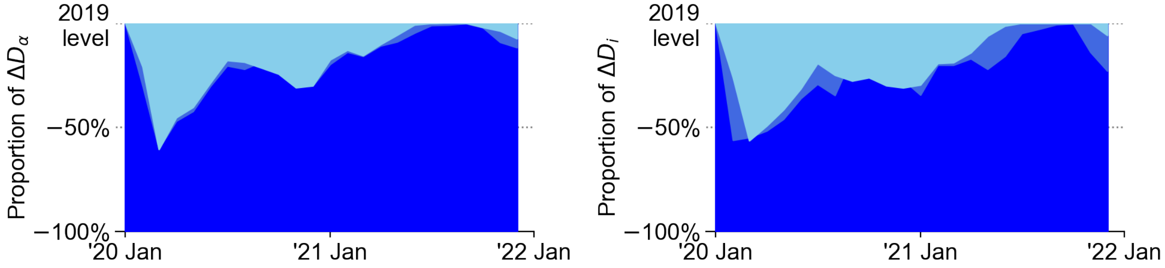

(a) Boston

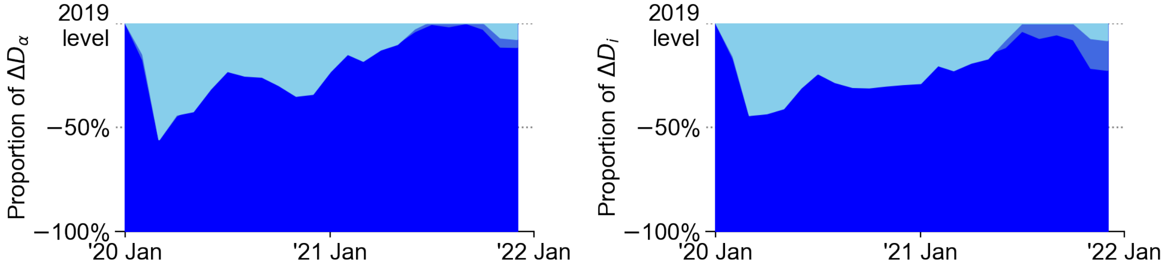

(b) Seattle

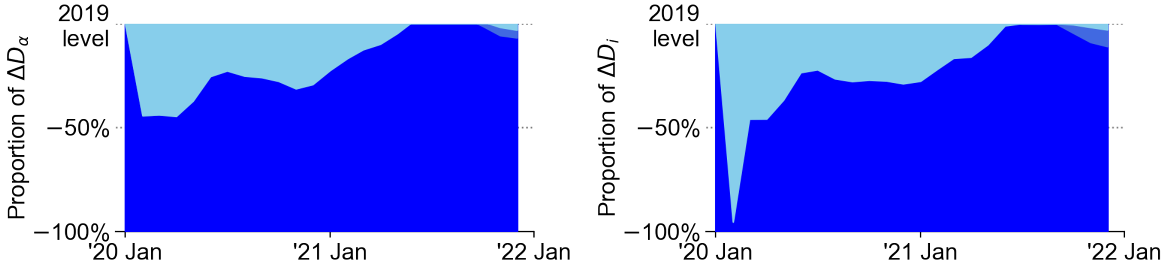

(c) Los Angeles

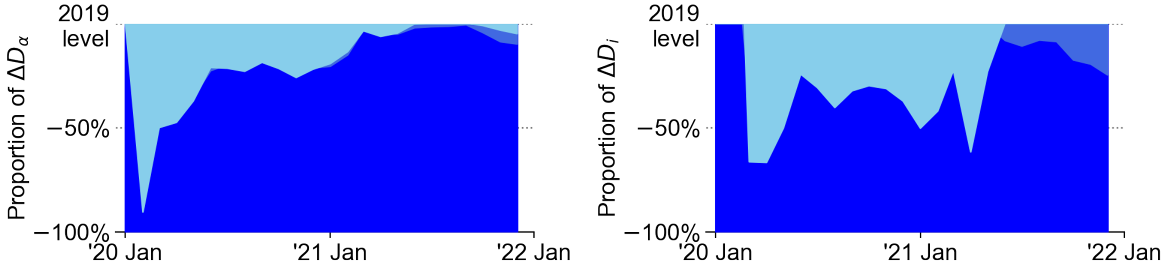

(d) Dallas

Figure S24: Proportion of percentage changes in income diversity of encounters in places and by individuals in the four CBSAs under different synthetic counterfactual scenarios, for (a) Boston, (b) Seattle, (c) Los Angeles, and (d) Dallas. Showing the proportions highlight the insights from the previous figure, where most of the decrease in income diversity can be attributed to microscopic changes in mobility behavior, especially during the later stages of the pandemic.

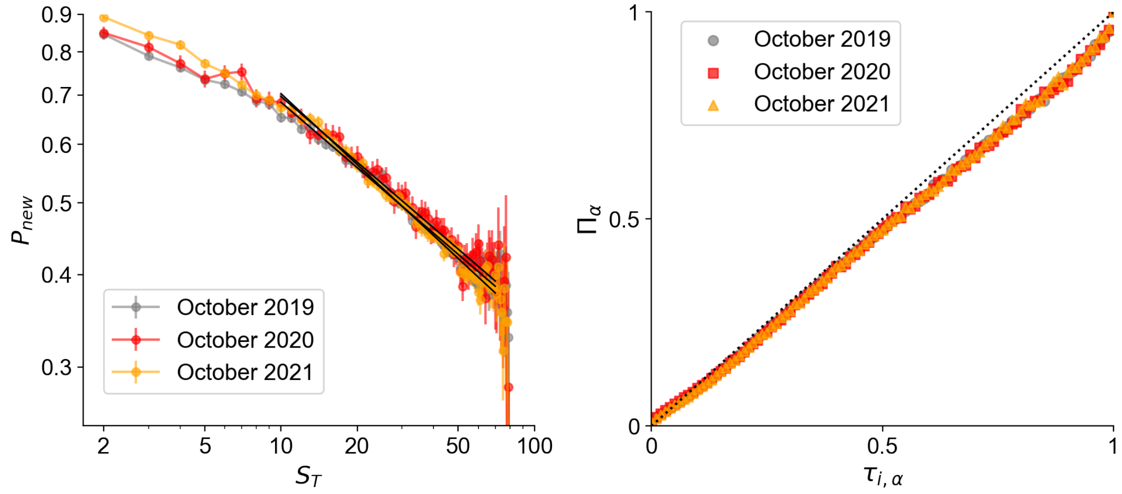

(a) Place exploration parameters

(b) Preferential return parameters

Figure S25: Key parameters of the Social-EPR model, ρ and γ (shown in panel a), and π (shown in panel b), are fairly consistent during the pandemic. The social exploration parameter σ_s (shown in main manuscript Figure 2D) was the only parameter with significant changes. Data are presented as mean values $+$ / $-$ SEM.

proportion of time already spent at place α by individual i.

To investigate whether any of the fundamental behavioral characteristics have changed due to the pandemic, we fitted the Social-EPR model to the observed mobility data patterns and estimated the model parameters across different periods of time. The fitted parameters are shown in Figure S25. Surprisingly, we find that the key parameters of the Social-EPR model, including ρ, γ, and linear relationship between Π_α and $\tau_{\alpha,i}$, are mostly consistent across time (with the exception of April and May 2020 due to the initial lockdown). This indicates that the fundamental characteristics of individual mobility, including exploration and preferential return, were consistent during the pandemic, when controlled by the number of visits an individual makes. The model parameter with the most significant change during the pandemic was the social exploration parameter σ_s, as shown in Figure 2D in the main manuscript, and in Figure S26, which shows the decrease in social exploration during the pandemic, even when compared to the counterfactual scenario (ii).

From the counterfactual experiment, we found that there is an excess level of decrease in diversity in urban encounters even when controlled for the number of visits to different place categories by different income groups, by travelled distance. The Social-EPR model revealed that such decrease was not due to changes in exploration and preferential return behavior, but because of decrease in social exploration behavior and microscopic changes in where people prefer to visit (sub-category level changes), which is shown in Figure S27. Across all four CBSAs, we observe that places such as hardware, big box stores, banks, and grocery stores were the places with the highest increase in the proportion of individuals who visited them with a top-10 frequency, while gyms, food places (pizza, fast food), apparel, movie theaters were places with the largest decrease.

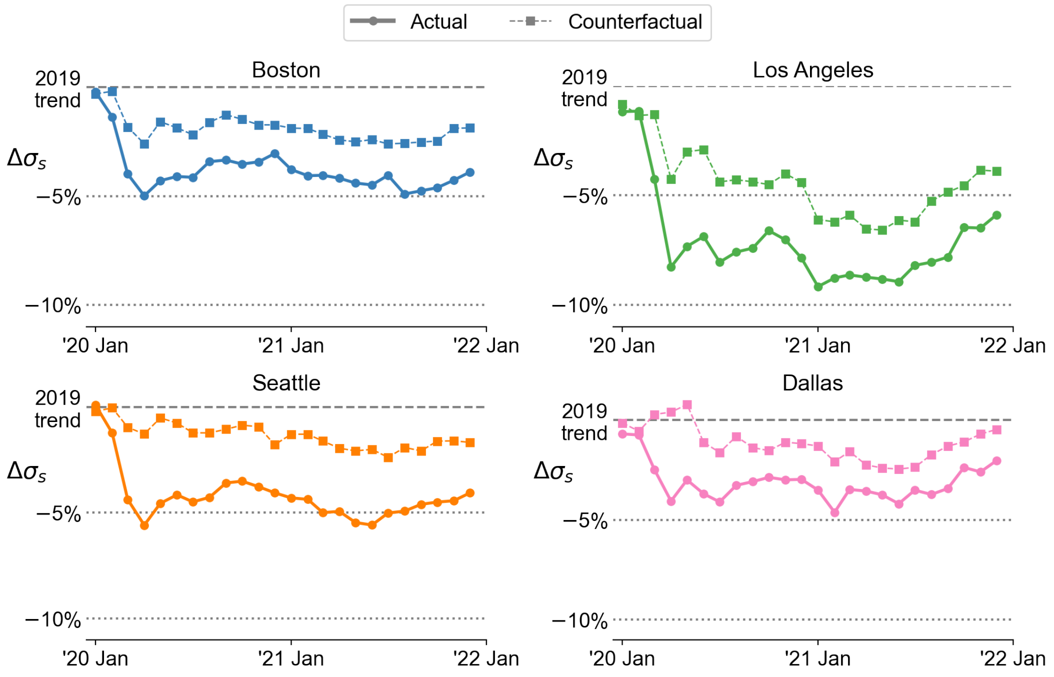

Figure S26: The model parameter with the most significant change during the pandemic was the social exploration parameter σ_s, even when compared with the counterfactual simulation results.

5 Explaining spatial heterogeneity in diversity

5.1 Regression models and results

To further understand how the income diversity of encounters decreased heterogeneously across sociodemographic groups during throughout the pandemic, we build simple linear regression models of the form:

$$D_{CBG}(t), \Delta D_{CBG}(t) \sim \{R_{CBG}\} + \{P_{CBG}\} + \{M_{CBG}\} \quad (7)$$

where $D_{CBG}(t)$ and $\Delta D_{CBG}(t)$ denote the differences in diversity at time t compared to the same month in the year 2019, and:

- $\{R_{CBG}\}$ is the set of all residential variables from the census that describe the demographic, transportation, education, race, employment, wealth, etc. of the Census Block Group. The entire list of these variables can be found in Table S3.

- $\{P_{CBG}\}$ is a vector of variables that indicate the places where individuals living in the CBG spent most of their time in 2019, out of the place subcategories which have at least 100 venues. For each individual, we identify the subcategories where the individual stays more than 0.3% of their time and obtain a binary vector with the length of 564, which is the number of place subcategories. Then to obtain $\{P_{CBG}\}$ we simply take the average of the vectors of all individuals who are living in the corresponding CBG. The threshold method previously employed in [20] are used for sparse and highly-skewed human data [5] to minimize the effect of the noisy and long-tailed distribution of human activities.

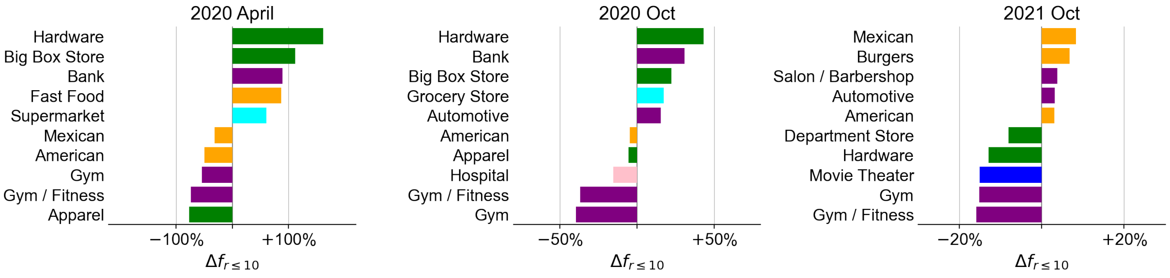

(a) Seattle

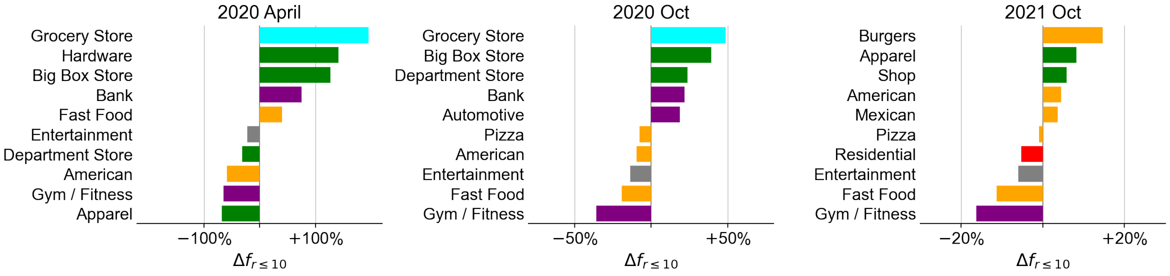

(b) Los Angeles

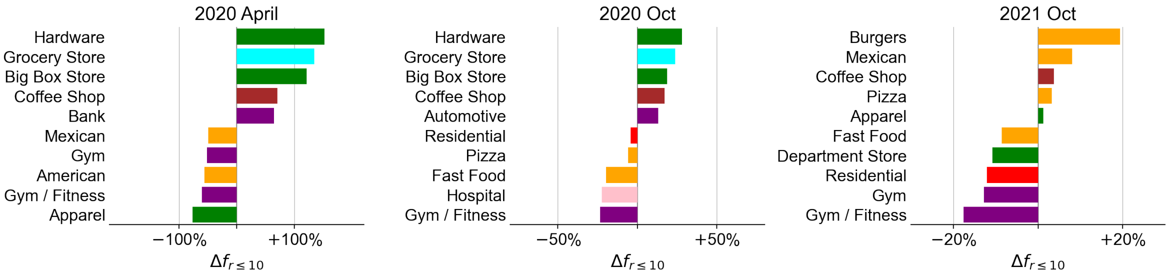

(c) Dallas

Figure S27: Changes in proportion of high-frequency visitation to place subcategories across different periods of the pandemic in (a) Seattle, (b) Los Angeles, and (c) Dallas (Boston is shown in main manuscript).

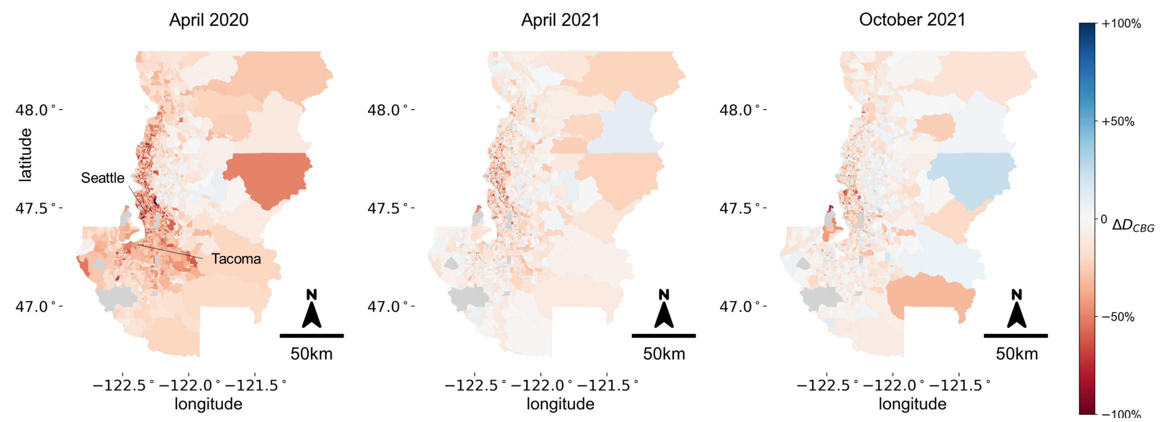

(a) Seattle

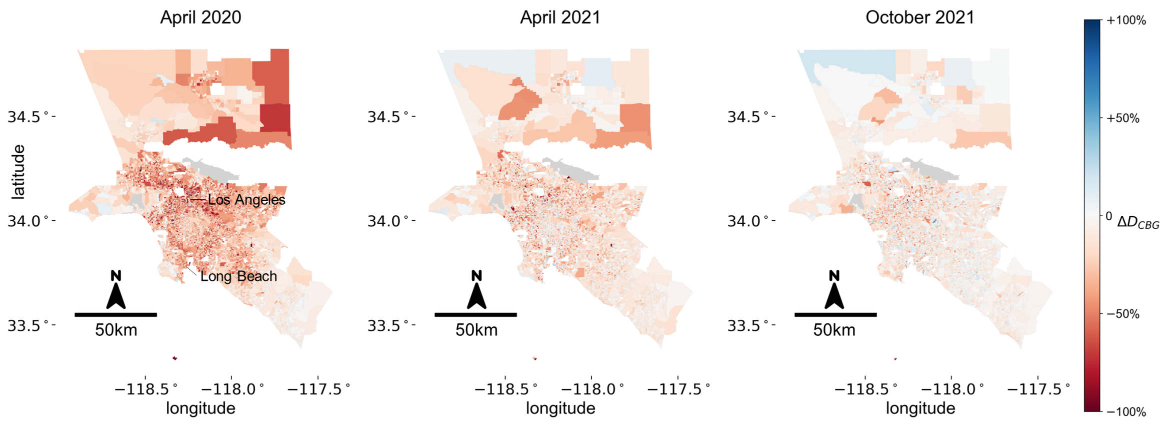

(b) Los Angeles

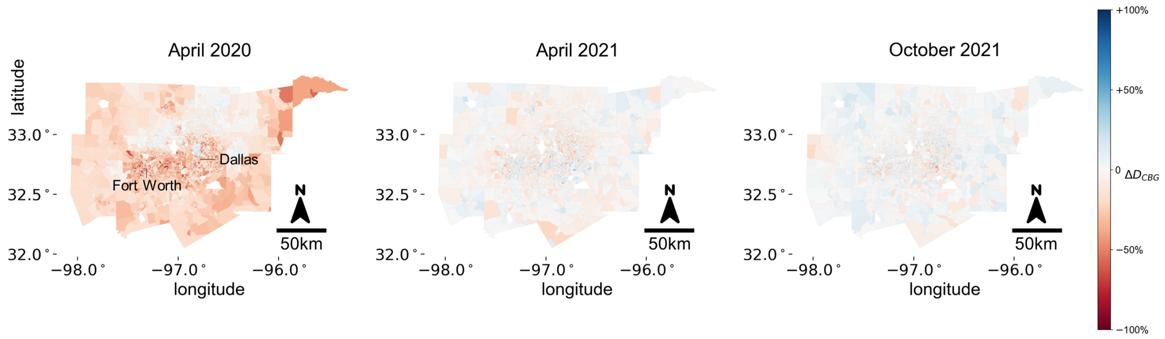

(c) Dallas

Figure S28: ΔD_{CBG} for different time periods in (a) Seattle, (b) Los Angeles, and (c) Dallas. Maps were produced in Python using the TIGER shapefiles from the U.S. Census Bureau [28]

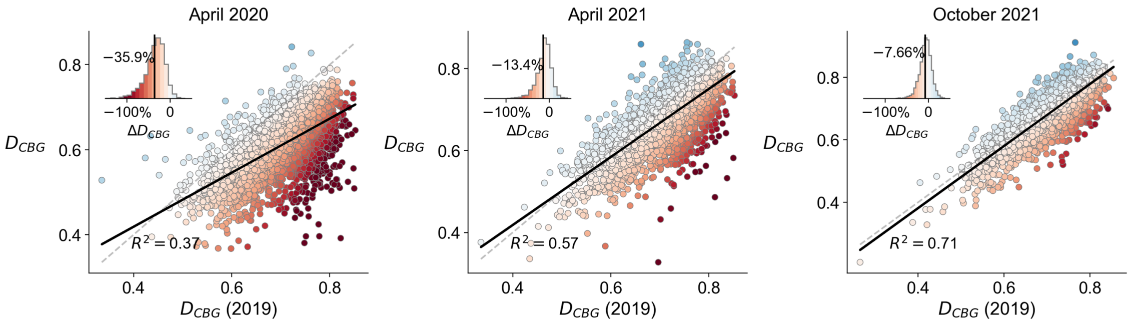

Figure S29: Correlation between D_{CBG} in different timings during the pandemic and the corresponding months in 2019.

- $\{M_{CBG}\}$ is a set of variables that describe the geographical mobility behavior of people living in the corresponding CBG. We use two variables: (i) the radius of gyration of all the places visited by each user, and (ii) the average distance traveled to all places from each individual's home.

The summary statistics of the residential variables are shown in Table S3. Variables that have high correlation amongst eachother, such as '% of people above the age 65', '% of people commuting by car', '% of population between Grades 9 and 12' were removed from the set of variables, and as shown in Figure S30, the correlation among variables are generally low, with the highest magnitude of correlation at $\rho = -0.59$ between '% with Bachelors degree or higher' with '% below Grade 9'. We also checked that the variance inflation factor (VIF) are all between 1 to 5, indicating that there is no significant issue of multicollinearity. Figure S28 shows the differences in ΔD_{CBG} for different periods during the pandemic, and S29 shows the scatter plots of the income diversity in each CBG compared between before the pandemic and during the pandemic at three time points, in Boston CBSA.

To evaluate the relative importance of the three groups of variables, we used the approach proposed by Lindeman, Merenda, and Gold (LMG method) [17]. The LMG method measures the additional R^2 when the variable group is added to the model. Since we have three groups of variables (A,B,C) with six different permutations, thus the contribution of variable group A, for example, is:

$$LMG(A) = \frac{1}{6}\big(2R^2(A) + R^2(A|B) + R^2(A|C) + 2R^2(A|B,C)\big). \quad (8)$$

Figure 3C in the main manuscript shows the relative importance of the three groups of variables for each month, for $D_{CBG}(t)$ and $\Delta D_{CBG}(t)$. Tables S4 to S6 and Tables S7 to S10 show the full regression results for the selected months for D_{CBG} and ΔD_{CBG}, respectively. All significant variables (with $p < 0.01$) in the full model with all variables ({R,M,P}) included, are shown for the other models with partial variables. The model results for D_{CBG} in Tables S4 (pre-pandemic), S5, and S6 (during the pandemic) largely agree with the results in [20], where the residential, mobility, and places variables collectively explain the heterogeneity in diversity well ($R^2 = 0.662$ for October 2021). On the other hand, the differences in diversity ΔD_{CBG} are less well explained by these variables, where the R^2 is at most around 0.3 during the COVID-19 outbreak periods (April, May 2020 and December 2020 and January 2021), as shown in Figure 3C in the main manuscript. This indicates that the decrease in income diversity during the pandemic (especially during the off-peak months) are relatively homogeneous across all sociodemographic segments.

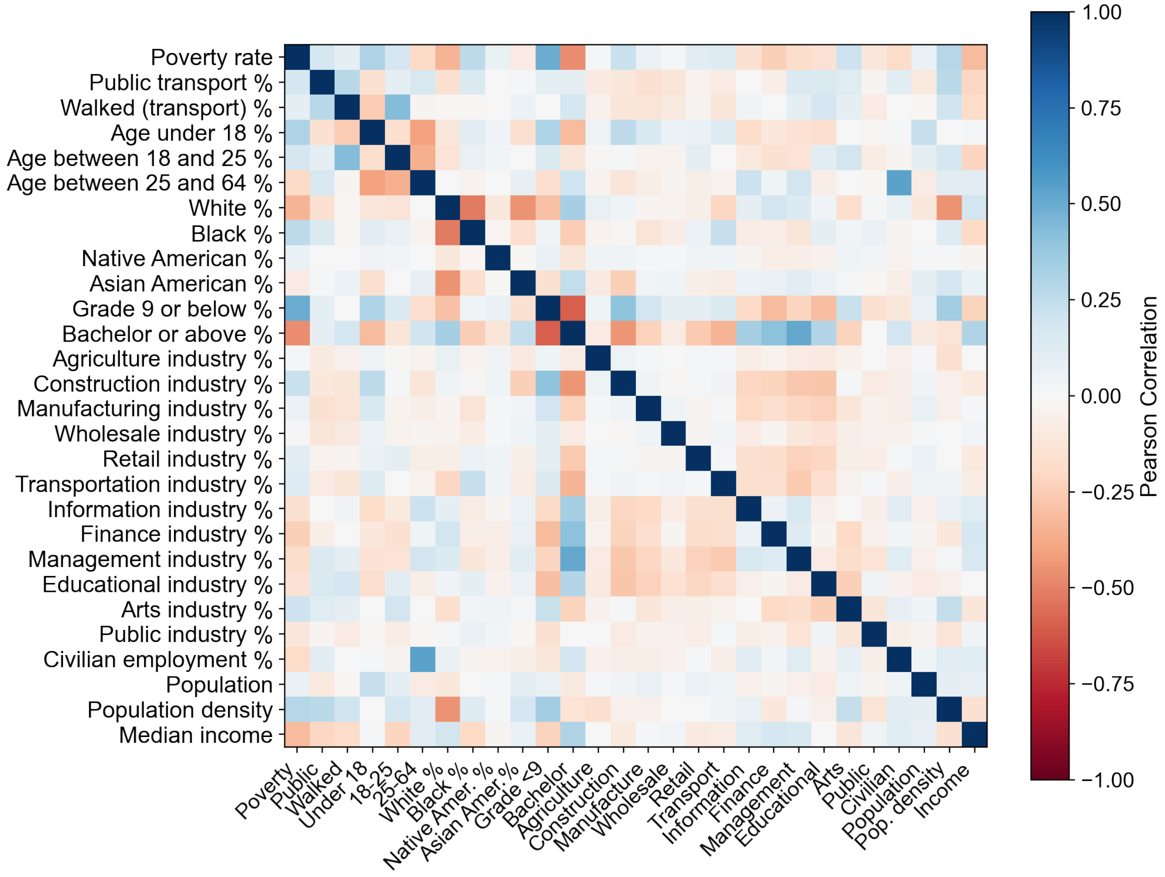

Figure S30: Correlation matrix of CBG based residential variables. Variables that have high correlation amongst eachother, such as '% of people above the age 65', '% of people commuting by car', '% of population between Grades 9 and 12' were removed from the set of variables. The correlation among the resulting variables are generally low, with the highest magnitude of correlation at $\rho = -0.59$ between '% with Bachelors degree or higher' with '% below Grade 9'. 'Management industry' indicates the 'Professional scientific and management and administrative and waste management services' in the American Community Survey categorization.

Table S3: Summary statistics of the residential variables used in the regression models.

Group	Variable	Mean	Std. Dev.	Min.	Max.
Population	$\log_{10}$(Population)	3.152	0.214	0.0	4.245
	$\log_{10}$(Population density)	3.110	0.573	0.0	4.577
Wealth	$\log_{10}$(Median Income)	4.829	0.453	0.0	5.397
	Poverty rate	0.088	0.108	0.0	1.000
Means of transportation to work	Public transport	0.064	0.097	0.0	1.000
	Walked	0.027	0.064	0.0	0.780
Age group	Below 18	0.216	0.086	0.0	0.573
	Between 18 and 25	0.089	0.073	0.0	0.986
	Between 25 and 64	0.551	0.091	0.0	0.955
Race	White	0.643	0.237	0.0	1.000
	Black	0.088	0.151	0.0	1.000
	Native Amerian	0.005	0.017	0.0	0.507
	Asian American	0.113	0.145	0.0	0.942
Educational attainment	Below grade 9	0.058	0.084	0.0	0.650
	Bachelor degree or more	0.376	0.234	0.0	1.000
Industry employment	Agriculture	0.006	0.017	0.0	0.437
	Construction	0.066	0.065	0.0	0.705
	Manufacturing	0.095	0.066	0.0	0.594
	Wholesale	0.030	0.034	0.0	0.378
	Retail	0.105	0.064	0.0	0.619
	Transportation	0.054	0.050	0.0	0.531
	Information	0.030	0.041	0.0	0.429
	Finance	0.071	0.058	0.0	0.680
	Management	0.140	0.082	0.0	0.853
	Educational	0.216	0.095	0.0	0.751
	Arts	0.097	0.068	0.0	0.673
	Public	0.033	0.037	0.0	0.710
Employment status	Civilian in labor force	0.669	0.104	0.0	1.000
PUMA area	Fixed effects	-	-	-	-

	Dependent variable: D_{CBG} (April 2019)						
	{R}	{M}	{P}	{R,M}	{R,P}	{M,P}	{R,M,P}
Constant	0.646***	0.688***	0.688***	0.642***	0.638***	0.688***	0.634***
% Age under 18	-0.011***			-0.011***	-0.009***		-0.009***
% Age 18-25	-0.006***			-0.006***	-0.005***		-0.005***
% Bachelor or more	-0.012***			-0.011***	-0.007***		-0.007***
% Grade 9 or below	-0.003***			-0.003***	-0.003***		-0.003***
% Civilian employed	0.006***			0.006***	0.005***		0.005***
% Finance industry	-0.003***			-0.003***	-0.002***		-0.002***
% Manufacturing industry	-0.003***			-0.003***	-0.002***		-0.002***
% Public industry	0.002***			0.001***	0.001***		0.001***
% Wholesale industry	-0.002***			-0.002***	-0.002***		-0.002***
Population density	0.002***			0.006***	-0.000		0.003***
Median income	-0.002***			-0.003***	-0.003***		-0.003***
Population	0.000			-0.001**	0.003***		0.002***
% Black	-0.002**			-0.002***	-0.001**		-0.002***
Poverty rate	-0.002***			-0.002***	-0.002***		-0.002***
% Public transportation	-0.004***			-0.003***	-0.004***		-0.004***
Traveled distance		0.003***		0.007***		0.004***	0.006***
Radius of gyration		-0.006***		0.002***		-0.001	0.003***
Bubble Tea			0.003***		0.002***	0.003***	0.002***
Sports Bar			0.002***		0.002***	0.002***	0.002***
Auto Workshop			0.002***		0.001***	0.002***	0.001***
Dim Sum			0.002***		0.001***	0.002***	0.001***
Korean			0.001		0.001***	0.001	0.001***
Piano Bar			0.003***		0.001***	0.003***	0.001***
Platform			0.001		0.001***	0.001	0.001***
Pub			0.003***		0.001***	0.003***	0.001***
Veterinarians			0.002***		0.001***	0.002***	0.001***
Video Store			0.001***		0.001***	0.001***	0.001***
Language School			-0.003***		-0.002***	-0.003***	-0.001***
Resort			-0.003***		-0.001***	-0.003***	-0.001***
Basketball			-0.004***		-0.002***	-0.004***	-0.002***
Lodge			-0.004***		-0.002***	-0.004***	-0.002***
Music School			-0.002***		-0.002***	-0.002***	-0.002***
Pilates Studio			-0.005***		-0.002***	-0.005***	-0.002***
South Indian			-0.006***		-0.002***	-0.006***	-0.002***
Wine Bar			-0.004***		-0.002***	-0.004***	-0.002***
Volleyball Court			-0.008***		-0.003***	-0.008***	-0.003***
PUMA Fixed Effects	Yes	No	No	Yes	Yes	No	Yes
Observations	17,824	17,824	17,824	17,824	17,824	17,824	17,824
R^2	0.703	0.002	0.422	0.706	0.735	0.423	0.737
Adjusted R^2	0.699	0.002	0.403	0.702	0.722	0.404	0.725
Note:							*p<0.1; **p<0.05; ***p<0.01

Table S4: Regression results for D_{CBG} for April 2019. The statistical tests were two-sided.

	Dependent variable: D_{CBG} (April 2020)						
	{R}	{M}	{P}	{R,M}	{R,P}	{M,P}	{R,M,P}
Constant	0.565***	0.605***	0.605***	0.563***	0.561***	0.605***	0.558***
% Age under 18	-0.007***			-0.007***	-0.005***		-0.005***
% Age 18-25	-0.004***			-0.004***	-0.003***		-0.003***
% Age 25-64	-0.003***			-0.004***	-0.003***		-0.003***
% Bachelor or more	-0.006***			-0.005***	-0.003***		-0.002**
% Grade 9 or below	-0.004***			-0.004***	-0.003***		-0.003***
% Civilian employed	0.005***			0.004***	0.004***		0.004***
% Public industry	0.002***			0.002***	0.002***		0.002***
% Wholesale industry	-0.001**			-0.001***	-0.001***		-0.001***
Population density	-0.001**			0.003***	-0.002***		0.002***
Median income	-0.001***			-0.002***	-0.002***		-0.002***
Poverty ratio	-0.003***			-0.002***	-0.003***		-0.003***
% Public transportation	-0.006***			-0.006***	-0.006***		-0.005***
Traveled distance		0.012***		0.008***		0.007***	0.008***
Bubble Tea			0.002***		0.001***	0.002***	0.001***
Burgers			0.001***		0.001***	0.001**	0.001***
Coffee Shop			0.001**		0.001***	0.001**	0.001***
Discount Store			0.003***		0.001***	0.003***	0.001***
Theme Park			0.001*		0.001***	0.001	0.001***
Water Park			0.001*		0.001***	0.001*	0.001***
Event Space			-0.001**		-0.001***	-0.001**	-0.001***
Historic Site			-0.001**		-0.001***	-0.001**	-0.001***
Apparel			-0.002***		-0.001***	-0.002***	-0.001***
Baseball			-0.002***		-0.001***	-0.002***	-0.001***
Food & Drink			-0.004***		-0.001**	-0.004***	-0.001***
Language School			-0.003***		-0.002***	-0.002***	-0.001***
Lodge			-0.002***		-0.002***	-0.002***	-0.001***
Music School			-0.002***		-0.001***	-0.002***	-0.001***
Hostel			-0.004***		-0.002***	-0.003***	-0.002***
Pilates Studio			-0.005***		-0.002***	-0.005***	-0.002***
Resort			-0.003***		-0.002***	-0.003***	-0.002***
South Indian			-0.004***		-0.002***	-0.004***	-0.002***
Volleyball Court			-0.006***		-0.003***	-0.006***	-0.003***
PUMA Fixed Effects	Yes	No	No	Yes	Yes	No	Yes
Observations	17,824	17,824	17,824	17,824	17,824	17,824	17,824
R^2	0.582	0.025	0.346	0.585	0.613	0.350	0.615
Adjusted R^2	0.576	0.025	0.325	0.580	0.594	0.329	0.597
Note:							*p<0.1; **p<0.05; ***p<0.01

Table S5: Regression results for D_{CBG} for April 2020. The statistical tests were two-sided.

	Dependent variable: D_{CBG} (October 2021)						
	{R}	{M}	{P}	{R,M}	{R,P}	{M,P}	{R,M,P}
Constant	0.628***	0.670***	0.670***	0.623***	0.620***	0.670***	0.615***
% Age under 18	-0.012***			-0.012***	-0.010***		-0.010***
% Age 18-25	-0.007***			-0.007***	-0.007***		-0.007***
% Bachelor or more	-0.014***			-0.013***	-0.009***		-0.008***
% Grade 9 or below	-0.004***			-0.004***	-0.005***		-0.004***
% Civilian employed	0.007***			0.007***	0.006***		0.006***
% Public industry	0.002***			0.002***	0.002***		0.002***
Population density	0.002**			0.007***	-0.000		0.004***
Median income	-0.003***			-0.003***	-0.003***		-0.003***
Population	-0.000			-0.001***	0.003***		0.002***
% Public transportation	-0.006***			-0.005***	-0.006***		-0.005***
Traveled distance		0.008***		0.008***		0.004***	0.007***
Radius of gyration		-0.003***		0.003***		0.000	0.004***
Billiards			0.003***		0.002***	0.003***	0.002***
Bubble Tea			0.003***		0.002***	0.003***	0.002***
Library			0.002***		0.002***	0.002***	0.002***
Motel			0.002**		0.002***	0.001**	0.002***
Sports Bar			0.003***		0.002***	0.003***	0.002***
Sushi			0.004***		0.002***	0.004***	0.002***
Auto Workshop			0.002***		0.002***	0.002***	0.001***
City Hall			0.000		0.001***	0.000	0.001***
Golf Driving Range			0.003***		0.001***	0.002***	0.001***
Shopping Plaza			0.003***		0.001***	0.003***	0.001***
Water Park			0.001		0.001***	0.001	0.001***
Basketball			-0.004***		-0.002***	-0.004***	-0.002***
Cycle Studio			-0.003***		-0.002***	-0.003***	-0.002***
Field			-0.002***		-0.002***	-0.002***	-0.002***
Hostel			-0.004***		-0.002***	-0.004***	-0.002***
Lodge			-0.004***		-0.002***	-0.004***	-0.002***
Music School			-0.003***		-0.002***	-0.003***	-0.002***
Pilates Studio			-0.005***		-0.002***	-0.005***	-0.002***
South Indian			-0.006***		-0.002***	-0.006***	-0.002***
Volleyball Court			-0.008***		-0.003***	-0.008***	-0.003***
PUMA Fixed Effects	Yes	No	No	Yes	Yes	No	Yes
Observations	17,824	17,824	17,824	17,824	17,824	17,824	17,824
R^2	0.641	0.005	0.375	0.644	0.675	0.376	0.678
Adjusted R^2	0.636	0.005	0.354	0.639	0.660	0.356	0.662

Note: *p<0.1; **p<0.05; ***p<0.01

Table S6: Regression results for D_{CBG} for October 2021. The statistical tests were two-sided.

	Dependent variable: ΔD_{CBG} (April 2020)						
	{R}	{M}	{P}	{R,M}	{R,P}	{M,P}	{R,M,P}
Constant	-0.124***	-0.118***	-0.118***	-0.122***	-0.120***	-0.118***	-0.118***
% Age -18	0.005***			0.005***	0.005***		0.004***
% Age 18-25	0.003***			0.003***	0.003***		0.003***
% over Bachelor	0.008***			0.008***	0.007***		0.007***
% Manufacturing industry	0.002***			0.002***	0.002***		0.002***
Population	-0.001			-0.001*	-0.004***		-0.004***
% Public transportation	-0.004***			-0.004***	-0.003***		-0.003***
Travel distance		0.013***		0.003***		0.006***	0.004***
Radius of gyration		0.007***		-0.003***		-0.001	-0.004***
Basketball			0.003***		0.002***	0.003***	0.002***
Housing Development			0.002***		0.002***	0.002***	0.002***
Non-Profit			0.001**		0.002***	0.001**	0.002***
Pet Store			0.001***		0.002***	0.001**	0.002***
Restaurant			0.002***		0.002***	0.002***	0.002***
Building			-0.002***		-0.002***	-0.002***	-0.002***
Department Store			-0.002***		-0.002***	-0.002***	-0.002***
Funeral Home			-0.002***		-0.002***	-0.002***	-0.002***
Laundromat			-0.003***		-0.002***	-0.003***	-0.002***
Pub			-0.002**		-0.002***	-0.001**	-0.002***
PUMA Fixed Effects	Yes	No	No	Yes	Yes	No	Yes
Observations	17,824	17,824	17,824	17,824	17,824	17,824	17,824
R^2	0.308	0.058	0.270	0.309	0.343	0.273	0.343
Adjusted R^2	0.299	0.058	0.246	0.299	0.312	0.249	0.312
Note:							*p<0.1; **p<0.05; ***p<0.01

Table S7: Regression results for ΔD_{CBG} for April 2020. The statistical tests were two-sided.

	Dependent variable: ΔD_{CBG} *(May 2020)*						
	{R}	{M}	{P}	{R,M}	{R,P}	{M,P}	{R,M,P}
Constant	-0.081***	-0.082***	-0.082***	-0.080***	-0.076***	-0.082***	-0.074***
% Age 25-64	-0.002***			-0.002***	-0.002***		-0.002***
% Finance industry	0.002***			0.002***	0.002***		0.002***
Population density	-0.004***			-0.004***	-0.002***		-0.003***
% Black	0.004***			0.004***	0.004***		0.004***
% Public transportation	-0.004***			-0.004***	-0.004***		-0.004***
Radius of gyration		0.006***		-0.002**		0.000	-0.002***
Engineering			0.002***		0.002***	0.002***	0.002***
Basketball			0.002***		0.001***	0.002***	0.001***
Coffee Shop			0.000		0.001***	0.000	0.001***
Medical School			0.000		0.001***	0.000	0.001***
Music School			0.001**		0.001***	0.001**	0.001***
Non-Profit			0.001**		0.001***	0.001**	0.001***
Restaurant			0.002***		0.001***	0.002***	0.001***
Classroom			-0.001**		-0.001***	-0.001**	-0.001***
Residence Hall			-0.002***		-0.001***	-0.002***	-0.001***
Pub			-0.001**		-0.002***	-0.001**	-0.002***
PUMA Fixed Effects	Yes	No	No	Yes	Yes	No	Yes
Observations	17,824	17,824	17,824	17,824	17,824	17,824	17,824
R^2	0.316	0.060	0.272	0.316	0.343	0.273	0.343
Adjusted R^2	0.306	0.060	0.248	0.307	0.312	0.249	0.312
Note:							*p<0.1; **p<0.05; ***p<0.01

Table S8: Regression results for ΔD_{CBG} for May 2020. The statistical tests were two-sided.

	Dependent variable: ΔD_{CBG} (December 2020)						
	{R}	{M}	{P}	{R,M}	{R,P}	{M,P}	{R,M,P}
Constant	-0.097***	-0.088***	-0.088***	-0.093***	-0.092***	-0.088***	-0.088***
Population density	-0.003***			-0.005***	-0.002***		-0.004***
% Black	0.002***			0.003***	0.002**		0.003***
% Public transportation	-0.005***			-0.006***	-0.005***		-0.005***
Radius of gyration		0.002***		-0.004***		-0.001	-0.004***
Baseball Field			0.002***		0.002***	0.002***	0.002***
Dentist's Office			0.002***		0.001***	0.002***	0.001***
Frame Store			0.002***		0.001***	0.002***	0.001***
Shop			0.001***		0.001***	0.001***	0.001***
Beer Store			-0.002***		-0.001***	-0.002***	-0.001***
Doctor's Office			-0.001***		-0.001***	-0.001**	-0.001***
Engineering			-0.002***		-0.001***	-0.001***	-0.001***
Bar			-0.002***		-0.001***	-0.002***	-0.001***
Seafood			-0.001		-0.001***	-0.001	-0.001***
Gate			-0.001***		-0.002***	-0.001***	-0.002***
PUMA Fixed Effects	Yes	No	No	Yes	Yes	No	Yes
Observations	17,824	17,824	17,824	17,824	17,824	17,824	17,824
R^2	0.275	0.042	0.248	0.277	0.302	0.248	0.303
Adjusted R^2	0.265	0.042	0.223	0.267	0.269	0.223	0.270
Note:					*p<0.1; **p<0.05; ***p<0.01		

Table S9: Regression results for ΔD_{CBG} for December 2020. The statistical tests were two-sided.

5.2 Pooled model with monthly fixed effects

To obtain a more robust understanding of how each factor (residential, mobility, places visited) explains the diversity of urban encounters throughout the entire period of the study, we tested an alternative version of the model where we pool all the months' data together and include monthly fixed effects. In this model, we will obtain a static estimate for the coefficients. The final regression result for D_{CBG} is shown in Table S11. Similar to previous monthly regression model results, many of the sociodemographic, mobility, and place category variables are significant, with an overall $R^2 = 0.644$ which is similar to Tables S4 – S6. The monthly fixed effects are shown in Figure S31, which agrees with the general trend of income diversity decrease that we observe in all cities (e.g., Figure 1C). The root mean squared error of all samples for each month are shown in Figure S32, and shows peaks during the pandemic waves (April 2020 and early 2021). Figure S33 shows the proportions of explained variance across the three categories of variables in the pooled model, which agrees with the general proportion trend shown in Figure 3B. The regression results using ΔD_{CBG} as the dependent variable are shown in Table S12 and Figures S34, S35, and S36. In summary, the estimated parameter coefficients, proportion of R^2 among the three variable categories, and the temporal trends of the root mean squared error show similar patterns with the results using the monthly regression model setup.

	Dependent variable: ΔD_{CBG} *(January 2021)*						
	{R}	{M}	{P}	{R,M}	{R,P}	{M,P}	{R,M,P}
Constant	-0.070***	-0.080***	-0.080***	-0.070***	-0.071***	-0.080***	-0.070***
% Agriculture industry	0.002***			0.002***	0.002***		0.002***
% Management industry	0.003***			0.003***	0.003***		0.003***
% Retail industry	0.003***			0.003***	0.003***		0.003***
% Black	0.004***			0.004***	0.003***		0.003***
% White	0.003***			0.003***	0.004***		0.004***
% Public transport	-0.005***			-0.005***	-0.004***		-0.004***
Department Store			0.003***		0.003***	0.003***	0.003***
Asian American			0.001***		0.002***	0.001***	0.002***
Bank			0.001***		0.002***	0.001**	0.002***
Cosmetics			0.002***		0.002***	0.002***	0.002***
Grocery Store			0.002***		0.002***	0.002***	0.002***
Building			0.001**		0.001***	0.001**	0.001***
Irish			-0.001**		-0.001***	-0.001**	-0.001***
Donburi			-0.004***		-0.002***	-0.004***	-0.002***
PUMA Fixed Effects	Yes	No	No	Yes	Yes	No	Yes
Observations	17,824	17,824	17,824	17,824	17,824	17,824	17,824
R^2	0.307	0.059	0.279	0.307	0.332	0.280	0.332
Adjusted R^2	0.297	0.059	0.255	0.297	0.301	0.257	0.301

Note: *p<0.1; **p<0.05; ***p<0.01

Table S10: Regression results for ΔD_{CBG} for January 2021. The statistical tests were two-sided.

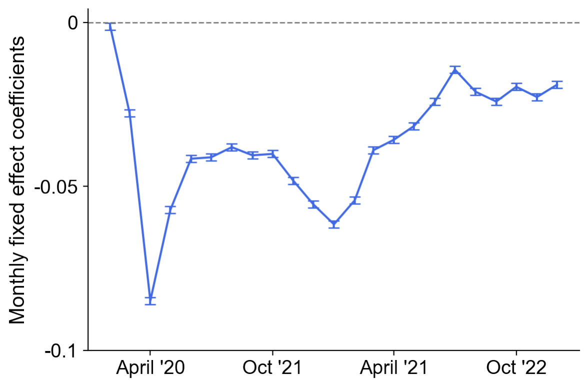

Figure S31: Monthly fixed effects for the pooled model with D_{CBG} as the dependent variable. Data are presented as mean values $+$ / $-$ SEM ($n = 427,776$ POIs across the four cities).

	Dependent variable: D_{CBG}						
	{R}	{M}	{P}	{R,M}	{R,P}	{M,P}	{R,M,P}
Constant	0.709***	0.689***	0.689***	0.711***	0.691***	0.689***	0.692***
% Age under 18	-0.012***			-0.013***	-0.010***		-0.010***
% Age 18-25	-0.007***			-0.007***	-0.006***		-0.006***
% Age 25-64	-0.004***			-0.004***	-0.002***		-0.003***
% Bachelor or more	-0.013***			-0.012***	-0.007***		-0.007***
% Grade 9 or lower	-0.004***			-0.003***	-0.004***		-0.003***
% Civilian employed	0.008***			0.008***	0.007***		0.007***
% Arts industry	0.001***			0.001***	0.000***		0.000***
% Education industry	0.002***			0.002***	0.002***		0.002***
% Finance industry	-0.002***			-0.002***	-0.002***		-0.002***
% Information industry	0.001***			0.001***	0.001***		0.001***
% Manufacturing industry	-0.002***			-0.002***	-0.002***		-0.002***
% Management industry	-0.001***			-0.001***	-0.001***		-0.001***
% Public industry	0.002***			0.002***	0.002***		0.002***
% Retail industry	0.000			-0.000	-0.001***		-0.001***
% Transportation industry	-0.000**			-0.000***	-0.000***		-0.001***
% Wholesale industry	-0.002***			-0.002***	-0.002***		-0.002***
Population density	0.003***			0.008***	0.001***		0.005***
Median income	-0.003***			-0.003***	-0.003***		-0.003***
% Asian American	0.004***			0.004***	0.003***		0.003***
% Black	-0.001***			-0.002***	-0.001***		-0.001***
% White	0.004***			0.004***	0.003***		0.003***
Poverty rate	-0.001***			-0.001***	-0.002***		-0.001***
% Public transportation	-0.005***			-0.005***	-0.005***		-0.004***
% Walked	-0.000***			0.001***	-0.001***		0.001***
Travel distance		0.009***		0.008***		0.004***	0.006***
Radius of gyration		-0.003***		0.000		0.000	0.001***
Discount Store			0.004***		0.002***	0.004***	0.002***
Ski Area			0.003***		0.002***	0.003***	0.002***
Sports Bar			0.002***		0.002***	0.002***	0.002***
Sushi			0.003***		0.002***	0.003***	0.002***
Big Box Store			0.001***		0.001***	0.001***	0.001***
Bubble Tea			0.002***		0.001***	0.002***	0.001***
Butcher			0.001***		0.001***	0.001***	0.001***
Dim Sum			0.002***		0.002***	0.002***	0.001***
Drugstore			-0.000		0.001***	-0.000	0.001***
South Indian			-0.005***		-0.002***	-0.005***	-0.002***
Farm			-0.002***		-0.002***	-0.002***	-0.002***
Hostel			-0.004***		-0.002***	-0.003***	-0.002***
Lodge			-0.003***		-0.002***	-0.003***	-0.002***
Pilates Studio			-0.005***		-0.002***	-0.005***	-0.002***
Baseball			-0.002***		-0.002***	-0.002***	-0.002***
Basketball			-0.004***		-0.002***	-0.004***	-0.002***
Wine Bar			-0.004***		-0.002***	-0.004***	-0.002***
Volleyball Court			-0.008***		-0.003***	-0.008***	-0.003***
Observations	427,776	427,776	427,776	427,776	427,776	427,776	427,776
R^2	0.604	0.056	0.414	0.606	0.643	0.416	0.644
Adjusted R^2	0.603	0.056	0.414	0.606	0.642	0.415	0.644
PUMA Fixed Effects	Yes	No	No	Yes	Yes	No	Yes
Monthly Fixed Effects	Yes	Yes	Yes	Yes	Yes	Yes	Yes

Note: *p<0.1; **p<0.05; ***p<0.01

Table S11: Regression results for D_{CBG}, all months pooled. The statistical tests were two-sided.

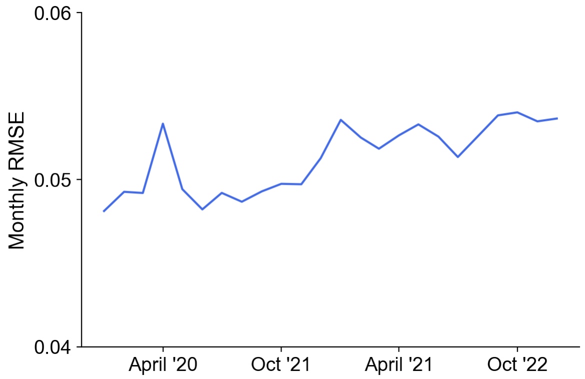

Figure S32: Root mean squared error of the pooled regression model with D_{CBG} as the dependent variable by months.

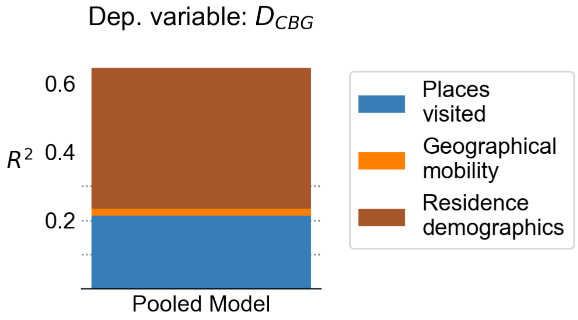

Figure S33: Proportion of explained variance across the three groups of variables in the pooled model with D_{CBG} as the dependent variable.

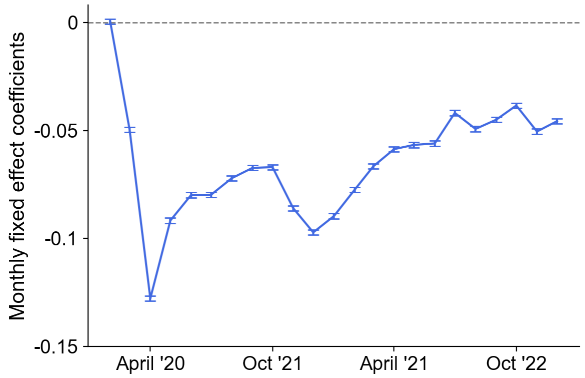

Figure S34: Monthly fixed effects for the pooled model with ΔD_{CBG} as the dependent variable. Data are presented as mean values $+/-$ SEM ($n = 427,776$ POIs across the four cities).

	Dependent variable: ΔD_{CBG}						
	{R}	{M}	{P}	{R,M}	{R,P}	{M,P}	{R,M,P}
Constant	0.015***	0.009***	0.009***	0.015***	0.014***	0.009***	0.014***
% Age 25-64	-0.001***			-0.001***	-0.000***		-0.000***
% Bachelor or more	-0.003***			-0.003***	-0.002***		-0.002***
% Grade 9 or below	-0.001***			-0.001***	-0.001***		-0.001***
% Civilian employed	0.001***			0.001***	0.001***		0.001***
% Agriculture industry	0.000***			0.000***	0.000***		0.000***
% construction industry	0.001***			0.001***	0.001***		0.001***
% Educational industry	0.001***			0.001***	0.001***		0.001***
% Finance industry	0.001***			0.001***	0.000***		0.000***
% Information industry	0.000***			0.000***	0.000***		0.000***
% Manufacturing industry	0.001***			0.001***	0.001***		0.001***
% Management industry	0.001***			0.001***	0.001***		0.001***
% Retail industry	0.002***			0.001***	0.001***		0.001***
% Transportation industry	0.001***			0.001***	0.001***		0.001***
% Wholesale industry	0.000***			0.000***	0.000***		0.000***
Population density	-0.002***			-0.001***	-0.001***		-0.001***
Median income	-0.001***			-0.001***	-0.001***		-0.001***
% Asian American	0.001***			0.001***	0.001***		0.001***
% Black	0.002***			0.002***	0.002***		0.002***
% White	0.001***			0.001***	0.001***		0.001***
Poverty rate	0.001***			0.001***	0.001***		0.001***
% Public transportation	-0.006***			-0.005***	-0.004***		-0.004***
% Walked	-0.002***			-0.002***	-0.001***		-0.001***
Radius of gyration		0.003***		0.002***		0.001***	0.001***
Baseball Field			0.001***		0.001***	0.001***	0.001***
Bowling Alley			0.000***		0.001***	0.000***	0.001***
Cosmetics			0.001***		0.001***	0.001***	0.001***
Discount Store			0.001***		0.001***	0.001***	0.001***
Grocery Store			0.001***		0.001***	0.001***	0.001***
Laser Tag			0.000***		0.001***	0.000***	0.001***
Non-Profit			0.001***		0.001***	0.001***	0.001***
Pawn Shop			0.001***		0.001***	0.001***	0.001***
Shopping Plaza			0.001***		0.001***	0.001***	0.001***
Tex-Mex			0.002***		0.001***	0.002***	0.001***
Carpet Store			-0.000***		-0.001***	-0.000***	-0.001***
Ethiopian			-0.001***		-0.001***	-0.001***	-0.001***
Event Space			-0.000***		-0.001***	-0.000***	-0.001***
Hostel			-0.001***		-0.001***	-0.001***	-0.001***
Irish			-0.000***		-0.001***	-0.000***	-0.001***
Korean			-0.002***		-0.001***	-0.002***	-0.001***
Seafood			-0.001***		-0.001***	-0.001***	-0.001***
Speakeasy			-0.002***		-0.001***	-0.002***	-0.001***
Wine Bar			-0.001***		-0.001***	-0.001***	-0.001***
PUMA Fixed Effects	Yes	No	No	Yes	Yes	No	Yes
Monthly Fixed Effects	Yes	Yes	Yes	Yes	Yes	Yes	Yes
Observations	427,776	427,776	427,776	427,776	427,776	427,776	427,776
R^2	0.271	0.188	0.260	0.271	0.278	0.261	0.278
Adjusted R^2	0.270	0.188	0.259	0.271	0.277	0.260	0.277
Note:							*p<0.1; **p<0.05; ***p<0.01

Table S12: Regression results for ΔD_{CBG}, all months pooled. The statistical tests were two-sided.

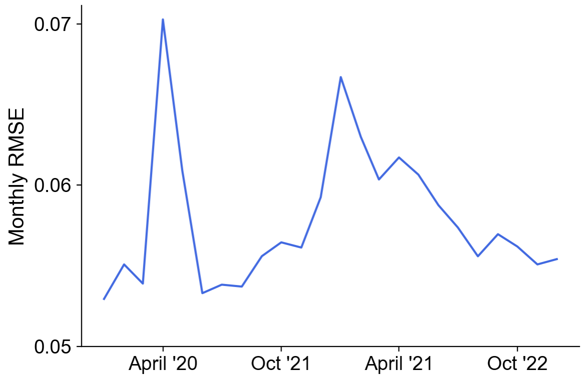

Figure S35: Root mean squared error of the pooled regression model with ΔD_{CBG} as the dependent variable by months.

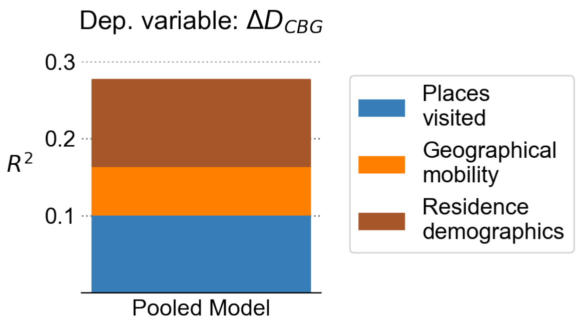

Figure S36: Proportion of explained variance across the three groups of variables in the pooled model with ΔD_{CBG} as the dependent variable.

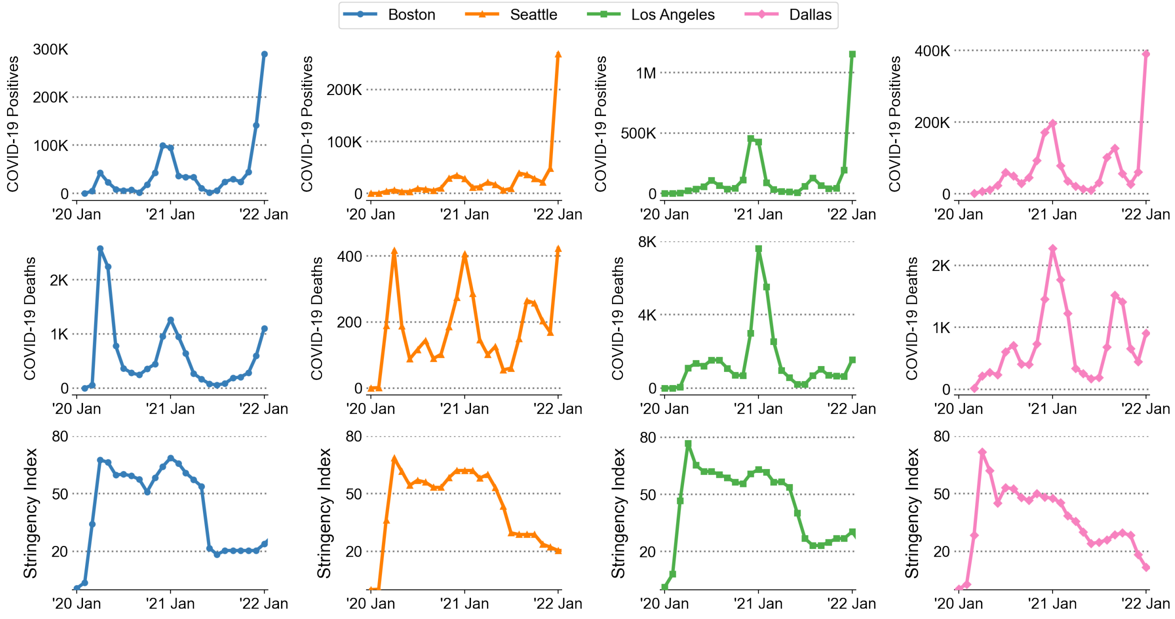

Figure S37: Number of monthly COVID-19 cases (top row), COVID-19 deaths (middle row), and stringency index (bottom row) in each of the CBSAs.

6 COVID-19 intensity and segregation

An interesting aspect of the COVID-19 pandemic was its asynchronicity in terms of outbreaks (number of cases and deaths) and the strictness of implemented policies. To further understand the differences in decreased income diversity across CBSAs, we build simple linear regression models with the form:

$$\Delta D_{CBSA}(t) \sim Cases_{CBSA}(t) + Deaths_{CBSA}(t) + Cases_{US}(t) + Deaths_{US}(t) + SI_{CBSA}(t), \quad (9)$$

where $Cases_{CBSA}(t)$, $Deaths_{CBSA}(t)$, $Cases_{US}(t)$, and $Deaths_{US}(t)$ denote the number of cases and deaths in the corresponding CBSA and the entire USA on time t, which is aggregated monthly.

Data about the number of cases and deaths in each CBSA and for the entire USA were collected from the New York Times Github page[6]. The data were provided on the county scale and for each day, and were aggregated into monthly values for each CBSA. The number of cases and deaths for the four CBSAs are shown in Figure S37.

The Oxford Covid-19 Government Response Tracker (OxCGRT) [7] collects systematic information on policy measures that governments have taken to tackle COVID-19. The different policy responses are tracked since 1 January 2020, cover more than 180 countries and are coded into 23 indicators, such as school closures, travel restrictions, vaccination policy. These policies are recorded on a scale to reflect the extent of government action, and scores are aggregated into a suite of policy indices. The stringency index $SI_{CBSA}(t)$ is a composite metric that measures the strictness of COVID-19 policies calculated using data collected in OxCGRT [8], and are provided at the state levels for the United States. More specifically, the stringency index takes into account:

[6]`https://github.com/nytimes/covid-19-data`

[7]`https://www.bsg.ox.ac.uk/research/research-projects/covid-19-government-response-tracker`

1. closings of schools and universities; scaled from 0 (no measures) to 3 (required closing)
2. closings of workplaces; scaled from 0 to 3
3. cancelling of public events; scaled from 0 to 2
4. limits on gatherings scaled from 0 to 4
5. closing of public transport scaled from 0 to 2
6. orders to ”shelter-in-place” and otherwise confine to the home scaled from 0 to 3
7. restrictions on internal movement between cities/regions scaled between 0 and 2
8. restrictions on international travel scaled from 0 to 4
9. presence of public info campaigns scaled between 0 and 2

More details are provided in the codebook in the github webpage [8]. The stringency index for each CBSA are shown in the bottom row of Figure S37. While all cities had high stringency until late 2020, the rollout of vaccines in early 2021 have significantly lowered the stringency.

6.1 Model estimation results

The regression model results for the effects of COVID-19 intensity on income diversity are shown in Table S13. We observe that the stringency index is significant for all CBSAs with a negative coefficient, which indicates that stricter the COVID-19 policies, the less diverse urban encounters become. In addition to the stringency index, the number of deaths at the CBSA and federal levels are also significant for Boston and Seattle. Both coefficients are negative, which indicate that when the monthly number of deaths are higher, the less diverse urban encounters become. To remove insignificant variables from the model, we tested a more simpler version with the form:

$$\Delta D_{CBSA}(t) \sim Deaths_{CBSA}(t) + SI_{CBSA}(t). \tag{10}$$

The model estimation results are shown in Table S14. The significance of the variables nor their direction are consistent with the first version of the model. The constants for Boston and Los Angeles are significantly negative, indicating that in the hypothetical scenario where there are zero monthly COVID-19 deaths and zero stringency of policies, the income diversity will have a negative change compared to 2019. Given that the scenario where we completely eliminate COVID-19 cases and deaths as well as social distancing policies in the near future with the coronavirus becoming an endemic disease, this result suggests that there could be a long-lasting effect of the pandemic on the income diversity of urban encounters. Regression results when we use only the stringency index is shown in Figure S38.

6.2 Robustness of results via time series modeling

Since the variables used in this model are temporal data, including the decrease in diversity as well as COVID-19 related data, it is important to check stationarity, autocorrelation, and partial autocorrelation, and if applicable test whether such temporal dependencies affect the outcomes of the results.

[8] `https://github.com/OxCGRT/covid-policy-tracker/blob/master/documentation/codebook.md`

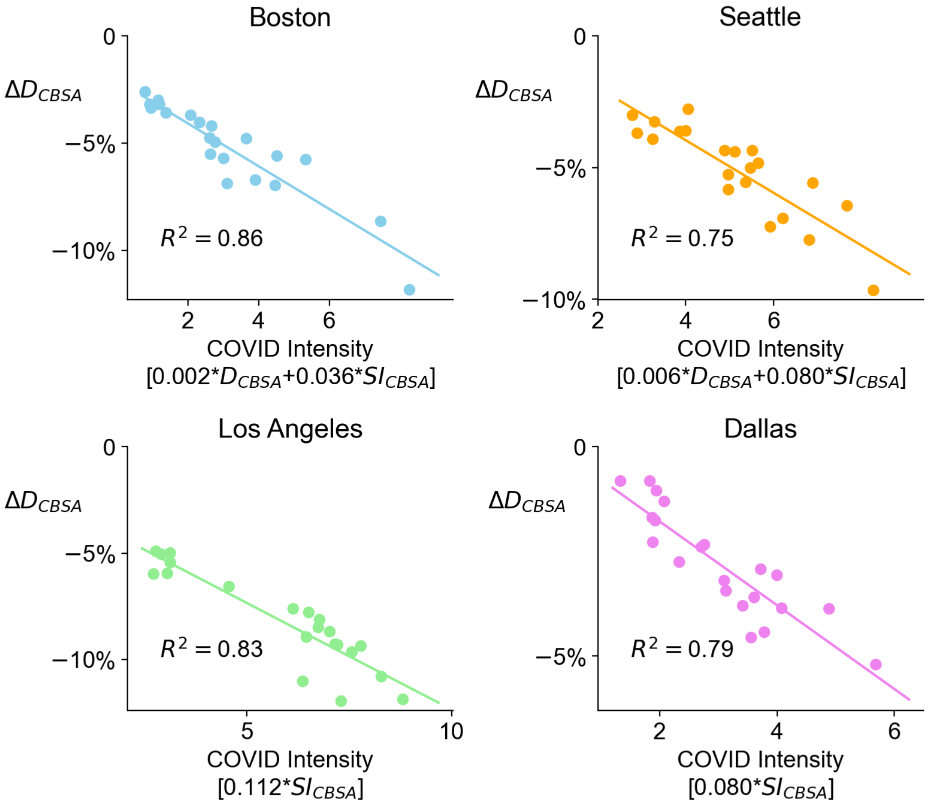

Figure S38: Reduction in income diversity regressed against the stringency index and the death rates due to COVID-19 in each of the CBSAs. For Boston and Seattle, the death rates were significant; for Los Angeles and Dallas the death rate was insignificant.

	Dependent variable: $\Delta D_{CBSA}(t)$			
	Boston	Seattle	Los Angeles	Dallas
Constant	-2.110***	-0.213	-1.127	-0.027
	(0.542)	(0.960)	(0.886)	(0.665)
Positives (CBSA)	0.000	0.000	-0.000	-0.000
	(0.000)	(0.000)	(0.000)	(0.000)
Positives (USA)	-0.000*	-0.000**	-0.000	0.000
	(0.000)	(0.000)	(0.000)	(0.000)
Deaths (CBSA)	-0.003***	-0.015***	0.000	0.001
	(0.000)	(0.003)	(0.000)	(0.001)
Deaths (USA)	0.000**	0.000***	-0.000	0.000
	(0.000)	(0.000)	(0.000)	(0.000)
Stringency Index (CBSA)	-0.039***	-0.080***	-0.128***	-0.077***
	(0.011)	(0.017)	(0.014)	(0.016)
Observations	21	21	21	21
R^2	0.906	0.853	0.914	0.820
Adjusted R^2	0.875	0.805	0.885	0.760
Note:				*p<0.1; **p<0.05; ***p<0.01

Table S13: Regression results for $\Delta D_{CBSA}(t)$ using COVID-19 intensity and policy measures. The statistical tests were two-sided.

	Dependent variable: $\Delta D_{CBSA}(t)$			
	Boston	Seattle	Los Angeles	Dallas
Constant	-2.073***	0.041	-2.343***	0.215
	(0.500)	(0.727)	(0.682)	(0.460)
Deaths (CBSA)	-0.002***	-0.007***	-0.000	0.000
	(0.000)	(0.002)	(0.000)	(0.000)
Stringency Index (CBSA)	-0.036***	-0.081***	-0.112***	-0.080***
	(0.012)	(0.014)	(0.014)	(0.010)
Observations	21	21	21	21
R^2	0.863	0.752	0.827	0.786
Adjusted R^2	0.847	0.725	0.807	0.762
Note:				*p<0.1; **p<0.05; ***p<0.01

Table S14: Regression results for $\Delta D_{CBSA}(t)$ using only COVID-19 local deaths and policy strictness measures. The statistical tests were two-sided.

	$\Delta D_{CBSA}(t)$		
	ADF Statistic	p-value	Stationary?
Boston	-13.43	4.02^{-25}	Yes
Seattle	-0.66	0.85	No
Los Angeles	-2.06	0.25	No
Dallas	-1.06	0.72	No

Table S15: Augmented Dickey Fuller test for $\Delta D_{CBSA}(t)$.

To check the stationarity of $\Delta D_{CBSA}(t)$, we conduct the Augmented Dickey Fuller (ADF) test [21]. Table S15 shows the ADF statistic, p-value, and whether the time series is determined to be stationary or not. The results show that except for Boston, the time series are non-stationary, thus we need to do some differencing. Figure S39 shows the autocorrelation and partial autocorrelation of the data $\Delta D_{CBSA}(t)$ under no differencing and 1st order differencing for the four CBSAs. We observe that for all three cities except Boston (which requires no differencing), 1st order differencing is enough to obtain no autocorrelation beyond 1 time step.

To model the temporal dynamics, we apply an ARIMA(p, d, q) model with covariates (number of local COVID-19 deaths and local stringency index). The model parameters p, d, q of the ARIMA model, each corresponding to the autoregressive term (or the lag of the dependent variable, number of differencing needed for stationary time series, and the lagged forecast error term, respectively. For Boston, the ADF test shows that no differencing is needed, thus $d = 0$. Under no differencing, ARIMA(1,0,0) and ARIMA(0,0,1) were tested for Boston and only the MA term was significant (shown in first column in Table S16). Using the ARIMA(0,0,1) model for Boston, both the he number of local deaths and the local stringency index were statistically significant with $p < 0.01$, indicating robustness of the OLS results in Table S14. For the other three cities, since $d = 1$ was determined using the ADF test, ARIMA(1,1,0), ARIMA(0,1,1), and ARIMA(1,1,1) were modeled and the statistical significance of autoregressive and moving average terms were tested. For Seattle, as shown in the second column in Table S16, the moving average term showed statistical significance with $p < 0.05$ and both the number of local deaths and the local stringency index were also statistically significant with $p < 0.05$, indicating robustness of the OLS results in Table S14. For Los Angeles and Dallas, both the autoregressive and moving average terms were statistically insignificant, indicating that the dependent variable can be modeled using OLS instead of time series models. To summarize, for Boston and Seattle ΔD_{CBSA} could be modeled as a moving average process but the coefficients and significance of the independent variables were consistent with the OLS results in Table S14. For Los Angeles and Dallas, the temporal components were insignificant, therefore the results in Table S14 are robust.

7 Software

Analysis was conducted using Python, Jupyter Lab, and the following libraries and software:

- `NumPy` [9] for general computation on Python.
- `Pandas` [19] for loading, transforming, and analyzing data tables.

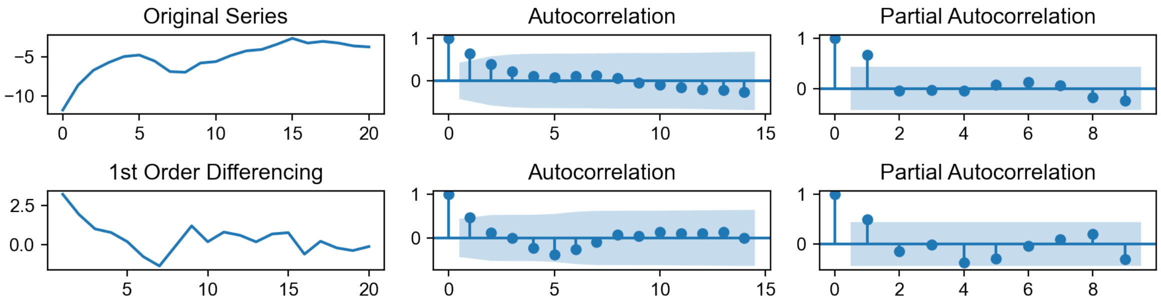

(a) Boston

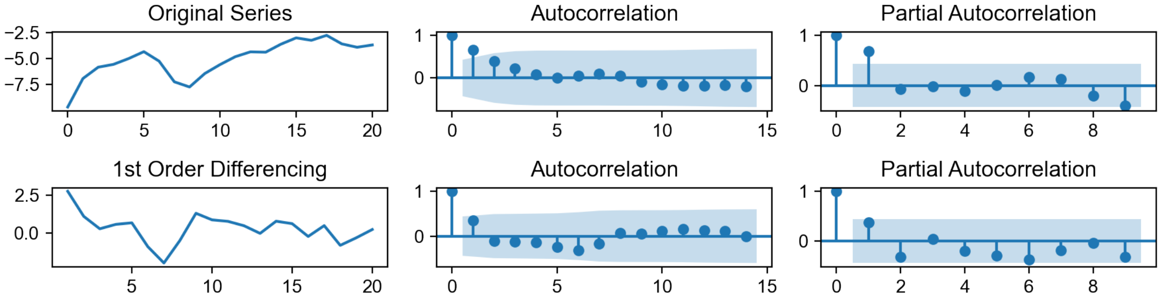

(b) Seattle

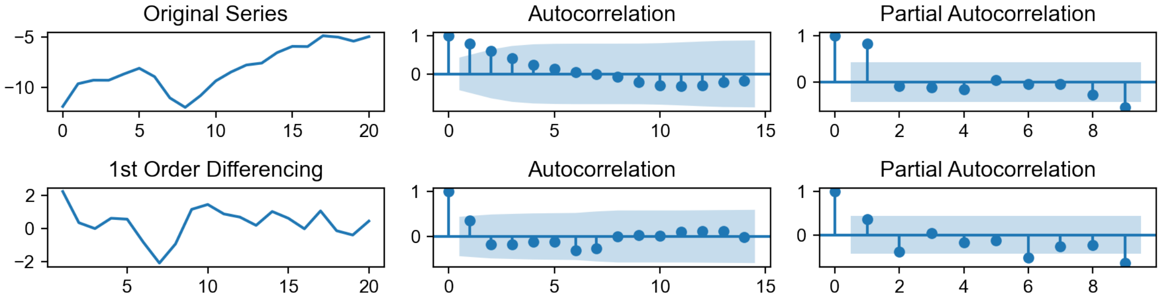

(c) Los Angeles

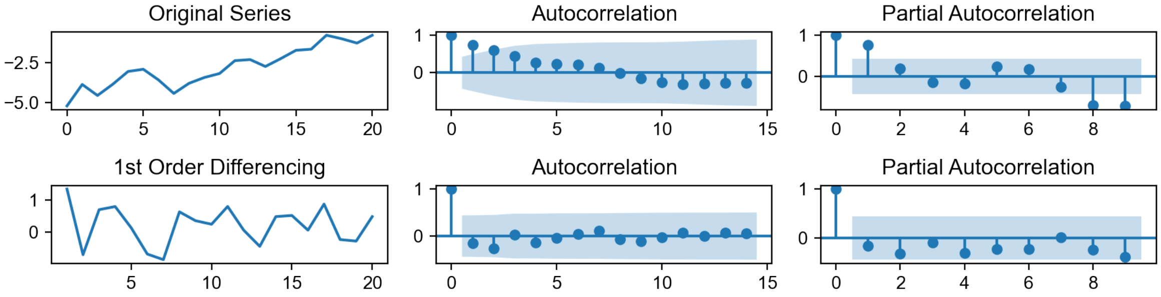

(d) Dallas

Figure S39: Autocorrelation and partial autocorrelation of original series and 1st order of differencing of $\Delta D_{CBSA}(t)$ for (a) Boston, (b) Seattle, (c) Los Angeles, and (d) Dallas. Error bands for autocorrelation and partial autocorrelation show the 95% confidence intervals.

	Dep. variable: $\Delta D_{CBSA}(t)$			
	Boston	Seattle	Los Angeles	Dallas
Best model	ARIMA(0,0,1)	ARIMA(0,1,1)	None	None
Constant	-2.073***	-	-	-
Deaths (CBSA)	-0.002***	-0.003**	-	-
Stringency Index (CBSA)	-0.036***	-0.070**	-	-
Autoregressive 1 lag	-	-	-	-
Moving average 1 lag	0.630***	0.736**	-	-
σ^2	0.374***	0.458**	-	-
Observations	21	21	-	-
AIC	58.849	49.934	-	-
Note:	*p<0.1; **p<0.05; ***p<0.01			
	Both AR and MA terms were insignificant for Los Angeles and Dallas.			

Table S16: ARIMA regression results for $\Delta D_{CBSA}(t)$ using COVID-19 local deaths and policy strictness measures. The statistical tests were two-sided.

- `Matplotlib` [11] for creating plots and figures.
- `GeoPandas` [16] for spatial analysis and plotting map figures.
- `Statsmodels` [24] for statistical modeling and econometric analysis.
- A Python implementation of the R `Stargazer` multiple regression model creation tool[9] was used to create the regression tables.

Supplementary References

[1] Alberto Aleta, David Martín-Corral, Michiel A Bakker, Ana Pastore y Piontti, Marco Ajelli, Maria Litvinova, Matteo Chinazzi, Natalie E Dean, M Elizabeth Halloran, Ira M Longini Jr, et al. Quantifying the importance and location of sars-cov-2 transmission events in large metropolitan areas. *Proceedings of the National Academy of Sciences*, 119(26):e2112182119, 2022.

[2] Alberto Aleta, David Martin-Corral, Ana Pastore y Piontti, Marco Ajelli, Maria Litvinova, Matteo Chinazzi, Natalie E Dean, M Elizabeth Halloran, Ira M Longini Jr, Stefano Merler, Alex Pentland, Alessandro Vespignani, Esteban Moro, and Yamir Moreno. Modelling the impact of testing, contact tracing and household quarantine on second waves of covid-19. *Nature Human Behaviour*, 4(9):964–971, 2020.

[3] United States Census Bureau. American community survey. `https://www.census.gov/programs-surveys/acs`. Accessed: 2019-12-14.

[9] `https://github.com/mwburke/stargazer`

[4] Serina Chang, Emma Pierson, Pang Wei Koh, Jaline Gerardin, Beth Redbird, David Grusky, and Jure Leskovec. Mobility network models of covid-19 explain inequities and inform reopening. *Nature*, 589(7840):82–87, 2021.

[5] Riccardo Di Clemente, Miguel Luengo-Oroz, Matias Travizano, Sharon Xu, Bapu Vaitla, and Marta C González. Sequences of purchases in credit card data reveal lifestyles in urban populations. *Nature Communications*, 9(1):1–8, 2018.

[6] Nathan Eagle, Michael Macy, and Rob Claxton. Network diversity and economic development. *Science*, 328(5981):1029–1031, 2010.

[7] Foursquare. Foursquare Places. `https://foursquare.com/products/places`, 2020. Accessed: 09-12-2020.

[8] Thomas Hale, Noam Angrist, Rafael Goldszmidt, Beatriz Kira, Anna Petherick, Toby Phillips, Samuel Webster, Emily Cameron-Blake, Laura Hallas, Saptarshi Majumdar, et al. A global panel database of pandemic policies (oxford covid-19 government response tracker). *Nature Human Behaviour*, 5(4):529–538, 2021.

[9] Charles R Harris, K Jarrod Millman, Stéfan J Van Der Walt, Ralf Gommers, Pauli Virtanen, David Cournapeau, Eric Wieser, Julian Taylor, Sebastian Berg, Nathaniel J Smith, et al. Array programming with numpy. *Nature*, 585(7825):357–362, 2020.

[10] Hartwig H. Hochmair, Levente Juhász, and Sreten Cvetojevic. Progress in Location Based Services 2018. *Lecture Notes in Geoinformation and Cartography*, pages 293–313, 2017.

[11] John D Hunter. Matplotlib: A 2d graphics environment. *Computing in Science & Engineering*, 9(03):90–95, 2007.

[12] Ruth F Hunter, Leandro Garcia, Thiago Herick de Sa, Belen Zapata-Diomedi, Christopher Millett, James Woodcock, Esteban Moro, et al. Effect of covid-19 response policies on walking behavior in us cities. *Nature Communications*, 12(1):1–9, 2021.

[13] Infogroup. ReferenceUSA Business Historical Data Files, 2014.

[14] Shan Jiang, Gaston A Fiore, Yingxiang Yang, Joseph Ferreira Jr, Emilio Frazzoli, and Marta C González. A review of urban computing for mobile phone traces: current methods, challenges and opportunities. In *Proceedings of the 2nd ACM SIGKDD international workshop on Urban Computing*, pages 1–9, 2013.

[15] Shan Jiang, Yingxiang Yang, Siddharth Gupta, Daniele Veneziano, Shounak Athavale, and Marta C González. The timegeo modeling framework for urban mobility without travel surveys. *Proceedings of the National Academy of Sciences*, 113(37):E5370–E5378, 2016.

[16] K Jordahl. Geopandas: Python tools for geographic data. *URL: https://github.com/geopandas/geopandas*, 3, 2014.

[17] Richard H Lindeman, PF Merenda, and RZ Gold. Introduction to bivariate and multivariate analysis, glenview, il. *Scott: Foresman and company*, 119, 1980.

[18] Lorenzo Lucchini, Simone Centellegher, Luca Pappalardo, Riccardo Gallotti, Filippo Privitera, Bruno Lepri, and Marco De Nadai. Living in a pandemic: changes in mobility routines, social activity and adherence to covid-19 protective measures. *Scientific reports*, 11(1):1–12, 2021.

[19] Wes McKinney et al. pandas: a foundational python library for data analysis and statistics. *Python for high performance and scientific computing*, 14(9):1–9, 2011.

[20] Esteban Moro, Dan Calacci, Xiaowen Dong, and Alex Pentland. Mobility patterns are associated with experienced income segregation in large us cities. *Nature Communications*, 12(1):1–10, 2021.

[21] Rizwan Mushtaq. Augmented dickey fuller test, 2011.

[22] Nolan E Phillips, Brian L Levy, Robert J Sampson, Mario L Small, and Ryan Q Wang. The social integration of american cities: Network measures of connectedness based on everyday mobility across neighborhoods. *Sociological Methods & Research*, page 0049124119852386, 2019.

[23] Matthew J Salganik. *Bit by bit: Social research in the digital age*. Princeton University Press, 2019.

[24] Skipper Seabold and Josef Perktold. Statsmodels: Econometric and statistical modeling with python. In *Proceedings of the 9th Python in Science Conference*, volume 57, page 61. Austin, TX, 2010.

[25] Chaoming Song, Tal Koren, Pu Wang, and Albert-László Barabási. Modelling the scaling properties of human mobility. *Nature Physics*, 6(10):818–823, 2010.

[26] Seth E. Spielman and Alex Singleton. Studying Neighborhoods Using Uncertain Data from the American Community Survey: A Contextual Approach. *Annals of the Association of American Geographers*, 105(5):1003–1025, 8 2015.

[27] Eric Tsetsi and Stephen A Rains. Smartphone internet access and use: Extending the digital divide and usage gap. *Mobile Media & Communication*, 5(3):239–255, 2017.

[28] United States Census Bureau. Tiger data products guide. `https://www.census.gov/programs-surveys/geography/guidance/tiger-data-products-guide.html`. Accessed: 2023-02-27.

[29] Qi Wang, Nolan Edward Phillips, Mario L Small, and Robert J Sampson. Urban mobility and neighborhood isolation in america's 50 largest cities. *Proceedings of the National Academy of Sciences*, 115(30):7735–7740, 2018.

[30] Longgang Xiang, Meng Gao, and Tao Wu. Extracting stops from noisy trajectories: A sequence oriented clustering approach. *ISPRS International Journal of Geo-Information*, 5(3):29, 2016.
