# Supplementary material for: Behavioral changes during the COVID-19 pandemic decreased income diversity of urban encounters
Source: Nat Commun. 2023 Apr 21;14:2310. doi: 10.1038/s41467-023-37913-y (PMC10120472; doi:10.1038/s41467-023-37913-y)
Supplement: Supplementary file 3 — Reporting Summary [file 41467_2023_37913_MOESM3_ESM.pdf]

nature portfolio

Corresponding author(s): Takahiro Yabe, Esteban Moro

Last updated by author(s): Feb 27, 2023

# Reporting Summary

Nature Portfolio wishes to improve the reproducibility of the work that we publish. This form provides structure for consistency and transparency in reporting. For further information on Nature Portfolio policies, see our Editorial Policies and the Editorial Policy Checklist.

## Statistics

For all statistical analyses, confirm that the following items are present in the figure legend, table legend, main text, or Methods section.

| n/a | Confirmed | |
| --- | --- | --- |
| ☐ | ☒ | The exact sample size (*n*) for each experimental group/condition, given as a discrete number and unit of measurement |
| ☒ | ☐ | A statement on whether measurements were taken from distinct samples or whether the same sample was measured repeatedly |
| ☐ | ☒ | The statistical test(s) used AND whether they are one- or two-sided<br>*Only common tests should be described solely by name; describe more complex techniques in the Methods section.* |
| ☐ | ☒ | A description of all covariates tested |
| ☒ | ☐ | A description of any assumptions or corrections, such as tests of normality and adjustment for multiple comparisons |
| ☐ | ☒ | A full description of the statistical parameters including central tendency (e.g. means) or other basic estimates (e.g. regression coefficient) AND variation (e.g. standard deviation) or associated estimates of uncertainty (e.g. confidence intervals) |
| ☐ | ☒ | For null hypothesis testing, the test statistic (e.g. *F*, *t*, *r*) with confidence intervals, effect sizes, degrees of freedom and *P* value noted<br>*Give P values as exact values whenever suitable.* |
| ☒ | ☐ | For Bayesian analysis, information on the choice of priors and Markov chain Monte Carlo settings |
| ☒ | ☐ | For hierarchical and complex designs, identification of the appropriate level for tests and full reporting of outcomes |
| ☐ | ☒ | Estimates of effect sizes (e.g. Cohen's *d*, Pearson's *r*), indicating how they were calculated |

*Our web collection on statistics for biologists contains articles on many of the points above.*

## Software and code

Policy information about availability of computer code

Data collection

No special software was used to collect the data.

Data analysis

Data analysis was conducted using different python libraries. Here is a list of references, included in the Supplementary Material.
- Charles R Harris, K Jarrod Millman, Stefan J Van Der Walt, Ralf Gommers, Pauli Virtanen, David Cournapeau, Eric Wieser, Julian Taylor, Sebastian Berg, Nathaniel J Smith, et al. Array programming with numpy. Nature, 585(7825):357–362, 2020. (version 1.24.2)
- John D Hunter. Matplotlib: A 2d graphics environment. Computing in Science & Engineering, 9(03):90–95, 2007. (version 3.7.0)
- Wes McKinney et al. pandas: a foundational python library for data analysis and statistics. Python for high performance and scientific computing, 14(9):1–9, 2011. (version 1.5.3)
- K Jordahl. Geopandas: Python tools for geographic data. URL: https://github.com/geopandas/geopandas, 3, 2014.(version 0.12.2)
- Skipper Seabold and Josef Perktold. Statsmodels: Econometric and statistical modeling with python. In Proceedings of the 9th Python in Science Conference, volume 57, page 61. Austin,TX, 2010. (version 0.13.5)
- Stargazer implementation in R. URL: https://github.com/mwburke/stargazer (Python implementation of the R stargazer multiple regression model creation tool, version 5.2.3)

Code to reproduce our results in the figures from the aggregated data are available on Github. URL: https://github.com/takayabe0505/incomeDiversity

For manuscripts utilizing custom algorithms or software that are central to the research but not yet described in published literature, software must be made available to editors and reviewers. We strongly encourage code deposition in a community repository (e.g. GitHub). See the Nature Portfolio guidelines for submitting code & software for further information.

# Data

Policy information about availability of data

All manuscripts must include a data availability statement. This statement should provide the following information, where applicable:

- Accession codes, unique identifiers, or web links for publicly available datasets
- A description of any restrictions on data availability
- For clinical datasets or third party data, please ensure that the statement adheres to our policy

The data that support the findings of this study are available from Spectus through their Social Impact program, but restrictions apply to the availability of these data, which were used under the license for the current study and are therefore not publicly available. Information about how to request access to the data and its conditions and limitations can be found in https://spectus.ai/social-impact/ . Data access requests should be submitted through Spectus' Social Impact customer page https://spectus.ai/lp/book-a-demo/. Timely response should be expected from the Sales team at Spectus. Other data including the American Community Survey is available for download at https://data.census.gov/, and Tiger shapefiles can be downloaded from the US Census Bureau https://www.census.gov/programs-surveys/geography/guidance/tiger-data-products-guide.html.

# Human research participants

Policy information about studies involving human research participants and Sex and Gender in Research.

| | |
|---|---|
| Reporting on sex and gender | The data contains no information about sex or gender. |
| Population characteristics | Data used are geo-locations from anonymous opted-in devices collected by the company Spectus in four metro areas in the US. Data have been aggregated at the level of places, categories, or census areas where a number of devices are present to prevent de-anonymization. The data is quantitative as it reflects the precise time and geolocation of the anonymous users or metrics from the census. |
| Recruitment | Data used are geo-locations from anonymous opted-in devices collected by the company Spectus in four metro areas in the US. Data have been aggregated at the level of places, categories, or census areas where a number of devices are present to prevent de-anonymization. Visitation information at sensitive places have been removed to protect the privacy of individuals. To minimize the potential bias in the geographical penetration of the users, we have implemented post-stratification techniques. All details about our sampling methods and post-stratification methods can be found in the Supplementary Material. |
| Ethics oversight | Since the data used was anonymized and spatially aggregated at places, categories, or census areas, we were granted an Exemption by the MIT Committee on the Use of Humans as Experimental Subjects (COUHES protocol #1812635935) and its extension #E-2962. |

Note that full information on the approval of the study protocol must also be provided in the manuscript.

# Field-specific reporting

Please select the one below that is the best fit for your research. If you are not sure, read the appropriate sections before making your selection.

☐ Life sciences ☒ Behavioural & social sciences ☐ Ecological, evolutionary & environmental sciences

For a reference copy of the document with all sections, see nature.com/documents/nr-reporting-summary-flat.pdf

# Behavioural & social sciences study design

All studies must disclose on these points even when the disclosure is negative.

| | |
|---|---|
| Study description | The study analyzes geo-location data from anonymous opted-in devices collected by the company Spectus in 4 metro areas in the US to understand the diversity of urban encounters across a three year period during the pandemic. Data have been aggregated at the level of places, categories, or census areas where a number of devices are present to prevent de-anonymization. The data enabled analysis of urban dynamics via quantitative methods. |
| Research sample | The sample of users is described above: anonymous opted-in devices collected by the company Spectus. The study sample was chosen to understand the microscopic behavior of individuals in urban environments at a high spatial and temporal granularity. The four cities were chosen to observe differences between cities with large variability in sociodemographic, geographic, policy, climate, and urban form. To maximize the representativeness of the data, we implemented post-stratification techniques. The details of the methods can be found in the Supplementary Material. |
| Sampling strategy | Mobile phone users whose mobility activity was sufficiently observed in the dataset during the period of interest. See the Methods section and Supplementary Material for the details of the sampling strategy. The sample size and percentage calculation was performed by overlaying the mobile phone location data with census data from the American Community Survey. To minimize |

potential biases in the data sample and the geographical differences in mobile phone penetration rate of users, we implemented post-stratification techniques. Details may be found in the Supplementary Material.

| | |
|---|---|
| Data collection | Data was collected by the company Spectus through different applications on their mobile phones. The researchers were blinded to experimental conditions and the study hypothesis. |
| Timing | Data was collected through January 2019 to January 2022. |
| Data exclusions | No data was excluded |
| Non-participation | Only anonymous opted-in devices were used in the analysis. |
| Randomization | The data collected is observational and does not come from an experiment. Thus, this is not applicable. |

# Reporting for specific materials, systems and methods

We require information from authors about some types of materials, experimental systems and methods used in many studies. Here, indicate whether each material, system or method listed is relevant to your study. If you are not sure if a list item applies to your research, read the appropriate section before selecting a response.

## Materials & experimental systems

| n/a | Involved in the study | |
|---|---|---|
| ☒ | ☐ | Antibodies |
| ☒ | ☐ | Eukaryotic cell lines |
| ☒ | ☐ | Palaeontology and archaeology |
| ☒ | ☐ | Animals and other organisms |
| ☒ | ☐ | Clinical data |
| ☒ | ☐ | Dual use research of concern |

## Methods

| n/a | Involved in the study | |
|---|---|---|
| ☒ | ☐ | ChIP-seq |
| ☒ | ☐ | Flow cytometry |
| ☒ | ☐ | MRI-based neuroimaging |
